# Spatially resolved transcriptomic profiles reveal unique defining molecular features of infiltrative 5ALA-metabolizing cells associated with glioblastoma recurrence

**DOI:** 10.1186/s13073-023-01207-1

**Published:** 2023-07-11

**Authors:** Geoffroy Andrieux, Tonmoy Das, Michaela Griffin, Jakob Straehle, Simon M. L. Paine, Jürgen Beck, Melanie Boerries, Dieter H. Heiland, Stuart J. Smith, Ruman Rahman, Sajib Chakraborty

**Affiliations:** 1 Institute of Medical Bioinformatics and Systems Medicine, Medical Center - University of Freiburg Faculty of Medicine, University of Freiburg, Freiburg, Germany; 2 Systems Cell-Signaling Laboratory, Department of Biochemistry and Molecular Biology, University of Dhaka, Dhaka, Bangladesh; 3 Children’s Brain Tumour Research Centre, Biodiscovery Institute, School of Medicine, University of Nottingham, Nottingham, UK; 4 Department of Neurosurgery, Medical Center - University of Freiburg, Freiburg, Germany; 5 German Cancer Consortium (DKTK) Partner Site Freiburg, German Cancer Research Center (DKFZ), Heidelberg, Germany; 6 Microenvironment and Immunology Research Laboratory, Medical Center - University of Freiburg, Freiburg, Germany; 7 Department of Neurological Surgery, Lou and Jean Malnati Brain Tumor Institute, Robert H. Lurie Comprehensive Cancer Center, Feinberg School of Medicine, Northwestern University, Chicago, IL USA

**Keywords:** Glioblastoma, 5ALA, Myeloid, Spatial transcriptomics, Mesenchymal subtype, Wound response, Glycolysis, Recurrence

## Abstract

**Background:**

Spatiotemporal heterogeneity originating from genomic and transcriptional variation was found to contribute to subtype switching in isocitrate dehydrogenase-1 wild-type glioblastoma (GBM) prior to and upon recurrence. Fluorescence-guided neurosurgical resection utilizing 5-aminolevulinic acid (5ALA) enables intraoperative visualization of infiltrative tumors outside the magnetic resonance imaging contrast-enhanced regions. The cell population and functional status of tumor responsible for enhancing 5ALA-metabolism to fluorescence-active PpIX remain elusive. The close spatial proximity of 5ALA-metabolizing (5ALA +) cells to residual disease remaining post-surgery renders 5ALA + biology an early a priori proxy of GBM recurrence, which is poorly understood.

**Methods:**

We performed spatially resolved bulk RNA profiling (SPRP) analysis of unsorted Core, Rim, Invasive margin tissue, and FACS-isolated 5ALA + /5ALA − cells from the invasive margin across *IDH*-wt GBM patients (*N* = 10) coupled with histological, radiographic, and two-photon excitation fluorescence microscopic analyses. Deconvolution of SPRP followed by functional analyses was performed using CIBERSORTx and UCell enrichment algorithms, respectively. We further investigated the spatial architecture of 5ALA + enriched regions by analyzing spatial transcriptomics from an independent *IDH*-wt GBM cohort (*N* = 16). Lastly, we performed survival analysis using Cox Proportinal-Hazards model on large GBM cohorts.

**Results:**

SPRP analysis integrated with single-cell and spatial transcriptomics uncovered that the GBM molecular subtype heterogeneity is likely to manifest regionally in a cell-type-specific manner. Infiltrative 5ALA + cell population(s) harboring transcriptionally concordant GBM and myeloid cells with mesenchymal subtype, -active wound response, and glycolytic metabolic signature, was shown to reside within the invasive margin spatially distinct from the tumor core. The spatial co-localization of the infiltrating MES GBM and myeloid cells within the 5ALA + region indicates PpIX fluorescence can effectively be utilized to resect the immune reactive zone beyond the tumor core. Finally, 5ALA + gene signatures were associated with poor survival and recurrence in GBM, signifying that the transition from primary to recurrent GBM is not discrete but rather a continuum whereby primary infiltrative 5ALA + remnant tumor cells more closely resemble the eventual recurrent GBM.

**Conclusions:**

Elucidating the unique molecular and cellular features of the 5ALA + population within tumor invasive margin opens up unique possibilities to develop more effective treatments to delay or block GBM recurrence, and warrants commencement of such treatments as early as possible post-surgical resection of the primary neoplasm.

**Supplementary Information:**

The online version contains supplementary material available at 10.1186/s13073-023-01207-1.

## Background

Isocitrate dehydrogenase-1 [*IDH1*]-wild-type glioblastoma (GBM) is a highly aggressive and heterogeneous tumor with poor survival outcomes. Despite radical multimodal treatment of aggressive surgery, radiation therapy, and chemotherapy with temozolomide, median survival has remained stagnant at 14.6 months from diagnosis [1]. A key contributing factor is the invasiveness of GBM deep into the neighboring brain parenchyma, which renders complete surgical resection impossible and efficacious brain penetration of chemotherapeutics a considerable challenge [2]. Furthermore, the ineffectiveness of therapeutic agents may arise from the plasticity of GBM cells which manifests intra- and inter-tumor heterogeneity; indeed, failed molecular targeted therapeutics have historically been focused on the GBM proliferative genotype predicated on tumor core alone [3]. Heterogeneity in GBM is well established and contributes to the differential expression of subclonal genes during the spatiotemporal evolutionary lifespan of the disease [4].

In search of the potential origins of phenotypic diversity and plasticity of GBM cells, emerging evidence indicates the existence of a rare GBM stem cell (GSC) subpopulation with self-renewing capacity [5, 6]. Major characteristics of the GSC include hijacking the normal neural stem cell developmental programs to promote and maintain tumor growth, and the acquisition of mechanisms to resist chemotherapy [7]. A recent single-cell RNA sequencing (scRNA-seq) study revealed high inter- and intra-GSC heterogeneity characterized by a transcriptional gradient composed of distinct gene signatures of two cellular states—“neural development” and “inflammatory wound response”—whereby a transcriptional program resembling a neural injury response in GSC may functionally contribute to GBM initiation [8]. To gain insight into the functional developmental and metabolic programs of GBM cells, Garofano et al. integrated scRNA-seq and bulk transcriptomics data by using a computational platform (single-cell biological pathway deconvolution (scBiPaD)) and showed that the distribution of GBM cells along neurodevelopmental and metabolic axes could facilitate their classification as “proliferative/progenitor”, “neuronal”, “mitochondrial” (MTC), and “glycolytic/plurimetabolic” (GMP) subtypes [9].

Based on transcriptional attributes, GBM was originally classified into four subtypes: Classical (CL), Neural (NE), Proneural (PN), and Mesenchymal (MES) subtypes [10]. However, a more recent study based on GBM transcriptomics, excluding non-malignant cell types, confirmed three subtypes of GBM—CL, PN, and MES [11], but where most patients exhibit intratumor plasticity of varying subtypes [4]. Moreover, longitudinal studies have demonstrated the temporal plasticity of GBM subtypes upon recurrence [11, 12]. Recently, Minata et al. showed that in response to the radiation-induced pro-inflammatory microenvironment, GBM cells at the tumor edge acquire an MES subtype defined by the expression of CD109 [13].

Tissue isolated from the invasive tumor margin region characterized by the MRI T2 high signal beyond the bulk tumor, where tumor blended into the brain in an invasive fashion (non-enhancing on T1 with gadolinium) (herein referred to as “Invasive margin”), harbors distinct genomic and transcriptomic profiles in contrast to tissue removed from the tumor core defined by the T1 with gadolinium non-enhancing or heterogeneously enhancing central tumor (herein referred to as “Core”) and enhancing rim (corresponding to peripheral strongly gadolinium-enhanced areas on T1 MRI, herein referred to as “Rim”) regions [14]. As recurrence of GBM is initiated within and beyond 2 cm of the Invasive margin post-surgery [15], unique molecular features that characterize this region may offer new therapeutically amenable targets to impair tumor regrowth. Nevertheless, the highly heterogeneous tissue cellularity of the Invasive margin, including infiltrated immune and healthy neural cells, poses a substantial challenge to filter the tumor-specific genomic and transcriptomic profiles from an overwhelming background of non-neoplastic cells.

A viable solution emerged from the use of 5-aminolevulinic acid (5ALA) during GBM neurosurgery [16]. 5ALA—a porphyrin—is metabolized to the fluorescent metabolite protoporphyrin IX (PpIX) by cells in which the heme biosynthetic pathway is activated, such as GBM cells, but not non-neoplastic cells [14]. The necrotic tumor core does not emit fluorescence owing to its deficiency of active heme metabolism; in contrast, Invasive margin fluoresces brightly and eventually fades with the decreasing number of tumor cells in the periphery [17]. We previously have shown that Invasive margin from freshly resected primary tissue harboring 5ALA + GBM cells can be purified from background non-neoplastic cells through fluorescence-activated cell sorting (FACS) [14]. We have demonstrated that transcriptomics analysis of spatially resolved tissues within this sampling framework can identify non-canonical molecular factors associated with GBM infiltration, such as *SERPINE1* [14]. However, deep transcriptomic profiling in concert with spatially resolved transcriptomics is required to fully elucidate the unique molecular signatures and spatial transcriptional heterogeneity of 5ALA + cells relative to Core. Furthermore, the prognostic potential of 5ALA + cells remains unresolved, which can be achieved by exploring the contribution of unique molecular features of 5ALA + cells to survival outcomes and GBM recurrence.

To address these questions, we performed spatially resolved bulk RNA profiling (SPRP) of unsorted Core, Rim, and Invasive margin tissue, in addition to FACS-isolated 5ALA + and 5ALA − cells across 10 GBM patients. We interrogated the SPRP-derived transcriptomic landscape to test the hypothesis that the 5ALA + subpopulation(s) is defined by unique transcriptional features. The deconvolution of SPRP followed by the integration of independent single-cell RNA-seq revealed unique cellular, metabolic, and transcriptional states. We further revealed the spatial architecture of the microenvironment harboring 5ALA + enriched regions by integrating spatially resolved transcriptomics (stRNA-seq). Lastly, we explored the association of unique transcriptional features of 5ALA + cells with GBM survival outcome and recurrence.

## Methods

### Patient enrollment and tissue sample collection

The GBM cohort used in this study was fully described in our previous study [14]. Briefly, Neuropathological and molecular analyses confirmed that all patients (*N* = 10) had de novo GBM with *IDH1*-wt and transcriptional regulator—*ATRX*-wt. Tissue samples were collected from the GBM patients at diagnosis (prior to Temozolomide and radiotherapy). The details of the status of each patient with corresponding clinical and genomic features are reported in Additional file 2: Table S1. GBM patients were operated on by a single surgeon at a major regional Neuroscience center (Nottingham, UK) with specimens collected after informed consent was obtained from the patients and under ethics committee approval (11/EM/0076). Tissue samples representing spatially distinct regions (Core, Rim, Invasive margin) of tumor samples from 10 GBM patients were retrieved (previously collected as part of an earlier study [14]). Briefly, 5ALA (20 mg/kg dose) was administered orally to patients 2–4 h prior to craniotomy and visualization of 5ALA-induced PpIX fluorescence. Aided by image guidance, multi-region tissue samples were collected from non-fluorescent or minimally fluorescent regions representing Core, while samples from the viable fluorescent region corresponded to Rim. The furthest region of 5ALA-induced PpIX fluorescence beyond the bulk tumor, where the tumor penetrated adjacent healthy parenchyma, corresponded to the Invasive margin. Histological diagnosis and formal postoperative diagnosis (including *IDH1* mutations, *ATRX* mutation, and *MGMT* methylation status) were included (Additional file 2: Table S1). Tissue samples from the enrolled GBM patients (*N* = 10) were subjected to bulk transcriptome measurements followed by subsequent Bioinformatics analyses. Out of 10 patients, two patients were subjected to histological analysis of hematoxylin and eosin-stained images. One additional IDH-wt GBM patient who received 5ALA + before surgery at diagnosis (prior to Temozolomide and radiotherapy) was enrolled and spatially distinct tissue samples were collected followed by radiographic and two-photon excitation fluorescence microscopic analyses.

### Tissue sample processing and FACS analysis

Cells were dissociated from the invasive margin and subjected to FACS based on 5ALA immunofluorescence as described by us previously [14]. Previously, we extensively investigated the 5ALA-induced PpIX fluorescence method to establish a robust pipeline to identify 5ALA + infiltrative GBM cells [14]. Briefly, during the processing of the primary cells from GBM patients, gating and separation of cells into 5ALA + and 5ALA − populations was performed using FACS via a two-stage process involving the enrichment of the 5ALA + population, followed by purification of the enriched population. U251 GBM cells incubated for 2 h with and without 5-ALA were used as controls to set gates for sorting. No significant cell viability differences were observed between 5ALA exposed and unexposed cells for any cell lines (all *t*-test *p*-values > 0.05). The cells were sorted using an excitation spectrum at 405 nm and an emission spectrum at 605–625 nm. The positive and negative sorted cells were subsequently centrifuged at 800 rpm (180 × *g*) for 5 min before being subjected to snap freezing.

### Hematoxylin and eosin staining and two-photon excitation fluorescence microscopy

Representative hematoxylin and eosin-stained image analysis of 5ALA + GBM infiltrative margin was conducted and scanned using NanoZoomer, magnification × 40, scale bar 100 μm. We analyzed the radiographic features of the Invasive margin (Inv) and investigated the distribution of 5ALA-induced PpIX fluorescence by the two-photon excitation fluorescence microscopy (TPEF) as described earlier [18]. Briefly, the segmentations of the GBM spatial regions such as enhancing tumor and non-enhancing tumor on T1-weighted pre- and post-contrast, T2-weighted, and T2-FLAIR sequences were generated by using DeepMedic [19] followed by the application of TPEF imaging [18] to determine the distribution of 5ALA-induced PpIX fluorescence.

### Immunohistochemistry

Tissues from spatially distinct regions of GBM were collected and immunohistochemistry was performed as described by us previously [2]. Samples were obtained from the Core (superficial and anterior medial), Rim (deep edge), and Invasive margin. Briefly, after the removal of paraffin wax, samples were treated with sodium citrate buffer (pH 6) for 40 min at 90 °C and washed with phosphate buffer solution (PBS) for 2 min. Then, 200 μL of peroxidase blocking solution was applied to cover the specimen for 5 min followed by washing with PBS. After the slides were dried, Ki-67 antibody (DAKO) and CD31 (DAKO) were applied at 1:50 dilution and incubated for 1 h at room temperature. Sections were washed with PBS before the addition of the secondary antibody (DAKO) and incubated at 37 °C for 30 min. Finally, substrate-chromogen solution (DAB) was applied to cover the specimen, incubated for 5 min, and rinsed gently with distilled water. An Olympus BX41 light microscope was used to visualize and capture the images of each GBM region.

Immunohistochemistry for NeuN was performed on four patient tissue microarrays (TMAs) containing three intratumor regions of nine patient tumors in triplicate. The histology of the tissues was confirmed as GBM by an experienced pathologist. Following deparaffinization through a xylene and alcohol series, antigens were retrieved via boiling sections in Tris–EDTA (pH 9.0). Once cooled, sections were washed in PBS buffer and then blocked using 20% normal goat serum. The sections were then incubated for 1 h at room temperature with rabbit monoclonal neuronal marker NeuN (Abcam, ab177487) at a dilution of 1:3000. Following three washes with PBS, the sections were incubated with secondary antibody (Dako Chemate EnVision kit) for 1 h at room temperature. Sections were then incubated with DAB-chromogen complex and incubated for 5 min. Counterstaining was performed with hematoxylin before rehydrating the sections by passing through the previous alcohol to xylene series. Sections were scored and neuron numbers counted, and statistical analysis via Student *t* test was performed using GraphPad Prism (9.0). Both Ki67 and NEUN staining was conducted on 4 tissue microarrays (TMAs) consisting of 9 patients per TMA with each region (Core, Rim, Invasive margin) in triplicate (*N* = 36 patients).

### RNA isolation and RNA-seq library preparation

Dissociation of Core, Rim, and Invasive margin was performed as previously described by us [20]. Total RNA extraction followed by quality control analysis was performed as described by us [14]. Briefly, libraries were prepared using the NEBNext Poly(A) mRNA Magnetic Isolation Module (NEB: E7490), the NEBNext Ultra Directional Library Kit for Illumina (NEB: E7420), and the NEBNext Multiplex Oligos for Illumina (Index Primers Set 1) (NEB: E7335L). Samples with a total RNA concentration of > 10 ng/µl (0.5 µg total amount) were used for library preparation. To ensure library quality, adequate concentrations were obtained from each sample, followed by 14 cycles of amplification during the PCR-based library enrichment step. Finished libraries were quantified using the Qubit dsDNA HS kit (Invitrogen: Q32854). Library concentrations, as well as fragment size distributions, were also analyzed by employing the Agilent Bioanalyzer High Sensitivity DNA Kit (Agilent: 5067–4626). Libraries were normalized to 2 nM and pooled in equimolar amounts. The Kapa Library Quantification Kit (KAPA Biosystems: KK4824) was used for the precise quantification of the library pool. The library pool was denatured and diluted to 1.6 pM, spiked with 1% PhiX (1.8 pM), and sequenced on the Illumina NextSeq 500, using the NextSeq 500/550 High Output v2 Kit (150 cycles) (Illumina: FC-404–2005), to generate a minimum of 70 million pairs of 75-bp paired-end reads per sample. Raw RNA-seq data have been deposited at ArrayExpress with accession number E-MTAB-8743.

### RNA-seq data processing

We obtained RNA-seq raw data (FASTQ files) from spatially distinct unsorted regions (Core, Rim, and Invasive margin) and 5ALA sorted cells from the Invasive margin across 10 GBM patients. RNA-seq raw data from spatially distinct regions were processed using Bioconductor package QuasR (version 1.30.0) [21]. For primary alignment, we used the reference genome hg19 for human. The QuasR package employs the required tools to obtain expression tables from the raw RNA-seq reads and includes the aligners Rhisat2 [22] and SpliceMap [23]. We performed the alignment by using the following command:

“qAlign (‘sampleFile.txt’, ‘BSgenome.Hsapiens.UCSC.hg19’, splicedAlignment = TRUE, aligner = ‘Rhisat2’)”.

We then measured the count of each gene within any annotated exonic region using the function qCount. The count data obtained from the QuasR package was then converted to transcripts per million (TMP) followed by log transformation (Log_2_) of TPM + 1 values. Initial quality control analysis was performed on the normalized RNA-seq counts including a comparison of differential expression across the samples. Genes with valid count values were additionally compared among the samples.

### Differential gene expression analysis

To identify differentially expressed genes, we used the linear modeling-based approach limma R/Bioconductor package [24] on transcriptome dataset. Briefly, we compared differential mRNA expression among unsorted tissues (Core, Rim, and Invasive margin) and sorted cells (5ALA + and 5ALA −). We selected significantly regulated genes with an adjusted *p*-value below 0.05 using the Benjamini–Hochberg correction method for multiple testing.

### Hallmark gene set enrichment analysis (GSEA)

Enrichment analyses of the hallmark gene sets representing biological processes related to cancer were carried out by a GSEA algorithm [25]. Briefly, the hallmark gene sets were selected from MSigDB gene-set collections [26], and enrichment analysis was conducted among the different regions (Core, Rim, and Invasive margin) and 5ALA sorted cells (5ALA + and 5ALA − cells) using GSEA. The ranked list of genes obtained from GSEA was further processed by Fast Gene Set Enrichment Analysis (fgsea) R-package [27]. Normalized enrichment score (NES), *p*-value, and adjusted *p*-values (calculated with a standard Benjamini-Hochberg—BH procedure) were retrieved for each of the hallmarks that were enriched in different regions and cell populations. The hallmarks with higher NES values and adjusted *p*-value < 0.05 were considered as enriched for a specific GBM region. For further analysis of the enriched pathways, the leading edge genes representing the subset of genes contributing significantly to the enrichment signal of a given gene set [25] in a specific GBM region, were identified and subjected to hierarchical clustering analysis. We employed a combined R Wrapper function ComplexHeatmap::pheatmap() that uses two R-packages, “pheatmap” and “ComplexHeatmap”, where the column and row clustering were performed by using the Euclidean distance method.

### Neural cell-type gene signature enrichment

To characterize the different GBM regions, transcriptome-based neural cell-type signatures described by Cahoy et al. were retrieved [28] (Additional file 2: Table S1). In brief, Cahoy et al. employed Affymetrix GeneChip Arrays to identify gene signatures of different neural cell types including neurons, oligodendrocytes, astrocytes, and cultured astroglial cells [28]. NES and adjusted *p*-values were calculated using GSEA and fgsea algorithms as described above.

### GBM subtype gene signature enrichment

Gene signatures of each GBM molecular subtype were obtained from Verhaak et al. describing an efficient gene expression-based molecular classification of GBM samples into four molecular subtypes: PN, NE, CL, and MES [10] (Additional file 2: Table S1). The signature gene set for each of the subtypes was retrieved from MSigDB. GSEA for the molecular subtypes was performed on the spatially distinct GBM regions. NES and adjusted *p*-values were calculated using the fgsea package as described previously.

### Developmental, inflammatory wound response, and metabolic gene signature enrichment

Signature genes for developmental and inflammatory wound healing/ injury response phenotypes were retrieved from Richards et al. (Additional file 2: Table S1). In addition, two gene sets representing two divergent metabolic phenotypes—mitochondrial (MTC), glycolytic/plurimetabolic (GPM)—were retrieved from Garofano et al. [9] (Additional file 2: Table S1). GSEA was performed to identify the enrichment of these diverse gene sets in spatially distinct GBM regions.

### Stemness gene signature enrichment

Diverse signature gene sets representing stem cells [29], cancer stemness [30], embryonic stem cells (ES1 and ES2) [31], human embryonic stem cells (hESC) [32], induced pluripotent stem cells (iPSC) [33], Nonog/Sox2 induced stem cell gene set [31], Myc induced ES gene set [34], and human epithelial adult stem cells [35] were retrieved (Additional file 2: Table S1). GSEA was performed to identify the enrichment of these diverse gene sets in spatially distinct GBM regions as described earlier.

### Deconvolution of GBM bulk RNA-seq data through CIBERSORTx

CIBERSORTx algorithm [36] was utilized to process spatially resolved bulk RNA-seq data from GBM 10 patients encompassing a bulk admixture of different cell types. First, signature matrix files representing the genes defining the expression profile for each cell type of interest were generated using previously published single-cell RNA-seq data. TPM-normalized scRNA-seq datasets were used for generating corresponding signature matrices. For this purpose, we used the scRNA-seq data representing six transcriptionally distinct GBM malignant cell types (AC-like, OPC-like, NPC1-like, NPC2-like, MES1-like, and MES2-like) in 28 *IDH*-wt GBM patients from Neftel et al. [37]. Secondly, scRNA-seq data, generated from 69,000 GBM stem cells (GSCs) cultured from the tumors of 26 patients, representing two distinct cellular transcriptomic states (Developmental and Inflammatory wound response) as reported by Richards et al. , was used to generate the signature matrix. For Richards et al., the GSCs were divided into three categories (High, Intermediate, and Low) based on the area under the curve (AUC) score [38] for Developmental (DEV) and Inflammatory wound response transcriptomic states. The corresponding signature matrices were then used to estimate the proportions of distinct cell types and transcriptional programs in bulk spatially resolved bulk RNA-seq data. Next, we used the “High-Resolution” expression analysis to impute sample-level gene expression variation in Mesenchymal, Inflammatory wound response, and 5ALA + signature genes of different cell types and transcriptional states across bulk RNA-seq profiles of 10 GBM patients.

### Single-cell wise gene signature scoring based on scRNA-seq data

UCell R-package [39] was utilized for determining the gene signature enrichment scores in the single-cell datasets. Briefly, based on the Mann–Whitney *U* statistic, UCell R-package allows to estimate single-cell wise signature scores called UCell scores for a given gene set by using the count matrix scRNA-seq dataset. In addition, to Neftel [37] and Richards et al. datasets, scRNA dataset from developing normal fetal brain as reported by Couturier et al. [40] was used. Briefly, the cells were isolated from the telencephalon of human fetuses (*N* = 4) of 13–21 gestational weeks. Microglia (CD45-positive) and endothelial cells (CD31-positive) were depleted by FACS sorting to enrich the CD133-positive cells (*N* = 10,093 cells) which were subjected to scRNA-seq. For scRNA-seq dataset from Neftel et al., gene signatures representing six distinct cell types (AC-like, OPC-like, NPC1-like, NPC2-like, MES1-like, and MES2-like) were used to estimate the UCell scores for each cell type. In addition, Neftel scRNA-seq data was re-analyzed to estimate the UCell scores by using four gene signatures (NEU, PPR, GPM, and MTC) as reported by Garfano et al. [9]. In the case of Richards et al. scRNA-seq data, gene signature from two transcriptional programs (Developmental and Inflammatory wound response). scRNA-seq dataset from Couturier et al. was employed to calculate the UCell scores for three gene signatures (DS1: Mixed population including truncated radial glial cells and cancer mesenchymal cells, DS2: Oligo-lineage cells/OLCs, and DS3: Glial progenitor cells/GPCs).

### Non-linear dimensional reduction by using tSNE followed by Louvain clustering

Seurat R-package [41] was used for the non-linear dimensional reduction of the scRNA-seq datasets (Neftel, Richards, and Couturier et al.). Briefly, we used the scRNA-seq count matrix to create a Seurat object followed up by the execution of the standard pre-processing workflow for scRNA-seq data in Seurat. These represent the selection and filtration of cells based on QC metrics, data normalization, and scaling, and the detection of highly variable features. After QC analyses, we normalized the data by employing a global-scaling normalization method “LogNormalize” that normalizes the feature expression measurements for each cell by the total expression. Then we utilized tSNE followed by the Louvain clustering algorithm to visualize and explore these datasets.

### Estimation of mRNA expression-based stemness index (mRNAsi)

Estimation of mRNA expression-based stemness index (mRNAsi) was performed by the method described by Malta et al. [42]. Briefly, to calculate mRNAsi, a machine learning approach was used to develop a predictive model by employing one-class logistic regression (OCLR) as described previously by Sokolov et al. [43]. The OCLR was based on the hESC and iPSC from the Progenitor Cell Biology Consortium (PCBC) dataset [44, 45]. The mRNAsi score ranges between 0 and 1, where 0 indicates less stemness with a more differentiated tissue state and 1 represents more stemness with a less differentiated state. To generate a stemness score based on the spatial RNA-seq data from 10 patients, and in comparison to The Cancer Genome Atlas (TCGA) GBM samples, a gene expression matrix (samples in columns and genes in rows) was prepared and employed on R-package “TCGAbiolinks” using the function TCGAanalyze_Stemness().

### Construction of the transcriptional network

Firstly, transcription factors in the leading gene sets representing inflammatory pathway, tumor necrosis factor-α (TNF-α) signaling via nuclear factor-кB (NFкB) pathway, MES subtype, inflammatory wound response, MTC, and GPM subtypes were manually curated using the ENCODE database [46]. We used the mutual information-based algorithm ARACNE [47] to construct the regulatory network between transcription factors and target genes based on mRNA expression values of the leading edge genes. The previously described bootstrap algorithm [48] was used to assess statistical confidence. We inferred 1000 networks based on bootstrap datasets, setting the most stringent value for Data Processing Inequality (DPI = 0) tolerance. Finally, we estimated the significance of the edges by testing their probability against a null distribution obtained by random permutation of predicted edges. The consensus network conserves edges with a *p*-value < 10^−4^. Based on the ARACNE output, we retrieved only the mutual information (MI) values for a given transcription factor and target genes for which the *p*-value was significant (< 0.05), and constructed and visualized the transcription factor—target gene network using Cytoscape [49].

### Exon–intron split analysis (EISA)

Exon–intron split analysis (EISA) was employed as described by Gaidatzis et al. [50] to investigate the changes in pre-mRNA (introns) and mature-mRNA (exons) counts across different regions of GBM, which leads to the quantification of transcriptional and post-transcriptional control of gene expression. R-package “eisaR” was used for the EISA. Briefly, after mapping the transcripts to a unique position in the genome, counts of annotated exonic reads representing mature mRNAs were quantified, in addition, to read counts that did not match any annotated exons (intronic). Normalization was performed for exons and introns separately by dividing each sample by the total number of reads and multiplying by the average library size. Based on these expression levels, only the genes with reasonable counts (average log_2_ expression level of at least 5) were selected for downstream analysis. Genes with overlapping reads were discarded due to difficulty assigning intronic reads to the respective genes. A differential exonic and intronic change among different regions was been performed with EdgeR, as described by Gaidatzis et al. where a *p*-value < 0.05 was considered significant. GSEA of Hallmark, 5ALA + cell-derived signatures and transcription factors were conducted as described before, with genes ranked based on the log_2_ difference of exon or intron normalized intensity between two regions used as input.

### qPCR validation

For qPCR validation, the expression profile of the *CD44* gene across spatially distinct GBM regions was used. Briefly, the gene expression profiling was performed using Human Stem Cell PCR Array (PAHS-405Z; RT2ProfilerPCR; Qiagen) to assess gene expression levels [2].

### Spatial transcriptomics (stRNA-seq) analyses

Tissue samples were collected from the pathologically diagnosed *IDH*-wt GBM patients (*N* = 16) after 5ALA-guided surgery, and subsequent spatial transcriptomics experiments were performed as part of a previous study described by Ravi et al. [51]. Briefly, the spatial transcriptomics measurements were conducted by utilizing the 10X Spatial transcriptomics kit (https://spatialtranscriptomics.com/). The downstream analysis of the spatial transcriptomics data was performed as described by Heiland et al. [52]. Briefly, the application of the st-pipeline (github.com/SpatialTranscriptomics-Research/st_pipeline) resulted in a gene count matrix and a spatial information file containing the x and y position and the H&E image. Seurat v3.0 package was used to normalize gene expression values. After the removal of the batch effects, data was scaled by a regression model. For spatial expression plots, we used SPATA 2.0 package [53]. Briefly, the scaled gene expression values to plot single genes or 5ALA + specific gene signature scores, using the 0.5 quantiles of a probability distribution fitting. The *x*-axis and *y*-axis coordinates are given by the input file based on the localization at the H&E staining. We computed a matrix based on the maximum and minimum extension of the spots used (32 × 33) containing the gene expression or combined scores. Spots without tissue covering were set to zero. The data are illustrated as surface plots by SPATA 2.0 package [53].

To minimize noise and technical artifacts, autoencoder network [54] based denoising approach was implemented. To infer copy number variation (CNV), we took advantage of the function InferCNV as implemented in SPATA 2.0. InferCNV was used to identify gains or loss of chromosomes. Surface plotting allowing the visualization of the gene expression of the barcode-spot’s with a spatial dimension was used where the x-esthetic and y-esthetic of the plot are mapped onto the respective coordinate variable. Distinct categorical features (5ALA + enrichment scores) signify a group of barcoded spots into experimental groups (5ALA-INV and 5ALA-CT). Differential expression analysis (DEA) was performed among the automatically generated or manually segmented experimental groups followed by the gene set enrichment analysis by implementing the hypeR package which uses hypergeometric testing for enriched gene sets in SPATA 2.0.

### Weighted spatial correlation

We performed spatially weighed correlation analysis of the preexisting established cluster signatures (from Neftel, Richards, and Garofano et al.) along with the 5ALA + gene signature individually for each sample by following the method described by Ravi et al. [51]. Briefly, a semi-parametric mixed geographic weighted regression model as implemented in GWmodel [55] was utilized. A non-adaptive approach under the assumption that the distances within spots are constant was used. The weighted matrix was then calculated by either a Gaussian or bi-square kernel distribution.

### Deconvolution of the bulk transcriptomics data from the TCGA-GBM cohort

To deconvolute the transcriptome data, bulk RNA-seq data from *IDH*1-wt primary (*N* = 154) and recurrent (*N* = 13) GBM tumors was retrieved from TCGA (https://tcga-data.nci.nih.gov/tcga/). For deconvolution, we used the scRNA-seq data representing six transcriptionally distinct GBM cellular states from Netfel et al. (AC-like, OPC-like, NPC1-like, NPC2-like, MES1-like, and MES2-like) [37], two distinct transcriptomic states from Richards et al. (Developmental and Inflammatory wound response) , and two metabolic states and two cellular states from Garofano et al. (GPM, MTC, NEU, and PPR) [9], to generate the signature matrix. CIBERSORTx algorithm [36] was utilized to process TCGA bulk RNA-seq data encompassing a bulk admixture of different cell types.

### Recurrent vs. primary GBM survival analysis

To compare the gene signature enrichments, in addition to TCGA *IDH*-wt GBM cohort, RNA-seq data was retrieved from Chinese Glioma Genome Atlas (CCGA) (*N* = 190; Primary = 109 and Recurrent = 81) (http://www.cgga.org.cn/) [56] and The Glioma Longitudinal AnalySiS (GLASS) (*N* = 60; primary = 30 and recurrent = 30) [57] cohorts. Gene signatures comprised representing TNF-α signaling, inflammatory response, MES subtype, inflammatory wound response, MTC subtype, and GPM subtype that were upregulated in 5ALA + cells, were used for GSEA as described earlier. In order to investigate the correlation between the unique 5ALA + gene signatures and the survival of GBM patients, a single-sample GSEA [58] was performed to identify the enrichment of the 5ALA + associated gene signatures for each primary and recurrent tumor sample from TCGA, CCGA, and GLASS cohorts. NES for each of the gene sets were accumulated to calculate the 5ALA + gene signature score for each primary and recurrent GBM patient. The Spearman correlation coefficient was calculated between the 5ALA + gene signature scores of GBM patients and survival data for primary and recurrent GBM separately.

We then performed Cox regression on the matched-recurrent (*N* = 30) and -primary (*N* = 30) samples from GLASS cohort. Apart from 5ALA + -specific gene signatures, we included confounding factors such as the Age and Gender of the patients. Firstly, a univariate Cox regression analysis including 5ALA + gene signature, Inf. wound response, MES, and Inf. Response was performed followed by multivariate regression analysis by using the survminer package.

## Results

### Differential regulation of cancer-specific and metabolic signatures in spatially distinct GBM regions

**Fig. 1:**
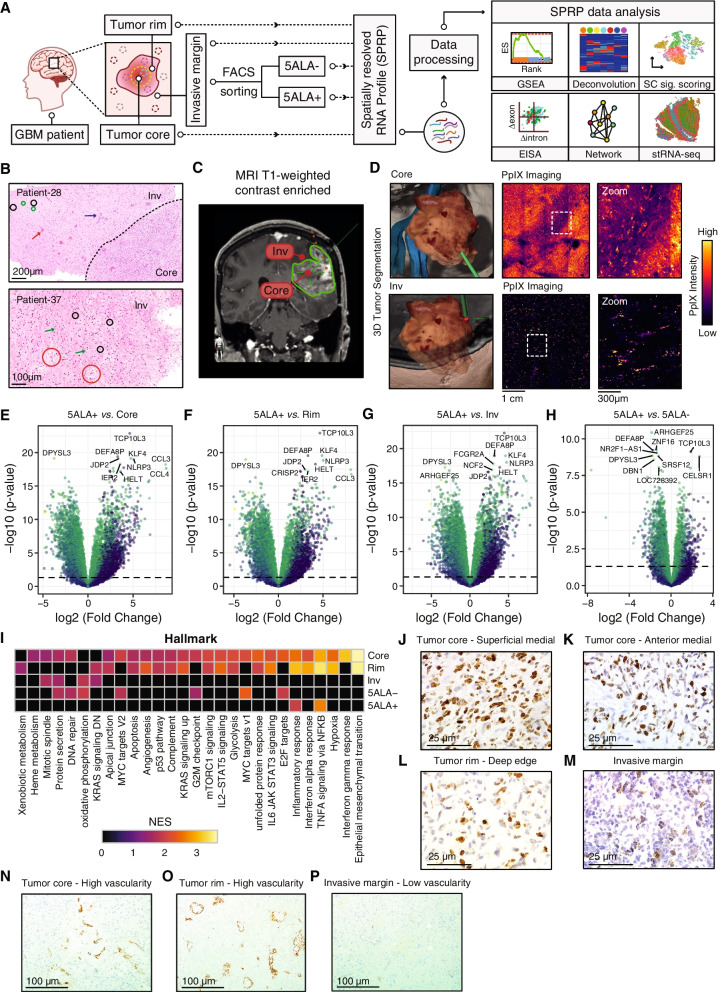
Differential gene expression analysis in spatially distinct GBM regions. Schematic figure delineating the steps for tissue collection from distinct GBM regions (Core, Rim, and Invasive margin) followed by 5ALA-based FACS isolation of Invasive margin cells into 5ALA + and 5ALA − subpopulations (**A**). The spatially resolved RNA profile (SPRP) from each unsorted region and sorted cells were interrogated by gene set enrichment analysis (GSEA), Deconvolution algorithm, single-cell gene signature scoring (SC sig-scoring), Exon–intron split analysis (EISA), network inference, and complemented by spatially resolved transcriptomics (stRNA-seq) (**A**). Representative hematoxylin and eosin-stained images of 5ALA + GBM infiltrative margin, scanned using NanoZoomer, magnification × 40, scale bar 200 μm for patient 28 and 100 μm for patient 37. Top (patient 28): Dashed line boundary demarcates cellular tumor-filling white matter. Individual tumor cells are observed within the cerebral neocortex (black circles), with neurons normally distributed (normally formed cerebral neocortex) (green circles). Microvascular proliferation (dark blue arrow) beyond the margin of the cellular tumor. Representative normal blood vessel (red arrow). Bottom (patient 37): Individual tumor cells are observed within the cerebral neocortex (black circles), with a normal distribution of white matter blood vessels (red circles). Deep white matter diffusely infiltrated by tumor cells at low density (green arrows). Other cells observed within this deep white matter region are oligodendrocytes with macrophages and astrocytes (**B**). The Inv margin representing the non-enhancing on T1 with gadolinium region located outside the MRI contrast region (**C**). The two-photon excitation fluorescence image demonstrates a distribution of PpIX across radiologically defined spatially distinct tumor core (Core) (**D**, Upper panel) and Invasive margin (Inv) (**D**, Lower panel) regions. Volcano plot representing differential gene expression between 5ALA + and Tumor Core (**E**), Rim (**F**), Invasive margin (**G**), and 5ALA − cells (**H**). Heatmap showing the normalized enrichment scores (NES) representing significantly enriched hallmarks (padj < 0.05) in a specific GBM region (**I**). GSEA was performed between a particular region and all other regions. The color represents the value of NES where yellow and black indicate the highest (NES = 3.5) and lowest (NES = 0) NES values, respectively. Only significant NES values are shown (adj. *p*-values < 0.05). Ki67 immunohistochemistry (IHC) of tumor core—superficial medial (**J**), anterior medial (**K**), tumor rim (**L**), and Invasive margin (**M**) to estimate the fraction of proliferating cells in spatially distinct regions of GBM. The scale bar indicates 25 µm. CD31-IHC represents the tumor vascularity identifying the number of vascular structures in the Core (**N**), Rim (**O**), and Invasive margin (**P**)

SPRP-derived RNA-seq data from tissue samples representing spatially distinct regions (Core, Rim, Invasive margin) across 10 GBM patients undergoing 5ALA-guided surgery were analyzed (Fig. 1A). Histological analysis of hematoxylin and eosin-stained images from representative patients (*N* = 2) revealed the cellular and anatomical features of the 5ALA + Invasive margin (Fig. 1B). For patient 28 (Fig. 1B, top), the dashed line boundary demarcates the boundary between the Core and Inv margin. Individual tumor cells are observed within the cerebral neocortex (black circles), with neurons normally distributed (normally formed cerebral neocortex) (green circles). Microvascular proliferation (blue arrow) beyond the margin of the cellular tumor, reflects trophic factors that are released by the tumor and permeate beyond the tumor margin. For patient 37 (Fig. 1B, bottom), individual tumor cells within the Invasive margin were observed within the cerebral neocortex (black circles), with a normal distribution of white matter blood vessels (red circles). Deep white matter diffusely infiltrated by tumor cells at low density (green arrows). In addition, oligodendrocytes, macrophages, and astrocytes were observed within this deep white matter region.

Moreover, results obtained by radiographic and two-photon excitation fluorescence microscopic analyses showed the spatially distinct nature of the Core and Invasive margin (Inv) from one additional GBM patient who received 5ALA + before surgery. The Inv margin represents the non-enhancing on T1 with gadolinium region located outside the MRI contrast region (Fig. 1C). The 5ALA-induced PpIX fluorescence was predominant in the Core (Fig. 1D, upper panel) compared to the Inv margin (Fig. 1D, lower panel). In the Inv margin, the intermittent PpIX fluorescence is visible within the background of normal brain cells (Fig. 1D, lower panel). These results demonstrated the spatially distinct nature of Inv from the previously reported enhancing region (ER) and enhanced margin (EM) [59].

Neuropathological and molecular analyses confirmed that all patients had GBM with *IDH1*-wt and transcriptional regulator—*ATRX*-wt (Additional file 2: Table S1). All samples were de novo *IDH*-wt GBM tumors, collected at diagnosis (prior to Temozolomide and radiotherapy). The details of the status of each patient with corresponding clinical and genomic features are reported in Additional file 2: Table S1. Cells from the Invasive margin were dissociated and subjected to FACS isolation based on 5ALA-induced PpIX fluorescence. Due to the distinct nature of the unsorted tissue and sorted cells (i.e., FACS process in the latter), we performed quality control analysis to compare the transcriptomes between unsorted tissues—Core, Rim, and Invasive margin—and sorted cells (5ALA + and 5ALA −). The Log_2_ normalized RNA-seq counts (TPM + 1) were uniform where inter-region variability did not supersede the intra-region patient variability in terms of gene expression in the unsorted regions (Additional file 1: Fig. S1A). However, variations were observed between the 5ALA-sorted cells and the unsorted tumor regions (Additional file 1: Fig. S1A). PCA results revealed consistent results and showed more transcriptome variability in the 5ALA-sorted cell populations (Additional file 1: Fig. S1B). The underlying cause of these differences could be attributable to both biological and technical factors. The percentage of 5ALA + cells of the total cell population in the Invasive margin samples gated based on 5ALA-induced PpIX fluorescence showed variation ranging from 0.90 to 2.90% with an average of 1.59% (Additional file 2: Table S1). The three patients (patients 30, 31, and 34) with lower 5ALA + transcriptome levels had a slightly lower average 5ALA + cell percentage (1.50%) compared to all other samples (1.63%). The lower read counts could possibly be due to the lower number of 5ALA + cells in these samples.

To evaluate gene expression comparability further, we calculated the Pearson correlation coefficients on the normalized mRNA expression data without housekeeping genes among the different regions and 5ALA sorted cells (Additional file 1: Fig. S1C). Correlation values across different tumor regions and cells were uniform, ranging from 0.88 to 1.00. When the expression levels of shared genes were compared between unsorted regions and sorted cells, a reasonable correlation was observed (Core *vs.* 5ALA − : 0.92, Rim *vs.* 5ALA − : 0.93, Invasive margin *vs.* 5ALA − : 0.91, Core *vs.* 5ALA + : 0.88, Rim *vs.* 5ALA + : 0.89, and Invasive margin *vs.* 5ALA + : 0.88) (Additional file 1: Fig. S1C). A higher correlation was also observed between 5ALA − and 5ALA + cells (*R* = 0.95). All *p*-values for the correlation coefficient are highly significant (*p*-value < 2.0 × 10^−16^). Overall, the highly correlated transcriptome of the unsorted tissue and sorted cells were indicative of their comparability in terms of reliable identification of gene signatures from the patient-specific background variability. We next aimed to identify molecular features of Invasive margin harboring the 5ALA + cells relative to Core, using comprehensive computational analyses (Fig. 1A). First, we employed DEA (differential expression analysis, Limma) to explore differentially regulated genes between 5ALA + cells and Core (Fig. 1E), Rim (Fig. 1F), Invasive margin (Fig. 1G), and 5ALA − cells (Fig. 1H), followed by pathway enrichment analysis where the upregulation of TNF-α signaling via NFкB and Inflammatory response pathways in 5ALA + cells was observed (Additional file 1: Fig. S2A and Additional file 2: Table S1). To determine cancer-related and metabolic pathways, a paired designed gene set enrichment analysis (GSEA) between a particular region/sorted cell and all other regions/sorted cell(s) was performed (Fig. 1I and Additional file 3: Table S2) where only the significant NES values (adj. *p*-values < 0.05) were considered as enriched. The Core exhibited the highest number of enriched pathways including epithelial-mesenchymal transition (EMT) and hypoxia (Fig. 1I and Additional file 3: Table S2). Akin to the Core, EMT, and hypoxia were highly enriched in the Rim (Fig. 1I and Additional file 3: Table S2). The Core was also enriched with pro-proliferative pathways such as mitotic-spindle, G2M checkpoint, mTOCR1 signaling, and E2F targets, whereas only mitotic-spindle was enriched in Inv (Fig. 1I). To investigate the distribution of proliferative cells across spatially distinct GBM regions, Ki-67 immunohistochemistry (IHC) was performed on tissue sections from GBM patients (*N* = 36), revealing a high number of proliferative cells in the Core superficial medial region (Fig. 1J) followed by anterior medial (Fig. 1K) and Rim regions (Fig. 1L). In contrast to the Core, the Invasive margin exhibited a lower number of proliferative cells (Fig. 1M and Additional file 1: Fig. S2B). The higher number of enriched pro-proliferative pathways in the Core was consistent with the increased number of proliferative cells observed in this region. The enrichment of the glycolytic pathway (Fig. 1I and Additional file 1: Fig. S2C, S2D) in addition to the absence of oxidative phosphorylation in the Core and Rim, was likely induced by the hypoxic conditions in these regions (Fig. 1I and Additional file 1: Fig. S2E and S2F). In contrast, hypoxia was neither enriched in the unsorted Invasive margin nor in 5ALA + and 5ALA-sorted cells. Enrichment of oxidative phosphorylation in Invasive margin and 5ALA − cells further corroborated the evidence suggesting the GBM infiltrative margin represents a normoxic microenvironment (Fig. 1I). CD31 IHC also reinforced this finding by showing that the Core and Rim were highly vascularized (Fig. 1N, O), whereas low vascularization was observed in Invasive margin (Fig. 1P). Interestingly, consistent with previous DEA results, TNF-α signaling via NFкB and Inflammatory response pathways were highly enriched in 5ALA + cells as well as in the Core and Rim (Fig. 1I, Additional file 1: Fig. S2A, S2G, and S2H and Additional file 3: Table S2).

To further investigate the gene expression in the enriched pathways, a hierarchical clustering algorithm was applied to the significantly regulated (Limma padj < 0.05) leading edge genes associated with glycolysis (Additional file 1: Fig. S2I), hypoxia (Additional file 1: Fig. S2J), and inflammatory response/TNF-α signaling via NFкB pathways (Additional file 1: Fig. S2K). Inter-tumor Core and Rim regions showed significantly higher relative expression of hypoxia response genes, including *LDHA*, *VEGFA*, *LOX*, *PLAUR*, *SERPINE1*, and *IGFBP3* (Additional file 1: Fig. S2J and S2L). Similarly, genes associated with glycolysis were significantly upregulated in the Core and Rim relative to the Invasive margin, 5ALA + , and 5ALA − cells (Additional file 1: Fig. S2I and S2M). In contrast, TNF-α signaling via NFкB, and Inflammatory response genes including *NFKB1*, *KLF6*, *IL6*, *CCL20*, *CCL2*, *CXCL3*, *SOCS3*, and *CXCL2*, exhibited significantly higher expression in 5ALA + and 5ALA − cells relative to all unsorted tumor regions (Additional file 1: Fig. S2K and Additional file 1: Fig. S2N).

Next, to identify the neural cell type that defines 5ALA + cells, we performed an enrichment analysis of gene signatures representing four neural cell types—oligodendrocytes, neurons, astrocytes, and cultured astroglia (Additional file 4: Table S3). Core and Rim were enriched with all four cell types representing a heterogeneous cell population, whereas Invasive margin and 5ALA − cells were mostly enriched with the neuronal cell type (Additional file 1: Fig. S2O). Intriguingly, no enriched neural cell type was identified for 5ALA + cells, indicating a likely evolution to a unique or mosaic cell-type signature that cannot be defined using canonical classifiers.

In summary, GSEA followed by hierarchical clustering revealed differential regulation of cancer-related and metabolic pathways in Invasive margin relative to Core, suggesting adaptation to different microenvironmental selection pressures.

### 5ALA + GBM population is enriched with a mesenchymal subtype, inflammatory wound response transcriptional program, and glycolytic metabolic state

**Fig. 2:**
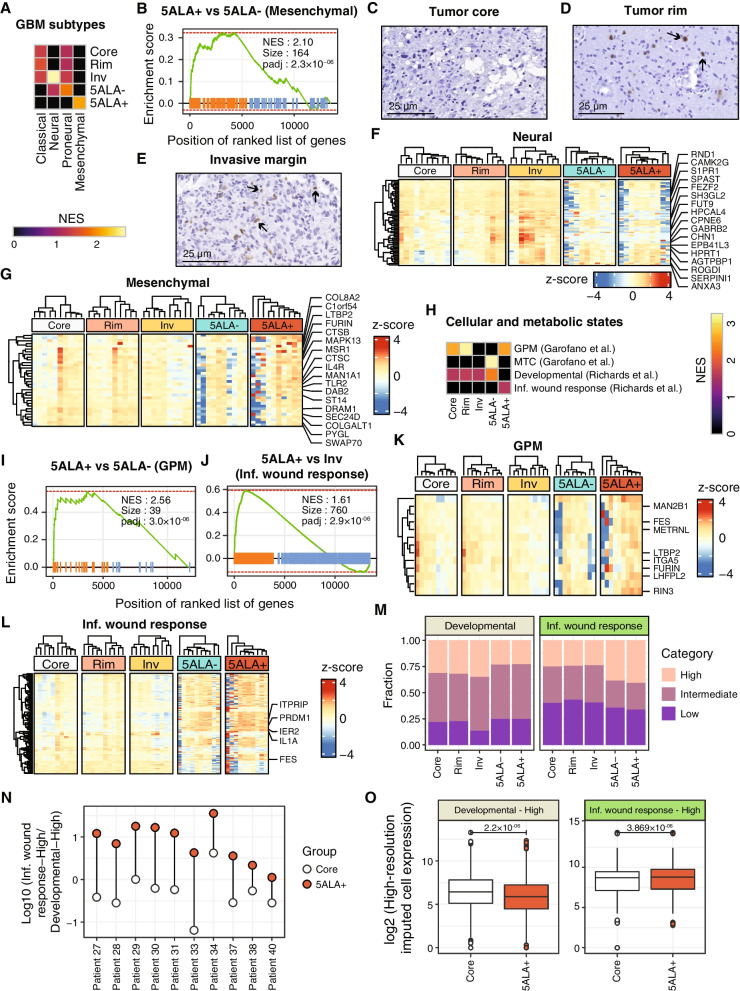
Enrichment of GBM subtypes, molecular and metabolic gene signatures in distinct GBM regions. The normalized enrichment scores (NES) of the significantly enriched (padj < 0.05) GBM subtypes are shown in distinct GBM regions and 5ALA sorted cells (**A**). Retrieval of gene sets specifying different GBM subtypes (Verhaak et al.) was followed by gene set enrichment analysis (GSEA). The color code indicates the differential NES values (yellow and black represent higher and lower NES, respectively). GSEA plot shows that the GBM mesenchymal subtype is significantly enriched (NES: 2.1, padj = 2.3 × 10^−6^) in 5ALA + cells (**B**). NeuN immunohistochemistry (IHC) of Core—(**C**), Rim (**D**), and Invasive margin (**E**) to estimate the proportion of NeuN positive (neuronal) cells (arrows). The scale bar indicates 25 µm. Differential *z*-scored normalized expression Log_2_(TPM + 1) of the significantly regulated (Limma, padj < 0.05) leading edge genes of GBM subtypes (Verhaak et al.)—Neural (F), and Mesenchymal (**G**)—are shown as a heatmap. Heatmap illustrating the normalized enrichment scores (NES) representing enriched cellular and metabolic states (padj < 0.05) in distinct GBM intratumor regions and 5ALA sorted cells (**H**). The gene signatures of cellular states (Developmental and Inflammatory wound response) and metabolic states (Glycolytic—GPM, and Mitochondrial—MTC) were retrieved and subjected to GSEA. GSEA plots represent the enrichment of GPM (**I**), and Inflammatory wound response (**J**) in 5ALA + cells compared to 5ALA − cells and Invasive margin, respectively. Heatmaps showing the differential expression of significantly regulated (Limma, padj < 0.05) leading edge genes of GPM (**K**) and Inflammatory wound response (**L**) in Core, Rim, Invasive margin, 5ALA − and 5ALA + cells. Stacked bar plot representing the transcriptional program estimates across 10 GBM samples (**M**). Each transcriptional program (Developmental and Inflammatory wound response) was divided into three expression-based categories based on their gene expression pattern (High, Intermediate, and Low). Each color indicates a specific expression-based category of a transcriptional program. The log_10_ ratios of High Inflammatory wound response and High Developmental transcriptional program in the unsorted Core region and 5ALA + cells across 10 GBM patients are shown (**N**). Box plots represent the median expression in Developmental and Inflammatory wound response genes enriched in cells with the high expression of different transcriptional programs (Developmental-High and Invasive margin-High) across Core and 5ALA + cell populations (**O**). *P*-values calculated from Student’s *t* tests are shown

To test the hypothesis that the 5ALA + cell population resembles a particular GBM subtype, we utilized enrichment analysis to identify GBM molecular subtypes (classical (CL), neural (NE), proneural (PN), and mesenchymal (MES) previously described by Verhaak et al. [10] (Additional file 5: Table S4). Core and Rim regions were mostly enriched with CL and PN subtypes compared to other regions (Fig. 2A) and in particular 5ALA + cells (Additional file 1: Fig. S3A and S3B), whereas the recently revised non-neoplastic NE subtype was highly enriched in the Invasive margin region (Fig. 2A and Additional file 1: Fig. S3C). 5ALA − cells were mostly associated with the NE and PN subtypes (Fig. 2A and Additional file 1: Fig. S3D); in contrast, 5ALA + cells were highly and uniquely enriched with the MES subtype in comparison to all other regions (Fig. 2A) and especially to 5ALA − cells (Fig. 2B).

To validate the cellular composition, IHC was performed on tissues from GBM patients (*N* = 36) to determine the expression of NeuN, representing the proportion of Invasive margin neuronal cells relative to Core and Rim. Core followed by Rim exhibited the lowest proportion of neuronal cells, compared to Invasive margin (Fig. 2C, D, and Additional file 1: Fig. S3E). The highest proportion of neurons was observed in Invasive margin, further corroborating spatial molecular signatures (Fig. 2E and Additional file 1: Fig. S3E).

Hierarchical clustering analysis of the significantly regulated (Limma padj < 0.05) leading edge genes showed that CL- and PN-specific genes were highly expressed in Core, Rim, and Invasive margin, relative to 5ALA + cells (Additional file 1: Fig. S3F, S3G, S3H, and S3J), whereas Invasive margin uniquely showed the highest expression of NE-specific genes (Fig. 2F, and Additional file 1: Fig. S3I). MES-specific genes exhibited a relatively higher expression in 5ALA + cells (Fig. 2G and Additional file 1: Fig. S3K). However, three patients showed relatively lower expression of MES genes while seven patients exhibited relatively higher MES expression. This result indicates that patient-specific MES gene expression variability exists in the 5ALA + population.

These results suggest that GBM molecular subtypes do not manifest uniformly throughout spatially distinct regions, but rather vary in a region-specific manner. The 5ALA + population localized within the invasive margin outside the MRI contrast-enhanced Core regions was enriched with the MES subtype.

Apart from molecular subtypes, Garofano et al. recently introduced two metabolism-associated GBM subtypes—mitochondrial (MTC) and glycolytic/plurimetabolic (GPM) [9]. Furthermore, it has been proposed that GBM transformation is initiated by a neural wound response transcriptional program activated in GBM stem cells (GSCs) . We, therefore, asked whether the transcriptional programs of GSCs and metabolic subtype(s) are active in 5ALA + cells, by processing gene signatures characterizing two GSC-derived transcriptional programs—Developmental and Inflammatory wound response (herein referred to as “Inf. wound response”) (Richards et al.) and two metabolic states—MTC and GPM (Garofano et al.) [9] by performing GSEA (Additional file 6: Table S5). The GPM genes (Additional file 2: Table S1) showed a minimal overlap (*N* = 2) with the classical glycolysis hallmark category (Additional file 3: Table S2). The GPM metabolic state has been defined by a diverse array of metabolic activities including glycolysis/hypoxia, lipids, amino acids, steroids, and iron/sulfur metabolism excluding mitochondrial/oxidative phosphorylation [9]. GSEA revealed differential enrichment of these transcriptional and metabolic states where Developmental and GPM states were enriched in Core and Rim, with only the Developmental program enriched in Invasive margin in comparison to all other regions (Fig. 2H). The MTC state was enriched in 5ALA − cells; however, unlike 5ALA − cells, GPM was enriched in 5ALA + cells compared to all other regions, indicating disparate metabolic states of 5ALA + and 5ALA − cells despite a shared infiltrative margin microenvironment and shared experimental processing via FACS (Fig. 2H). Interestingly, Inf. wound response was uniquely enriched in 5ALA + cells (Fig. 2H). For 5ALA + cells, the highest enrichment score was observed for GPM (Fig. 2I), followed by Inf. wound response (Fig. 2J) compared to 5ALA − cells and Inv margin, respectively.

Clustering analysis based on the significantly regulated (Limma padj < 0.05) leading edge genes (Additional file 6: Table S5) revealed a higher expression of GPM-associated genes in the 5ALA + cells for 7/10 patients (Fig. 2K and Additional file 1: Fig. S4A). Most of the genes associated with Inf. wound response were highly upregulated in the 5ALA + cells relative to other regions (5ALA + *vs.* Core: *p*-value = 0.0085 and 5ALA + *vs.* Invasive margin: *p*-value = 0.0006) (Fig. 2L, and Additional file 1: Fig. S4B).

Next, we performed deconvolution of the bulk SPRP data based on the Inf. wound response and Developmental gene signatures from Richards et al. [18] using CIBERSORTx. The single-cell wise area under the curve scores (AUCscores) for Developmental and Inf. wound response transcriptional programs were retrieved from Richards et al. [18] and categorized into three expression categories (High, Intermediate, and Low). Single cells were annotated into expression categories based on the AUCscores representing Developmental and Inf. wound response transcriptional programs. Signature matrices representing the High, Intermediate, and Low expression of Developmental (Additional file 1: Fig. S4C) and Inf. wound response (Additional file 1: Fig. S4D) were generated (Additional file 6: Table S5). The higher expression of the corresponding Developmental (e.g., *CCND2*) and Inf. wound response (e.g., *NFKBIA*) markers validated the signature matrices of the Developmental_High_ and Inf. wound response_High_ groups, respectively. By utilizing the signature matrices, the cells with different expression categories (High, Intermediate, and Low) were estimated across 10 GBM patients for Developmental (Additional file 1: Fig. S4E) and Inf. wound response (Additional file 1: Fig. S4F) transcriptional programs (Additional file 6: Table S5). The abundance of cells with the Developmental_High_ program was higher in unsorted regions compared to sorted cells (*p*-value = 0.001) (Fig. 2M). The fraction of Inf. wound response_High_ was significantly higher in 5ALA + cells compared to Core (adj. *p*-value = 0.048), Rim (adj. *p*-value = 0.045), and Inv (adj. *p*-value = 0.03) (Fig. 2M and Additional file 6: Table S5).

Interestingly, an increased fraction of cells harboring Inf. wound response_High_ transcriptional program was observed in sorted cells, with the highest abundance in 5ALA + cells. Particularly, the differential proportions of cells with Developmental_High_ and Inf. wound response_High_ programs between Core and 5ALA + cells were prominent (Additional file 1: Fig. S4G). To further explore, a Log_10_ ratio between cells with Inf. wound response_High_ and Developmental_High_ programs showed a positive value in the 5ALA + population across all 10 GBM patients, while the Core exhibited a near-zero or negative ratios (Fig. 2N), implying that the 5ALA + cell population is characterized by the activation of Inf. wound response program. For further validation, the expression of the Developmental and Inf. wound response genes (Additional file 6: Table S5) were compared between cells with Developmental_High_ and Inf. wound response_High_ transcriptional programs, respectively within the Core and 5ALA + cell population. Developmental program expression was significantly higher (*p* = 2.2 × 10^−6^) in the Core while Inf. wound response expression was significantly upregulated (*p* = 3.86 × 10^−6^) in the 5ALA + cell population (Fig. 2O).

In summary, these results highlighted the activation of differential transcriptional and metabolic states throughout spatially distinct GBM regions and 5ALA-sorted cells. The activation of the Inf. wound response program was unique to the 5ALA + subpopulation relative to Core. Conversely, 5ALA + cells are likely to retain their GPM state analogous to the Core.

### 5ALA + population represents transcriptionally concordant invasive malignant and myeloid cells with active mesenchymal subtype and Inflammatory wound response program

MES, Inf. wound response, GPM, and TNF-α signaling/inf. response signatures were enriched in all 5ALA + cell populations across 10 GBM patients except Patient 31 (Additional file 1: Fig. S5A). We additionally performed a Generally Applicable Gene-set Enrichment for Pathway Analysis (GAGE) between 5ALA + cells and unsorted regions (Core, Rim, and Inv) and 5ALA − cells. GAGE confirmed that the proposed gene signature (*N* = 251) is highly enriched in 5ALA + cells compared to intratumor regions and 5ALA − cells (Additional file 6: Table S5). By taking advantage of the different gene signatures that were enriched in 5ALA + cells, a combined gene set was prepared by taking the non-redundant genes (*N* = 251) termed as “5ALA + gene signature”.

To test whether the proposed 5ALA + genes exhibit different expression levels in 5ALA + and 5ALA − cells, we further investigated the differential gene expressions of 5ALA + and 5ALA − cells with respect to Core. In brief, we took the 5ALA + gene signature (*N* = 251) and plotted the log2 fold change (FC) of each gene between 5ALA + *vs.* Core and 5ALA − *vs.* Core. Although a high correlation was observed (*R* = 0.75), the values were skewed indicating a higher amplitude of regulation in 5ALA + cells (Additional file 1: Fig. S5B). We then performed a *t*-test between the FCs, and the result clearly indicated that genes expressed by 5ALA + cells were significantly upregulated compared to 5ALA − cells (Additional file 1: Fig. S5C). *P*-value (*p*-value = 0.001) is shown.

To investigate a plausible confounding factor of whether the 5ALA + gene signature includes genes directly associated with FACS-induced stress, we analyzed bulk RNA-seq data representing 5ALA-based FACS sorted cells and unsorted tissue from the Core, Rim, and Inv regions from one additional GBM patient. Firstly, we performed a Limma analysis to identify differentially expressed genes (DEGs) between sorted (5ALA + and 5ALA −) cells and unsorted tissue (Core, Rim, and Inv). Three hundred and seventy six upregulated and 284 downregulated DEGs were identified (adjusted *p*-value > 0.05). When we compared the 5ALA + gene signature (*N* = 251) with the DEGs, only 8 genes were found overlapping with the upregulated DEGs indicating a minimal impact of FACS sorting on the 5ALA + gene signature. The GSEA enrichment analysis showed the intersecting 8 genes (*IL10*, *CD83*, *CSF2*, *MAP3K8*, *GADD45B*, *KLF6*, *IL10RA*, and *MAFF*) as gene members of the IL2-STAT5 pathway (adjusted *p*-value = 1.33 × 10 − 17). Overall, this new analysis highlighted the minimum impact of the FACS process on the sorted cells.

To characterize the cellular states of the unsorted regions (Core, Rim, and Inv) and sorted cells (5ALA + and 5ALA −), we performed deconvolution of the bulk SPRP dataset using the CIBERSORTx algorithm to estimate cell-type abundances. Briefly, scRNA-seq data was processed by CIBERSORTx to build a signature matrix defining the genes that are specific for a particular cell state. The signature matrix was then utilized to estimate the cell-type proportion across distinct GBM regions and sorted cells (Additional file 1: Fig. S5D and Additional file 5: Table S4).

Surprisingly, we observed that a fraction of the myeloid cellular state (MG pro-inflammatory type II) was significantly higher in 5ALA + populations compared to all other intratumor regions (Core: adj. *p*-value = 0.02, Rim: adj. *p*-value = 0.02, Inv: adj. *p*-value = 0.02) (Additional file 1: Fig. S5D and Additional file 5: Table S4).

The presence of the myeloid signature in the 5ALA + population may signify a heterogenous cellular state in the 5ALA + population including myeloid cells. Nevertheless, the malignant nature of the 5ALA + population was shown in our previous study where we confirmed that the 5ALA + cells isolated from the resected Inv region by FACS, harbors tumor cells [1]. Briefly, multiple subcutaneous xenograft implants were performed using different regions from the resected primary tumor (*N* = 10). No tumor uptake was evident in any animal injected with 5ALA − cells after approximately 144 days, whereas animals (*N* = 2) injected with 5ALA + cells grew tumors requiring sacrifice at 138- and 145-day postimplant, respectively. In contrast, 5ALA − injections did not generate a subcutaneous tumor representing a minimal cubic volume.

**Fig. 3:**
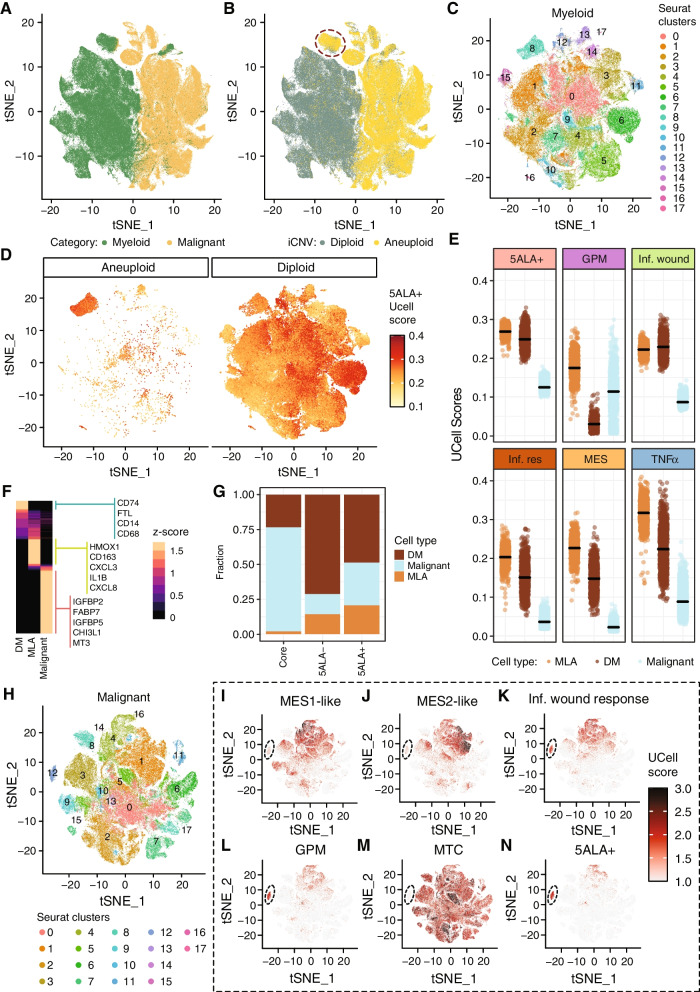
Enrichment of 5ALA + gene signatures in myeloid-like aneuploid, diploid myeloid, and non-myeloid malignant cells. tSNE plot representation of the sing cells (*N* = 261,092) annotated by GBmap as malignant and myeloid cell types (**A**). The color code represents the proposed cell annotation from GBmap. Copy number variation (CNV) categories (aneuploid and diploid) based on were mapped as different colors (aneuploid: Orange and diploid: Green) (**B**). tSNE plot with Louvain clustering of myeloid cells from GBmap dataset (*N* = 134,405, as annotated by Gbmap) (**C**). tSNE representation of the myeloid-like aneuploid (*N* = 5171) (left) and diploid myeloid (*N* = 127,483) (right) cells. The color code represents the 5ALA + UCell enrichment score calculated based on the expression of the 5ALA + -specific genes (*N* = 251) (**D**). UCell scores of different gene signatures (5ALA + , GPM, Inf. wound, Inf. response, MES, and TNF***α***) across aneuploid myeloid (AM), diploid myeloid (DM), and non-myeloid-like malignant (malignant) cells (**E**). CIBERSORTx-derived signature matrix based on three cell annotations (DM, MLA, and Malignant) (**F**). Color code represents the *z*-scored expression. Selected differentially expressed genes are shown. Estimated fractions of different cellular states (MLA, DM, and Malignant) across unsorted Core and sorted 5ALA + and 5ALA − cells (**G**). Louvain clustering based on the single-cell RNA-seq data from GBmap (**H**). The color code represents 18 different clusters (cluster 0 to cluster 11). tSNE plots of GBmapdataset are shown where the color code represents the single-cell wise UCell scores for different gene signatures—MES1-like (**I**), MES2-like (**J**), Inf. wound response (**K**), GPM (**L**), MTC (**M**), and 5ALA + (**N**)

Previously Gangoso et al. [60] showed that GBM cells acquire the MES signature module upon immune attack followed by alterations of the transcriptional landscape driven by an epigenetic mechanism that aids GBM cells to mimic the transcriptional landscape of myeloid cells including microglia. Therefore, we cannot rule out the possibility that the 5ALA + population may harbor malignant cells that can acquire myeloid-like transcriptional programs. This may reflect the capacity of 5ALA + malignant cells with the MES subtype mimicking myeloid transcriptional features. To test the hypothesis that malignant GBM cells can acquire a transcriptional state analogous to myeloid cells, we first analyzed the malignant and myeloid cells from GBmap by tSNE followed by Louvain clustering (Fig. 3A). Subsequently, we mapped the cell-specific CNV (representing Chr 7 gain and Chr 10 loss for each cell) categories onto the malignant and myeloid cells (Fig. 3B). Intriguingly, the CNV analysis identified a distinct cluster of aneuploid cells (marked by a black circle) that were annotated as myeloid cells based on transcriptional features only (Fig. 3B). The remaining myeloid cluster was predominantly diploid. From these results, it is evident that transcriptome-based cellular annotation strategies may not have sufficient resolving power to differentiate two cell populations with similar transcriptomic but distinct genomic features. To investigate this further, we performed tSNE with Louvain clustering on the aneuploid- and diploid myeloid cells (*N* = 132,654) resulting in 18 clusters (Fig. 3C). We performed Fisher’s exact test to observe the distribution of aneuploid and diploid cells across the clusters and to identify any overrepresentation of aneuploid cells in a particular cluster. Results revealed that aneuploid cells were highly enriched in Cluster 8 (odds ratio = 80.52, CI = 75.07–86.36, *p*-value < 0.001). We then assessed the enrichment of the 5ALA + gene signature by calculating the Ucell score and subsequently mapped 5ALA + UCell scores on two myeloid-like populations of cells: myeloid-like aneuploid (MLA) cells (*N* = 5171) and diploid myeloid (DM) (*N* = 127,483) cells (Fig. 3D). The 5ALA + gene signature was highly enriched in MLA cells (Cluster 8) which may represent the malignant cells that are transcriptionally analogous to myeloid cells. Further investigation revealed that DM cells (predominantly cells belonging to Cluster 6) are also enriched with the 5ALA + gene signature. Next, we investigated the enrichment of 5ALA + -specific gene signatures (5ALA + , GPM, Inf. wound, Inf. response, MES, and TNFα) in each of these cellular categories (MLA, DM, and non-myeloid-like malignant cells) by calculating the UCell scores (Fig. 3E). MLA cells were highly enriched with 5ALA + signature compared to DM and non-myeloid-like malignant cells (adj. *p*-value < 0.001) (Fig. 3E, Additional file 5: Table S4). In addition, MLA cells showed higher UCell scores for GPM, Inf. wound, MES, and TNFα gene signatures (Additional file 5: Table S4). For Inf. wound, both MLA and DM cells showed high UCell scores compared to non-myeloid-like malignant cells. High enrichment of 5ALA + and associated gene signatures in MLA cells indicate that this aneuploid population, although transcriptionally similar to myeloid cells, may contribute to the malignant potency of the 5ALA + cell population.

Based on this newly defined ploidy-based annotation (MLA, DM, and non-myeloid-like malignant cells), we established a signature matrix (Fig. 3F) from GBmap dataset and subsequently deconvolute the transcriptomes from spatially resolved Core, and 5ALA-based sorted samples (5ALA + and 5ALA −) by employing the CIBERSORTx algorithm. We excluded the Inv margin as the signature matrix includes only the malignant and myeloid cellular states, whereas the normal cellular states (Astrocytes, Oligodendrocytes, etc.) relevant to the Inv margin are excluded. However, we included the 5ALA − population in order to compare myeloid fractions with 5ALA + cells. Estimation of fractions by CIBERSORTx revealed that indeed the MLA fraction was higher in the 5ALA + population compared to the Core and 5ALA − population (Fig. 3G and Additional file 5: Table S4), thus rendering approximately 50% of the 5ALA + population malignant (non-myeloid-like malignant and MLA cells) whereas the remaining fraction may belong to DM cells. In contrast, the 5ALA − population predominantly consisted of a DM population (~ 75%). These new analyses hinted towards the possibility that 5ALA + cells may represent a heterogenous population encompassing transcriptionally similar aneuploid malignant cells and myeloid cells. However, the high enrichment of the 5ALA + -specific gene signatures in MLA cells renders this population the major contributor to the malignant nature of the 5ALA + population. This posits an interesting scenario whereby myeloid cells are either capable of uptaking and metabolizing 5ALA and/or uptaking the 5ALA-derived PpIX. This very notion has been shown by recent studies [18, 61, 62]. In conclusion, these results underscore the possibility of heterogeneous cellular states in the 5ALA + population predominantly consisting of transcriptionally concordant malignant and myeloid cells.

Next, we turned our attention to the malignant characteristics of the 5ALA + population.

Using the established 5ALA + gene signature, we next aimed to further explore GBM-associated cellular, metabolic, and transcriptional signatures of 5ALA + cells by analyzing the latest publicly available single-cell datasets. To achieve this, we investigated the enrichment of 5ALA + gene signature in single cells with distinct cellular states (OPC-like, NPC-like, AC-like, and MES-like) from Neftel et al. [37] and transcriptional programs (Developmental and Inf. wound response) from Richards et al. [63] (Additional file 2: Table S1). Single-cell wise UCell scores of 5ALA + gene signature were mapped onto a two-dimensional scatter plot of cellular states based on the meta-module score. The meta-module score is defined by the gene sets for which the expression varies between cells across the tumor samples [37]. Results showed that MES-like and AC-like cells are enriched with the 5ALA + gene signature, further confirming that the 5ALA + cell population predominantly harbors MES-like states (Additional file 1: Fig. S5E and Additional file 7: Table S6). To distinguish between MES-like and AC-like cells, we first defined the MES-like (setting the cut-off: + 1 on the *x*-axis and − 1 on the *y*-axis) and AC-like (− 1 on the *x*-axis and − 1 on the *y*-axis) cell (Additional file 1: Fig. S5E) states based on the meta-module scores. In total, 549 MES-like and 787 AC-like cells were selected. MES-like cells are significantly enriched with the 5ALA + gene signature compared to AC-like cells (Additional file 1: Fig. S5F, Wilcox test, *p*-value = 2.2 × 10^−10^).

Next, we mapped the single-cell wise UCell scores of the 5ALA + gene signature onto a PCA plot of cells with two transcriptional programs—Developmental and Inf. wound response—revealing that the Inf. wound response cell cluster is enriched with the 5ALA + gene signature (Additional file 1: Fig. S5G), thus corroborating the previous findings that the 5ALA + cell population harbors active Inf. wound response transcriptional programs. Furthermore, we selected the Developmental and Inf. wound response-positive cells based on the respective AUCscores as reported by Richards et al. [63]. Cells were sorted according to Developmental and Inf. wound response AUCscores and cells with the top AUCscore (*N* = 10,000) were retained. The 5ALA + UCell scores were then compared between Developmental (*N* = 10,000) and Inf. wound response (*N* = 10,000) positive cells. Inf. wound response cells exhibited a significantly higher 5ALA + score compared to Developmental cells (Wilcox test, *p*-value = 2.2 × 10^−16^) (Additional file 1: Fig. S5H).

To validate the association of a GPM metabolic state with MES-like and Inf. wound response, the GPM enrichment scores (UCell Score) were compared between MES-like and AC-like cells by a bootstrapping method (as previously discussed). Results revealed that MES-like cells are significantly enriched with the GPM gene signature, compared to AC-like cells (The mean *p*-value = 1.01 × 10^−05^, range 1.01 × 10^−16^–9.37 × 10^−04^) (Additional file 1: Fig. S5I and S5J). Similarly, a comparison of GPM UCell scores between Developmental (*N* = 10,000) and Inf. wound response (*N* = 10,000) positive cells showed that the GPM score was significantly higher in Inf. wound response cells (*p*-value = 2.2 × 10^−16^) (Additional file 1: Fig. S5K and S5L). These analyses showed that MES-like and Inf. wound response-positive cells were also enriched with the GPM metabolic state.

Garofano et al. [9] provided extensive metabolic validation of GBM cells enriched with glycolysis-dependent (GPM) and oxidative phosphorylation-dependent (MTC) gene signatures where a cohort of patient-derived cellular (PDC) models of GBM was utilized. By using a random forest machine learning classifier, the authors defined PDCs (based on bulk RNA-seq data) into either GPM (*N* = 21) or MTC (*N* = 26) subtypes to validate the metabolic states of these cells using multiple metabolic parameters, including extracellular acidification rate (ECAR). We took advantage of the transcriptomics dataset representing the pre-defined GPM and MTC PDCs and performed a GSEA of the 5ALA + gene signature. Results indicated that PDCs with GPM state were also significantly enriched with the 5ALA + signature (Additional file 1: Fig. S5M), suggesting that PDCs enriched with the 5ALA + gene signature exhibit glycolysis-dependent metabolic features characterized by a higher rate of ECAR and glucose uptake. Collectively, the results reveal that the 5ALA + gene signature is strongly enriched in MES-like cells with active Inf. wound response program and GPM metabolic state. Based on these results, we hypothesized that GBM single cells with a 5ALA + signature are concomitantly enriched with MES, Inf. wound response, and GPM metabolic states. To explore the possibility, we performed a correlation analysis of UCell scores between the 5ALA + gene signature and different cellular states from Neftel et al. [37] (AC-like, MES1-like, MES2-like, NPC1-like, NPC2-like, and OPC-like), transcriptional programs from Richards et al. [63] (Inf. Wound response and Developmental), and metabolic gene signatures from Garofano et al. [9] (GPM, MTC, NEU, and PPR) (Additional file 7: Table S6). The UCell-based correlation analysis revealed a significantly higher positive correlation between the 5ALA + gene signature and MES1-like (*r* = 0.52; *p*-value < 2.2 × 10^−16^) (Additional file 1: Fig. S6A), MES2-like (*r* = 0.50; *p*-value < 2.2 × 10^−16^) (Additional file 1: Fig. S6B), Inf. wound response (*r* = 0.686; *p*-value < 2.2 × 10^−16^) (Additional file 1: Fig. S6C), and GPM (*r* = 0.405; *p*-value < 2.2 × 10^−16^) (Additional file 1: Fig. S6D). In contrast, UCell scores for OPC-like (Additional file 1: Fig. S6E) and MTC (Additional file 1: Fig. S6F) showed a negative correlation with the 5ALA + gene signature. Furthermore, a weak positive correlation (*r* = 0.11) was observed between the 5ALA + signature and AC (Additional file 1: Fig. S6G) whereas the 5ALA + gene signature was negatively correlated with NPC1 (Additional file 1: Fig. S6H), NPC2 (Additional file 1: Fig. S6I), Developmental (Additional file 1: Fig. S6J), PPR (Additional file 1: Fig. S6K), and NEU (Additional file 1: Fig. S6L).

To further uncover the malignant nature of the 5ALA + population, we first performed tSNE analysis based on the neoplastic cells from the GBmap dataset (Additional file 2: Table S1) followed by Louvain clustering and identified 18 clusters designated as cluster 0 to 17 (Fig. 3H). We subsequently mapped the UCell scores of MES1-like (Fig. 3I), MES2-like (Fig. 3J), Inf. wound response (Fig. 3K), GPM (Fig. 3L), MTC (Fig. 3M), and 5ALA + gene signature (Fig. 3N). Clustering analysis revealed a small cluster of rare 5ALA + cells (Cluster-12) overlapping with Inf. wound response and GPM clusters, but exhibited a clear distinction from clusters representing metabolic states (MTC). In addition, a diffuse distribution of the 5ALA gene signature was also observed in cluster 4. To quantify the overrepresentation of the 5ALA + signature in distinct clusters, we plotted the average 5ALA + UCell score per cluster (Additional file 1: Fig. S6M) and performed a Wilcox test. The average 5ALA + UCell score was significantly higher in cluster-12 followed by cluster-4 (Wilcox, *p*-value < 2.2 × 10^−16^), compared to all other clusters (Additional file 1: Fig. S6M). To identify the cellular state of the 5ALA + enriched clusters, we mapped the cell annotation on the tSNE plot (Additional file 1: Fig. S6N). Cluster-4 was predominantly MES-like while a mixed annotation (OPC-like, AC-like, and MES-like) was observed for cluster-12.

Collectively, this data substantiates MES cellular states, active Inf. wound response transcriptional program, and GPM metabolic state, as predominant in 5ALA + cells. Furthermore, 5ALA + cells may retain a hypoxia-dependent GPM state within discreet foci despite a normoxic Invasive margin microenvironment where the MTC metabolic state is predominant.

### Identification of transcriptional and post-transcriptional control in 5ALA + cells

Concomitant activation of multiple transcriptional programs in 5ALA + cells is likely to be controlled by shared transcriptional circuits governed by specific transcription factors (TFs). To address this, we constructed a transcriptional network controlling differentially regulated 5ALA + enriched genes associated with Inflammatory response, TNF-α signaling, MES, MTC, GPM, and Inf. wound response. Upon identification of the TFs in the 5ALA + enriched gene sets (*KLF4, NFкB1, NFKBIA, EGR2, REL*, and *FOSL2*), the TFs along with the 5ALA + enriched genes, were used as hubs and target genes respectively in the ARACNE algorithm, to identify the TF-target gene association significance (*p*-value < 0.05). Subsequently, a TF-target gene network was constructed (Additional file 8: Table S7) and visualized by the Cytoscape tool. All TFs except *FOSL2* showed a positive log_2_ fold change (FC) in 5ALA + compared to Core (Additional file 1: Fig. S7A) and 5ALA − cells (Additional file 1: Fig. S7B, Additional file 8: Table S7), and most target genes (nodes) were upregulated in 5ALA + cells (Additional file 1: Fig. S7A and S7B).

*REL* showed the highest number (*N* = 108) of interaction (edges) with nodes, closely followed by *KLF4* (*N* = 106) and *NFкB 1* (*N* = 98). The lowest number of target gene interactions was identified for *FOSL2* (*N* = 13), while all other TFs exhibited higher (> 70) target gene interactions (Additional file 8: Table S7). The TFs controlling the highest number of Inf. wound response genes were *KLF4* (*N* = 67), NFкB1 (*N* = 65), and *REL* (*N* = 52). The highest number of MES signature genes were likely to be controlled by *REL* (*N* = 16), and *NFкB1* (*N* = 14) (Additional file 1: Fig. S7C).

Overall, these results uncovered the shared transcriptional network associated with enriched cellular and metabolic states in 5ALA + cells, whereby the Inf. wound response and MES subtype are primarily controlled by *NFкB* and *REL*.

Exon–intron split analysis (EISA) was performed to determine the changes in pre-mRNA (intron) and mature-mRNA (exon) counts across distinct GBM regions and 5ALA-sorted cells. Compared to Core, Rim, and Invasive margin, a higher number of genes with significant intronic changes (∆intron) was observed in 5ALA + cells, than genes with significant changes in exon counts (∆exon) (Additional file 1: Fig. S8A-C) indicating a relatively strong transcriptional control regulating the genomic landscape of 5ALA + cells. In contrast, a lower number of genes with significant ∆exon (*N* = 140) and ∆intron (*N* = 93) counts were identified between 5ALA + *vs.* 5ALA − cells (Additional file 1: Fig. S8D) underscoring a similar global transcriptomic landscape of these cells, which is likely to be induced by a shared infiltrative margin microenvironment.

Furthermore, to investigate the transcriptional and post-transcriptional control of 5ALA + cell-specific cellular/metabolic gene signatures, we performed pre-ranked GSEA (Additional file 9: Table S8). The results showed significant enrichment of the MES subtype, Inflammatory response, and TNF-α signaling pathways, and GPM genes with upregulated ∆exon counts in 5ALA + cells (Additional file 1: Fig. S8E) relative to 5ALA − cells. Interestingly, a comparison of 5ALA + cells with unsorted regions (Core, Rim, and Invasive margin) mostly resulted in the enrichment of Inf. wound response, Inflammatory response, TNF-α signaling, and MES subtype, with increased ∆intron counts (Additional file 1: Fig. S8E) signifying transcriptional regulation controlling these genes.

Next, we identified significant DEGs with higher exon and intron counts in 5ALA + cells (Additional file 1: Fig. S8F-W). The highest number of DEGs were identified for Inf. wound response (*N* = 11) (Additional file 1: Fig. S8F, S8J-S, S8U). Only one DEG (*SIGLEC9*) was identified for the MES subtype (Additional file 1: Fig. S8H, S8W). For Inflammatory wound response, six genes (*IER2, PLS1, MOV10L1, CCL2, MMP25,* and *ADAMTSL5*) exhibited increased ∆exon counts and five genes (*MMP19, DMD, PLCXD3, MGLL,* and *BTBD11*) showed increased ∆intron counts in 5ALA + cells (Additional file 1: Fig. S8F, S8J-S, S8U). *CCL2* was associated with multiple gene signatures—(Inflammatory wound response, Inflammatory response, and TNF-α signaling) and showed significantly higher (*p*-value < 0.001) ∆exon counts in 5ALA + cells (Additional file 1: Fig. S8H, S8M).

Collectively, these results decipher the transcriptional and post-transcriptional regulation of enriched cellular and metabolic gene signatures in the 5ALA + cell population.

### 5ALA + cell population resembles CD44 expressing cancer mesenchymal cells

**Fig. 4:**
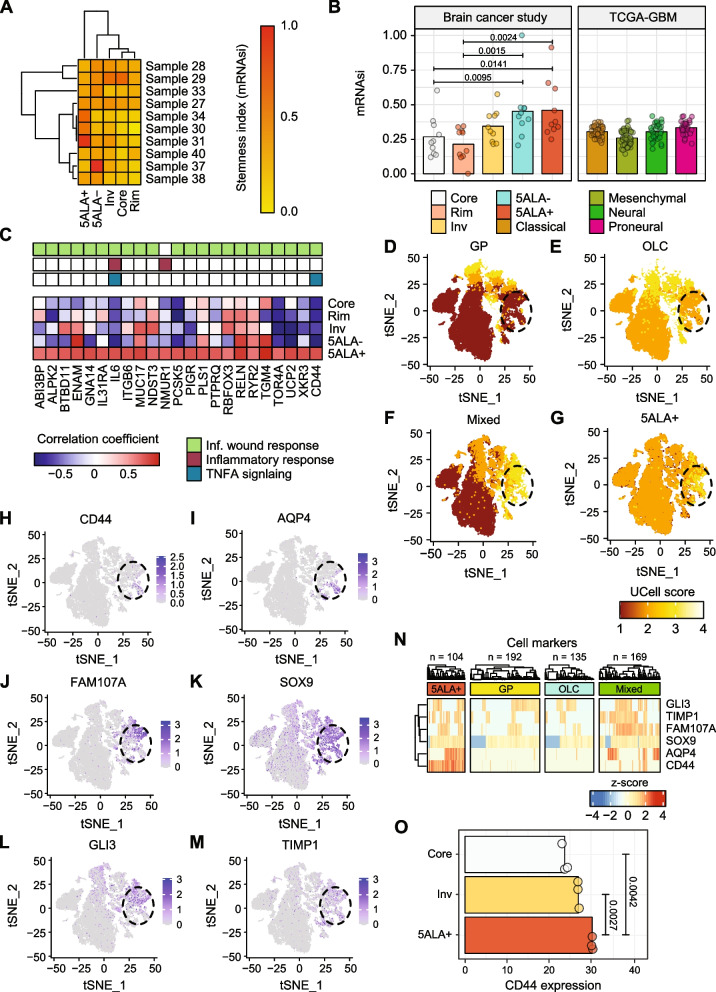
5ALA + cells represent *CD44* expressing mesenchymal cells. mRNA-based stemness index (mRNAsi) values across distinct GBM regions (Core, Rim, Invasive margin, 5ALA − and 5ALA +) for each patient are represented in a heatmap (**A**). Row (samples) and columns (GBM regions) are clustered by using a correlation algorithm. Comparison of the mRNAsi values according to brain regions (left) and TCGA-GBM samples (right) are shown as bar diagrams (**B**). The TCGA-GBM samples were pre-stratified according to GBM subtypes as described by Verhaak et al. [10]. Kruskal–Wallis tests showed a significantly higher mRNAsi in 5ALA + cells compared to Core and Rim, with *p*-values shown. Pearson correlation coefficient values between the mRNAsi and mRNA expression of selected genes are shown (**C**). The genes that showed a significant (*p*-value < 0.05) positive correlation with mRNAsi in 5ALA + cells for each patient were selected. tSNE clustering plots based on scRNA-seq dataset (Couturier et al.) are shown (**D–G**). The color code represents the single-cell wise UCell scores for different cell-signatures—glial progenitor cells (GP) (**D**), oligo-lineage cells (OLC) (**E**), mixed population including truncated radial glial cells, and cancer mesenchymal cells (**F**), and 5ALA + cells (**G**). tSNE clustering plots (Couturier et al.) with the color code representing the single-cell wise gene expression values for different marker genes—*CD44* (**H**), *AQP4* (**I**), *FAM107A* (**J**), and *SOX9* (**K**), *GLI3* (**L**), and *TIMP1* (**M**) are shown. Heatmap showing the *z*-scored log2 TPM expression of selected marker genes (*GIL3*, *TIMP1*, *FAM107A*, *SOX9*, *AQP4*, and *CD44*) based on scRNA-seq dataset (Couturier et al.) across different cell types (5ALA + , GP, OLC, and mixed population) (**N**). qPCR validation results showing Log_2_ gene relative gene expression of *CD44* gene in spatially resolved RNA profiles across Core, Invasive margin, and 5ALA + cells (**O**). *P*-values are shown as calculated by paired *T*-test

The activation of a GSC-derived Inf. wound response program in 5ALA + cells raises the possibility of retention of a stem-cell-like transcriptomic landscape. Thus, we aimed to interrogate the stem cell transcriptomic signature in 5ALA + cells by estimating a patient-specific stemness index (mRNAsi) [42] based on bulk mRNA expression (SPRP) data across 5ALA + , 5ALA − cells, and spatially distinct GBM regions. With the highest stemness index, 5ALA + cells showed a distinct profile relative to all unsorted tumor regions (Fig. 4A, B) where 5ALA + cells exhibited a significantly higher stemness profile compared to Core and Rim regions (Fig. 4B). Interestingly, stratification of TCGA-GBM samples (representing tumor Core) into molecular subtypes revealed a lower mRNAsi of GBM subtypes compared to both 5ALA + and 5ALA − cells (Fig. 4B). We took advantage of mRNAsi data from TCGA adjacent non-tumor tissue (NAT) representing 5ALA − cells, and compared this with TCGA-GBM tissue representing the tumor core. Bootstrap followed by the Wilcox test revealed that the NAT region has significantly higher mRNAsi values (median *p*-value = 0.015) compared to the GBM core, thus corroborating our results that 5ALA − cells showed higher mRNAsi values in comparison to Core (Additional file 1: Fig. S9A). Previously, an astrocyte precursor cell (APC) population with high proliferative capacity has been reported in GBM [8] which may explain the higher stemness of the 5ALA − cells and NAT region of GBM.

We retrieved the stemness-associated genes (*KLF4, MYC, CTNNB1, EPAS1, EZH2, KDM5B, NES, TWIST1, ABCG2, CD34, CD44, NANOG, PROM1, ZFP42*, and *ZSCAN4*) as reported by Malta et al. [42] and analyzed the correlation between mRNAsi and expression of these genes across the 10 patients (Additional file 1: Fig. S9B). Only two genes—*CD44* and *ZSCAN4*—showed a positive correlation between mRNA expression and mRNAsi (Additional file 1: Fig. S9B). Next, we obtained 13 known brain cancer and 13 stem cell markers from the cell marker database (http://biocc.hrbmu.edu.cn/CellMarker/help.jsp) [64] and investigated the correlation between mRNAsi and mRNA expression. Three genes—*ITGA6*, *SLITRK6,* and *SOX9*—showed a positive correlation (Additional file 1: Fig. S9C); however, when the expression levels of these genes were analyzed, *ZSCAN4, SLITRK6,* and *SOX9* showed low expression in 5ALA + cells (Additional file 1: Fig. S9D and S9E).

To further characterize the stemness-associated gene signature, eight previously published stemness-associated gene sets representing *Consensus Stemness* [33], *Human embryonic stem cell*—HuESC [32], *Stem cell* [29], *Myc induced genes* [34], *Embryonic stem cell—ES1 Sox2 induced genes* [31], *NANOG induced genes* [31], and *Epithelial Atypical squamous cells (ASC)* [35] were retrieved. GSEA showed that most of the gene sets were highly enriched in the Core, followed by Rim and Invasive margin regions, whereas 5ALA − cells were enriched in Consensus Stemness, HuESC, and Myc gene sets. Interestingly, none of these gene sets were enriched in 5ALA + cells (Additional file 1: Fig. S9F and Additional file 10: Table S9). Moreover, *Sox2*, an important gene for the stemness phenotype showed a very low expression across the 5ALA + populations. However, the stemness gene sets were predicated on classical stem cell niches, whereas the role of stem cell biology associated with the phenotype of infiltration is poorly understood. Therefore, the distinct mRNAsi index identified in the 5ALA + population may offer an avenue to elucidate whether stem cell plasticity may be associated with GBM infiltration.

Realizing the uniqueness of the 5ALA + cells, we performed a correlation analysis between stemness and 5ALA + signature genes. Only 23 genes showed a significant (*p*-value < 0.05) positive correlation with mRNAsi whereas all other genes (*N* = 228) showed predominantly negative correlations. The highest correlation was observed for the gene—Transglutaminase 4 (*TGM4*; R = 0.95). Although not significant (*p*-value = 0.08), *CD44*—a previously reported marker for Inf. wound response—showed a strong positive correlation (*R* = 0.57) with mRNAsi (Fig. 4C and Additional file 11: Table S10). This result indicates that although the 5ALA + population showed a relatively higher Stemness index, the lack of significant correlations between stemness-associated genes and 5ALA + gene signature renders the stemness character of 5ALA + cells unresolved.

To characterize the 5ALA + cells in terms of neurodevelopmental hierarchy, we utilized a scRNA dataset from normal fetal brain as reported by Couturier et al. [40] where cells were isolated from the telencephalon of human fetuses (*N* = 4) at 13–21 gestational weeks. Microglia (CD45-positive) and endothelial cells (CD31-positive) were depleted by FACS sorting to enrich the CD133-positive cells (*N* = 10,093 cells), which were subjected to scRNA-seq prior to characterization of glial progenitor cells (GP), oligo-lineage cells (OLC), and a mixed population including truncated radial glial cells and cancer mesenchymal cells. Single-cell wise UCell score of the 5ALA + gene signature was weakly correlated with GP (*r* = 0.040; *p*-value = 1.385 × 10^−09^), negatively correlated with OLC (*r* =  − 0.049; *p*-value = 9.959 × 10^−14^) (Additional file 1: Fig. S9G and S9H), but positively correlated with mixed population (*r* = 0.253; *p*-value < 2.2 × 10^−16^) (Additional file 1: Fig. S9I).tSNE plots based on the Couturier et al. scRNA-seq dataset followed by the mapping of single-cell wise UCell scores for different cell states—GP (Fig. 4D), OLC (Fig. 4E), mixed population including truncated radial glial cells, and mixed population (cancer mesenchymal cells) (Fig. 4F), and 5ALA + cells (Fig. 4G) were generated. tSNE analysis revealed that cells with an enriched 5ALA + gene signature localized distinctly (Fig. 4G) from GP and OLC but showed a significant overlap with the mixed population including truncated radial glial cells and cancer mesenchymal cells (Fig. 4F). To distinguish between these two populations, the expression levels of positive marker genes for truncated radial glial (*AQP4*, *FAM107A*, *SOX9*, and *GLI3*) and cancer mesenchymal (*CD44* and *TIMP1*) were investigated. Cells expressing *CD44* (Fig. 4H), *AQP4* (Fig. 4I), *FAM107A* (Fig. 4J), *SOX9* (Fig. 4K), *GLI3* (Fig. 4L), and *TIMP1* (Fig. 4M) overlapped with the 5ALA + cell cluster. To gain a better understanding, expression levels of truncated radial glial and cancer mesenchymal maker genes were compared across the cells with the highest enrichment of 5ALA + , GP, OLC, and mixed signatures (based on UCell scores) (Fig. 4N). Interestingly, the analysis revealed that cells (*N* = 104) with a high 5ALA + signature also expressed a high level of the cancer mesenchymal marker *CD44*, while almost half the *CD44* expressing cells (*N* = 64) also expressed the truncated radial glial marker *AQP4*. We further validated *CD44* gene expression across patient-matched spatially distinct GBM regions and 5ALA + cells, revealing high expression in 5ALA + cells relative to Core and Invasive margin (Fig. 4O). The experiment was performed on 5ALA + , Core, and Inv margin as the difference between 5ALA + and tumor regions (Core/Inv) would be most informative.

These findings provide evidence that 5ALA + may resemble cancer mesenchymal rather than truncated radial glial cells, yet suggest cellular heterogeneity within the 5ALA + cell population.

### Spatially resolved transcriptomics reveals the microenvironment harboring rare infiltrative 5ALA + cells

Having uncovered unique defining transcriptional features an important question regarding whether the 5ALA + cell population is confined to a particular spatial location or localized in multiple spatially distinct regions, remained elusive. Moreover, the localization of the 5ALA + population within the Inv margin has been determined by MRI and defined by 5ALA-induced PpIX fluorescence detected furthest into the area of MRI T2 high signal localized distantly from the Core (Fig. 1B–D), which is ethically safe to collect surgical biopsies from, and where the 5ALA + signal fades into the background of non-neoplastic cells and GBM penetrates into the brain in an invasive fashion (non-enhancing on T1 with gadolinium). The region that we are defining as “Invasive margin” is therefore spatially distinct from the enhancing region (ER) and enhanced margin (EM) as reported previously by Jin et al. [59]. To further gain insight, we investigated the distribution of 5ALA + signature across the spatially distinct anatomical regions characterized in the IVY glioblastoma dataset [65]. 5ALA + gene signature was predominantly expressed in hyperplastic blood vessels (HBV) and microvascular proliferation (MVP) regions but also diffusely distributed in other anatomic regions including pseudopalisading cells (PAN) and perinectoritc zone (PNZ) (Additional file 1: Fig. S10A) (Additional file 12: Table S11). The lack of a clear distribution pattern is most likely attributable to the distinct anatomical microenvironment of the 5ALA-based Inv margin representing a predominantly normal brain as histologically defined.

**Fig. 5:**
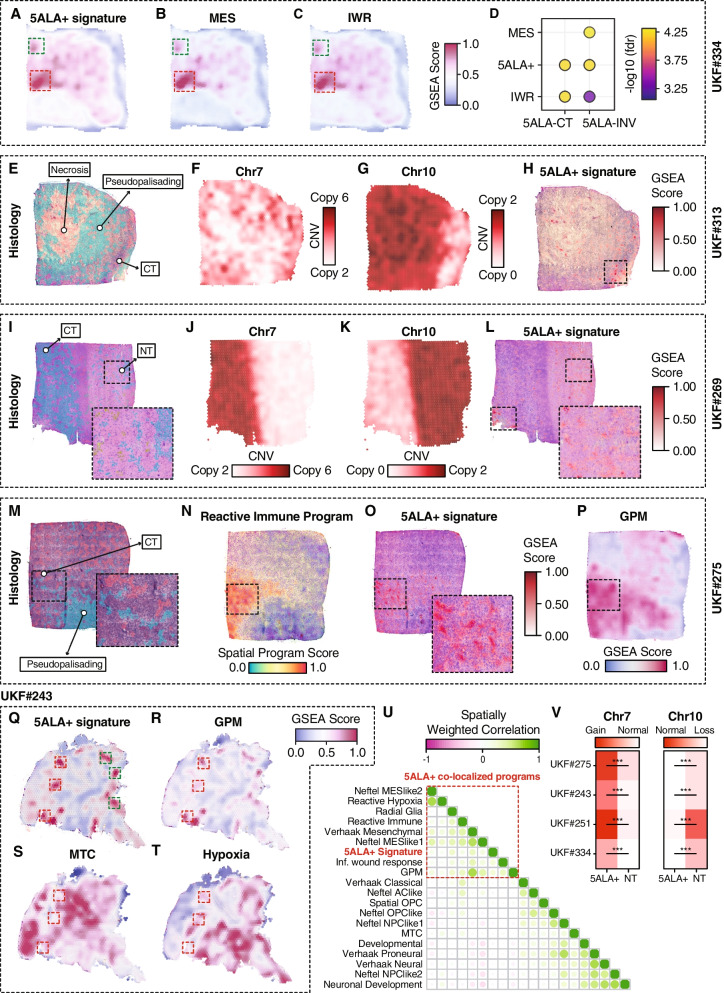
Identification of rare infiltrative 5ALA + cell cluster within GBM invasive margin. GSEA score of 5ALA + gene signature (**A**), MES (**B**), and Inf. wound response (**C**) are shown for patient sample UKF#334, with enriched (red) and random (blue) spots. Spatial localization of the 5ALA + gene signature is marked (dashed box), where red (5ALA-INV) and green (5ALA-CT) boxes represent 5ALA + enriched spots. GSEA between 5ALA-CT and 5ALA-INV is shown as a bubble plot (**D**). Enrichment scores (− log_10_ FDR) are color-coded (yellow—high enrichment; black—low enrichment). H&E-stained tissue section of patient UKF#313 showing the necrotic core, pseudopalisading cells, and cellular tumor (CT) regions (**E**). Inferred CNV analysis of chromosomes 7 (**F**) and 10 (**G**) presented by a spatial surface plot where gain and loss of chromosomes 7 and 10 respectively are color-coded. GSEA of 5ALA + gene signature illustrated by a surface plot where GSEA score is depicted by color code and 5ALA + enriched spots marked (dashed box) (**H**). H&E-stained tissue section of patient UKF#269 showing CT and adjacent non-tumor (NT) regions (**I**). Inferred CNV analysis of chromosomes 7 (**J**) and 10 (**K**) is shown by a surface plot. GSEA of 5ALA + gene signature is shown by a surface plot where 5ALA + enriched spots are marked (dashed box) (**M**). H&E-stained tissue section of patient UKF#275 showing CT with surrounding pseudopalisading cells (**M**). The scaled spatial program score indicates the expression of the reactive immune program (**N**). GSEA score representing spatial arrangements of 5ALA + gene signature (**O**) and GPM (**P**) enriched spots (dashed box). Spatial locations of 5ALA + enriched spots are represented by the GSEA score where red indicates high enrichment of 5ALA + gene signature (**Q**). The 5ALA-INV and 5ALA-CT spots are indicated by red and green boxes, respectively. GSEA scores representing GPM (**R**), MTC (**S**), and hypoxia (**T**). Spatially weighted correlation analysis and spatial overlap of 5ALA + gene signature with established transcriptional signatures (**U**). Gene sets with high cross-correlation with 5ALA + gene signature are marked (dashed box) (**U**). CNV analysis of Chr7 (left) and Chr10 (right), across four samples (UKF#275, UKF#243, UKF#251, and UKF#334) (V). The color code indicates the gain or loss of chromosomes. Each box represents the average CNV value from 50 selected spots across 5ALA and NT regions. The stars indicate the significance of the *p*-value < 0.001

We, further, interrogated the spatial-localizations and surrounding microenvironments that harbor these cells using spatially resolved transcriptomics (stRNA-seq) analysis of tissue sections from an independent *IDH-wt* GBM patient cohort (*N* = 16) [51]. UKF#334 was chosen as a representative tissue sample illustrating the co-localization of 5ALA + transcriptional features (Fig. 5A), with MES (Fig. 5B) and Inf. wound response (Fig. 5C) gene signatures. In addition, 5ALA + enriched cells exhibited spatial proximity to regions with an enriched GPM metabolic state (Additional file 1: Fig. S10B), and to regions of hypoxic activation (Additional file 1: Fig. S10C). The concordant spatial co-localization of GPM in close relation to hypoxia provided a plausible explanation for the active GPM state of 5ALA + cells as a compensatory mechanism in response to metabolically altered environments. Corroborating our previous findings, 5ALA + cells were further characterized by high expression of *CD44* (Additional file 1: Fig. S10D). Interrogation of transcriptional heterogeneity through spatial clustering revealed the presence of distinct clusters (*N* = 11) (Additional file 1: Fig. S10E). Among the clusters, cluster 9 and cluster 7 predominantly showed a spatial overlap with the 5ALA + enriched region, implying the heterogeneous transcriptome landscape manifests within the 5ALA + cell population.

Next, we uncovered the spatial localization of the Invasive margin, through inference of copy number variations (CNVs), where the cellular tumor (CT) region was characterized by a gain of chromosome 7 (Additional file 1: Fig. S10F) and adjacent non-tumor (NT) region was delineated by high expression of *RBFOX3 (*NeuN) (Additional file 1: Fig. S10G). 5ALA + spots were predominantly localized in close proximity to the Invasive margin (5ALA-INV) separating the CT and NT regions (Fig. 5A and Additional file 1: Fig. S10H). However, a 5ALA + signal was also detected within the CT region (5ALA-CT) (Fig. 5A and Additional file 1: Fig. S10H). These distinct spatial locations of the 5ALA + spots raise the question as to what unique characteristics define 5ALA-INV from 5ALA-CT. To test the hypothesis that 5ALA-INV possesses unique defining transcriptional features, we segmented the spatially distinct 5ALA regions (5ALA-INV from 5ALA-CT) (Additional file 1: Fig. S10H). DEG analysis followed by GSEA revealed that 5ALA-INV activates transcriptional programs including reorganization of collagen assembly, extracellular matrix, and epithelial to mesenchymal transition, necessary for cell migration/invasion (Additional file 1: Fig. S10I). Both 5ALA-INV and 5ALA-CT were enriched in 5ALA + and Inf. wound response gene signatures (Fig. 5D), whereas 5ALA-INV was enriched in MES (Fig. 5D), further providing evidence that 5ALA + invasive margin cells may undergo a transition to the MES subtype.

Previously, it was reported that GBM cells with MES subtype reside within the tumor core, near the necrotic zone, and in the presence of blood vessels [59, 65–67]. Our results also highlighted that the bulk-derived MES signature was not solely restricted to the Inv margin but was also present within the tumor core across different tumor tissue samples (Additional file 1: Fig. S10J and S10K). In addition, high enrichment of the hypoxia-dependent MES2 subtype was identified within the tumor core (Additional file 1: Fig. S10L) and peri-necrotic region (Additional file 1: Fig. S10M), corroborating previously reported results. In contrast, MES1 enriched spots were identified in the Inv margins (Additional file 1: Fig. S10N). Furthermore, the minimal or no overlap of genes among the mesenchymal gene sets (MES, MES1, and MES2) (Additional file 1: Fig. S10O) also contributed to the varied spatial localization across the GBM tissues.

To gain a deeper understanding of how distinct the localization and spatial microenvironment of 5ALA + cells are, relative to the necrotic tumor core, we further utilized the stRNA-seq data to identify 5ALA + gene signature enriched regions within specific tissue samples. We selected sample UKF#313 representing a necrotic tumor core, surrounded by pseudopalisading cells, followed by a CT region (Fig. 5E). Inference of CNV provided further evidence signifying the gain of chromosome 7 (Fig. 5F) and loss of chromosome 10 (Fig. 5G) in the CT. GSEA analysis revealed that spots enriched with the 5ALA + gene signature in the cellular regions were spatially distant from the necrotic core (Fig. 5H).

In sample UKF#269, histology analysis showed a distinction between CT and spatially adjacent NT regions (Fig. 5I). The spatial boundary between CT and NT was also underpinned by CNV analysis, where CT was characterized by the gain of chromosome 7 (Fig. 5J) and loss of chromosome 10 (Fig. 5K). The spatial distinction between CT and NT was further confirmed by the high expression of *MKI67* (Ki67) (Additional file 1: Fig. S10P) and *RBFOX3* (NeuN) (Additional file 1: Fig. S10Q) in CT and NT regions, respectively. In this tissue sample, 5ALA + enrichment was identified within a localized small CT cluster (Fig. 5L). Interestingly, a relatively weak enrichment of 5ALA + gene signature was also observed in the NT region where a few intermittent 5ALA + enriched spots exhibited a more diffused pattern (Fig. 5L). This result presented clear evidence of infiltrative 5ALA + cells present within the adjacent NT brain region that is spatially distinct from CT.

A further spatially distinct niche for 5ALA + spots was revealed in sample UKF#275, where 5ALA + cells were co-localized with immune cells. Histology (Fig. 5M) coupled with CNV analyses—gain of chromosome 7 (Additional file 1: Fig. S11A) and loss of chromosome 10 (Additional file 1: Fig. S11B), aided spatial location determination of the CT region within the tissue. Within the reactive immune zone (Fig. 5N), the presence of 5ALA + enriched spots (Fig. 5O) was identified, indicating co-occurrence of infiltrative 5ALA + cells with immune cells (Additional file 1: Fig. S11C) further corroborating our previous findings that the 5ALA + population may harbor myeloid cells. Reactive immune was previously characterized by the inflammation-associated genes (e.g., *HLA-DRA, C3, CCL4*, and *CCL3*) [51]. This result underscores the unique potential utility of 5ALA + fluorescence-guided surgery to demarcate an immune reactive microniche consisting of both tumor and myeloid cells beyond the Core.

To better understand the spatial architecture of the distinct metabolic programs, localization of the GSEA scores was calculated for GPM, hypoxia, MTC, and 5ALA + cells. The 5ALA + spots (Fig. 5O) were found to be enriched with the GPM metabolic state (Fig. 5P). Interestingly, unlike, UKF#334, this tissue sample revealed that although the 5ALA + region exhibited a relatively lower enrichment of the hypoxia response transcriptional program, the major hypoxia enriched region was spatially distinct from 5ALA + cells (Additional file 1: Fig. S11D), implying that 5ALA + cells retain a GPM state even in the presence of weak hypoxic strain. In contrast, the MTC metabolic state was distantly located from both the 5ALA + and hypoxic regions (Additional file 1: Fig. S11E). Akin to sample UKF#275, co-localization of 5ALA + spots with reactive immune cells was also observed within the CT region for sample UKF#251 (Additional file 1: Fig. S11F-I). These results indicated that irrespective of the strong or weak hypoxic strain, 5ALA + cells have the ability to activate a glycolysis-dependent GPM metabolic state.

Sample UKF#243 was further analyzed where inferred CNVs (gain of chromosome 7 (Additional file 1: Fig. S11J) and loss of chromosome 10 (Additional file 1: Fig. S11K) clearly defined CT and NT regions. Notably, in this sample, reactive immune was localized both in the NT and infiltrative margin (Additional file 1: Fig. S11L). 5ALA + enriched spots were identified in both the Invasive margin and spatially distant locations deep within the CT region (Fig. 5Q). Enrichment analysis of metabolic programs showed that 5ALA + cells harbored active GPM (Fig. 5R), but not MTC (Fig. 5S) signatures, corroborating our previous findings. Rather, MTC was enriched in the NT region (Fig. 5S) characterized by low hypoxic strain (Fig. 5T). Seurat clustering of UKF#243 resulted in nine distinct clusters where 5ALA + enriched regions exclusively co-localized with Cluster-5 (Additional file 1: Fig. S11M). DEG analysis revealed that the top differentially expressed genes included Inf. wound response genes (*CXCL8, CXCL3, RGS1,* and *RNASET2*) in Cluster-5 (Additional file 1: Fig. S11N). Enrichment analysis showed that Cluster-6, representing the cells aligned with Invasive margin was enriched in MHC protein complex-associated pathways, whereas innate immune systems and negative regulation of cell population proliferation pathways, were enriched in Cluster-5 encompassing the 5ALA + spots (Additional file 1: Fig. S11O).

To explore the spatial proximity of 5ALA + gene signature with established transcriptional programs, we performed a quantitative analysis based on the patient-wise spatially weighted correlations which were horizontally integrated for robust prediction of adjacent transcriptional activation (Fig. 5U). 5ALA + spots (55 μm) shared co-localizations with radial glia (RG), mesenchymal (MES and MES1-like), Inf. wound response, GPM, and reactive immune transcriptional programs (Fig. 5U). The Cartesian co-occurrence of the reactive immune and 5ALA + transcriptional program suggests that 5ALA + cells may reside in the same microenvironment shared with myeloid cells. Interestingly, these results highlighted the potential use of 5ALA-induced PpIX fluorescence-guided surgical procedure to identify the immune reactive microniche beyond the Core, where tumor cells can potentially interact with myeloid cells.

To demonstrate the malignant nature of 5ALA + cells, we quantified the chromosomal gain and loss of 5ALA + enriched spots compared to adjacent NT regions. A subset of samples (*N* = 4, UKF#275, UKF#243, UKF#251, and UKF#334) were used for which there was a clear spatial distinction between 5ALA + and NT regions. The spots within the 5ALA + region were sorted according to the 5ALA + GSEA score. The number of 5ALA + spots showed a high degree of variability in a patient-specific manner. Among the tissue samples, we identified a minimum of 50 ALA + enriched spots across all the tissues, and therefore the top 50 spots were selected. An equal number of NT spots (*N* = 50) were selected for which the 5ALA + gene signature was not enriched. The comparison revealed that 5ALA + spots showed a gain of Chr7 (*p*-value < 0.001) and a loss of Chr10 (*p*-value < 0.001) compared to NT spots (Fig. 5V). Corroborating our previous analysis (Fig. 3E), spatial CNV analysis clearly demonstrated that the 5ALA + spots harbored malignant cells with CNVs, although non-malignant myeloid cells can co-localize in 5ALA + spots.

In conclusion, stRNA-seq analyses unveiled the existence of the *CD44* expressing rare population of infiltrative 5ALA + cells residing in the Invasive margin surrounding a hypoxic microniche, distant from the necrotic core and which co-localizes with myeloid cells.

### Enriched cellular, transcriptional, and metabolic states of 5ALA + cells are associated with tumor recurrence

**Fig. 6:**
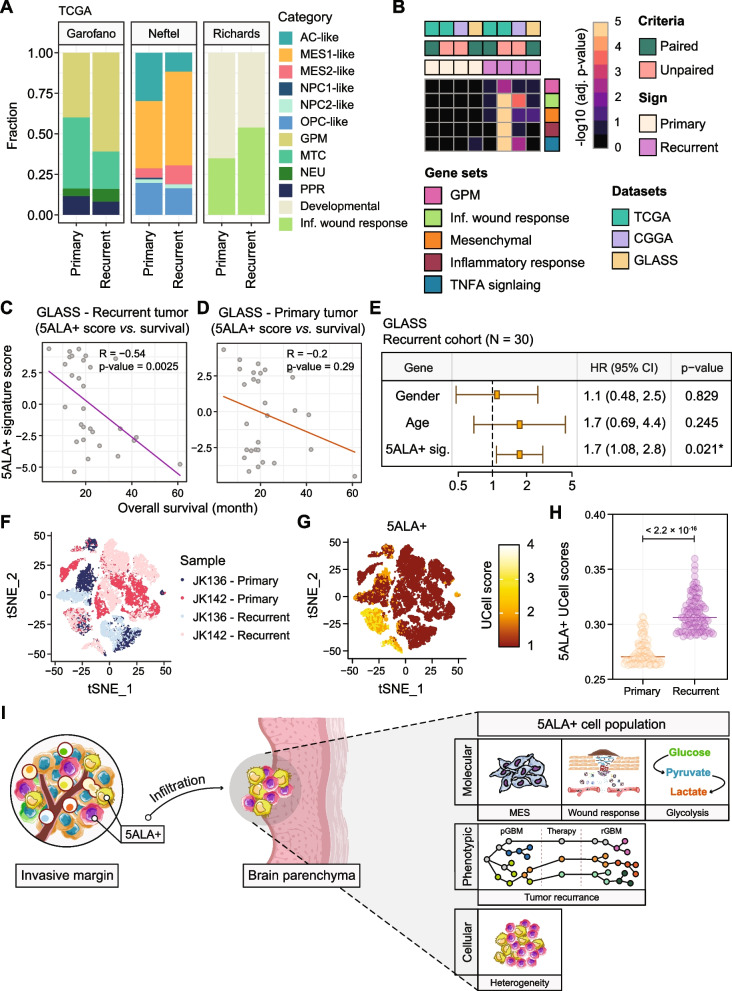
Association of 5ALA + gene signatures with recurrence in GBM patients. Stacked bar plots representing the CIBERSORTx-derived average cell-type estimates from Netfel, Richards, and Garofano et al. datasets across primary and recurrent GBM patients from TCGA (**A**). Heatmap showing the − log10 adjusted *p*-value of the enriched gene signatures across recurrent and primary GBM tumors (**B**). The conditions representing the comparisons between recurrent and primary GBM tumors from TCGA and CCGA are given in columns (TCGA—Recurrent *vs.* Primary and CCGA—Recurrent *vs.* Primary). GBM data from TCGA has been analyzed in a paired and unpaired manner. Columns are divided into upregulated (Red) or downregulated (Blue) segments based on the regulation of genes between Recurrent *vs.* Primary samples. Each row represents the different gene signatures. Scatter plots representing the correlation between 5ALA + gene signature scores and overall survival (months) in recurrent and primary patients from GLASS (**C** and **D**) cohort. Spearman correlation coefficient (R) and *p*-values are shown. Forest plot representing the hazard ratio of different factors in recurrent GBM patients (**E**). tSNE coupled with Louvain clustering of the primary and recurrent GBM cells from two patients (**F**). 5ALA + UCell score mapping onto the tSNE (**G**). Comparison of 5ALA + UCell score between primary and recurrent cells (**H**). (Bar represents the mean). Schematic diagram showing the 5ALA + cells with MES subtype and distinct transcriptional programs such as wound response signatures are associated with recurrence of GBM tumor (**I**)

To identify the patients with a higher fraction of 5ALA + cellular and metabolic states (MES, Inf. wound response, and GPM) from the TCGA-GBM cohort, we performed deconvolution of the TCGA-derived bulk transcriptome dataset using CIBERSORTx algorithm. Cell-type fractions were estimated based on scRNA-seq datasets from Neftel et al. [37], Richards et al. , and Garofano et al. [9]. The cellular and metabolic states were estimated for each of the primary (*N* = 154) and recurrent (*N* = 13) GBM patients from the TCGA cohort by using signature matrices generated from Neftel (Additional file 1: Fig. S12A), Richards (Additional file 1: Fig. S12B), and Garofano et al. (Additional file 1: Fig. S12C). For unequal sample numbers, a bootstrap method followed by the Wilcox test was carried out between recurrent and primary samples. Interestingly, recurrent tumor samples showed a higher fraction of GPM metabolic state (*p*-value = 0.014), Inf. wound response transcriptional program (*p*-value = 0.048), and MES1-like cellular state (*p*-value = 0.0533), compared to primary tumors (Fig. 6A).

To further explore the impact of the 5ALA + enriched gene signatures on the recurrence of GBM, GSEA was performed on RNA-seq data obtained from primary and recurrent *IDH*-wt patients of TCGA, CGGA, and GLASS cohorts (Additional file 13: Table S12). Primary tumors only exhibited marginal enrichment of pathways such as hypoxia, DNA repair, and E2F targets, whereas a diverse array of pathways including oxidative phosphorylation, MYC targets, fatty acid metabolism, IL6/JAK/STAT signaling, epithelial-mesenchymal transition, TNF-α signaling via NFкB, and inflammatory response were enriched at recurrence, and where the two latter pathways were also enriched in 5ALA + cells (Additional file 1: Fig. S12D). TCGA harbors an unequal sample distribution between primary (*N* = 154) and recurrent (*N* = 13) tumor samples. Among the thirteen recurrent GBM samples, six were paired with primary tumor data. Therefore, paired and unpaired analyses were performed for TCGA (Additional file 1: Fig. S12D). For CGGA and GLASS cohorts, primary and recurrent tumor samples are unpaired and paired, respectively. Enrichment analysis revealed an intriguing phenomenon where 5ALA + gene signature enrichment was observed in recurrent but not in primary tumors. For instance, MES was enriched in recurrent tumors across TCGA, CCGA, and GLASS cohorts, and Inf. wound response was enriched in recurrent GBM of TCGA and GLASS cohorts but not in CGGA (Fig. 6B). Compared to recurrent tumors, no gene signatures were enriched in the primary tumors of TCGA (Fig. 6B). To further investigate the impact of unique 5ALA + gene signatures on the survival of recurrent and primary GBM patients, a single-sample GSEA (ssGSEA) was performed on recurrent and primary tumors separately for the 5ALA + gene signatures. By combining the NES, a combined 5ALA + gene signature score was calculated for each tumor sample, followed by correlation analysis with survival data of recurrent and primary patients. For recurrent tumors of GLASS (Fig. 6C) cohorts, 5ALA + gene signature scores exhibited a significant negative correlation with survival, whereas primary tumors did not (Fig. 6D). Analysis with TCGA cohort corroborated this result signifying that 5ALA + gene signature is highly correlated with the survival of recurrent GBM patients (Additional file 1: Fig. S12E and S12F). We then performed Cox regression on the matched-recurrent (*N* = 30) and -primary (*N* = 30) samples from GLASS cohort (Additional file 13: Table S12). Apart from 5ALA + -specific gene signatures, we included confounding factors such as the age and gender of the patients. Firstly, a univariate Cox regression analysis revealed that 5ALA + gene signature, Inf. wound response, MES, and Inf. response were significantly associated with poor survival in the recurrent cohort (Additional file 13: Table S12). The age and gender of the patients were not associated with survival (Additional file 13: Table S12). In contrast, no significant association was identified in primary GBM patients (Additional file 13: Table S12). Next, a multivariate regression analysis revealed that the combined 5ALA + gene signature was significantly associated with poorer survival of the recurrent GBM patients (Fig. 6E). In contrast, no significant association was observed for primary GBM patients. Due to the highly unequal number of TCGA recurrent (*N* = 154, Additional file 1: Fig. S12E) and primary (*N* = 13, Additional file 1: Fig. S12F) samples, the interpretation of the Cox regression analysis is challenging and hence was not performed on TCGA cohort. In an ideal setting, the experimental validation of the role of 5ALA + cells in the recurrence of GBM may include patient-matched longitudinal samples from primary and recurrent patients. The process of collecting patient-matched primary and recurrent samples is challenging and time-consuming; moreover, only a small percentage of GBM patients receive recurrence surgery [68]. In addition, resectable recurrent tumors tend to exhibit a higher rate of necrosis rendering molecular analysis challenging [69]. However, patient-derived explants (PDEs) provide a reliable model for molecular analysis of recurrent tumors [70]. To test the hypothesis that the 5ALA + gene signature is associated with recurrent GBM at single-cell resolution, we took advantage of the scRNA-seq dataset from matched primary and recurrent tissues from two GBM patients where the resected tissue samples were used to generate the patient-derived explants (PDEs) [70]. We retrieved the scRNA-seq data representing primary (*N* = 10,905) and recurrent (*N* = 8833) cells from PDEs. tSNE analysis coupled with Louvain clustering (Fig. 6F) followed by the mapping of 5ALA + UCell score onto the tSNE plot showed that a 5ALA + gene signature was markedly visible in a distinct cluster consisting of primary and recurrent tumor cells from the GBM patients (Fig. 6G). The comparative analysis confirmed our hypothesis that, recurrent GBM cells have significantly higher 5ALA + UCell scores compared to primary ones (*p*-value < 2.2 × 10^−16^) further underscoring the association between 5ALA + gene signature and GBM recurrence (Fig. 6H).

In conclusion, the enriched gene signatures of 5ALA + cells were associated with poor survival and recurrence in GBM, signifying the probable functional impact of these signatures in tumor progression and interval to disease recurrence (Fig. 6I).

## Discussion

We have unlocked cellular, transcriptional, and metabolic signatures of the 5ALA + population residing within the GBM Invasive margin that are distinct from the tumor Core. SPRP complemented by scRNA-seq and spatially resolved stRNA-seq revealed that the infiltrative 5ALA + cell population consisting of MES GBM and myeloid cells with mesenchymal subtype, active wound response pathway, and glycolytic metabolic states resides within a microenvironment characterized by patient-specific strong or weak hypoxic strain. The transcriptional signatures that define the infiltrative 5ALA + cell population are associated with poor survival and tumor recurrence, implicating 5ALA + cells as an accurate a priori proxy of GBM residual disease post-surgery.

Previously, it has been hypothesized that 5ALA-induced PpIX fluorescence beyond the T1 enhancing region on magnetic resonance imaging (MRI) represents a unique microenvironment contributing to molecular signatures distinct to tumor Core and Rim [2]. Extending this hypothesis, we showed that pro-proliferative pathways (e.g., G2M checkpoint, mTOCR1 signaling, and E2F targets) were highly enriched in the Core but absent in the infiltrative margin (Fig. 1I).

Enrichment of the NeuN cell type in both unsorted Invasive margin and 5ALA − cells indicates that normal neural cells constitute the majority of the infiltrative margin (Fig. 2E), consistent with previous findings [71]. One of the striking features of 5ALA + cells was the lack of molecular identity to canonical neural cell types, underscoring the unique transcriptional landscape of infiltrative GBM. Using a microarray-based study, Bonnin et al. also showed that 5ALA + enriched tumor tissue failed to exhibit molecular signatures of any known neural cell types [72].

Emerging evidence supports GBM molecular subtype heterogeneity and indicates that subtype switching to MES in particular is associated with recurrence and poor survival outcomes [11, 13]. To extend this hypothesis, we showed that the heterogeneity of GBM molecular subtypes is not restricted to recurrence, but can manifest in a region and cell-type-specific manner. Enrichment of the 5ALA + population with the MES subtype (Fig. 2A, B) supports a hypothesis that an infiltrative MES subtype may drive GBM recurrence. Indeed, as determined by stRNA-seq analysis, the 5ALA + invasive subpopulation (5ALA + enriched spots outside the tumor core: 5ALA-INV) was enriched in MES subtype (Fig. 5D). Interestingly, a link between an MES gene signature and decreased tumor purity has been established as a common theme across different cancers [73, 74]. One of the prominent features of the MES subtype is the association with immune-related pathways and the lower purity score in comparison to PM and CL, highlighting the possible infiltration of non-neoplastic and immune cells into MES GBM [11, 75].

Previously, Jin et al. [59] showed that tumor cells from the enhancing region (ER), characterized by the disruption of the blood–brain barrier at areas of angiogenesis, exhibit high expression of proneural genes, whereas the necrotic region (NR; hypoxic) exhibits a high expression of MES genes. However, this study lacked 5ALA-induced PpIX fluorescence guidance to define the invasive margin characterized by MRI T2 high signal distantly localized from the Core (non-enhancing on T1 with gadolinium), representing the furthest region from the tumor core where the 5ALA + signal fades into the background of non-neoplastic cells (Fig. 1B–D). The “Invasive margin” identified by the 5ALA-induced PpIX fluorescence method is therefore spatially distinct from the radiologically determined enhancing region (ER) and enhanced margin (EM). Since the Inv margin defined by our study is localized in a spatially distinct region, the presence of an infiltrative 5ALA + population with MES subtype presents an interesting insight into the biology of the Inv margin. However, our results do not contradict the previous report that MES GBM cells can be found within the Core region. Indeed, our spatial analysis revealed that MES subtype GBM cells are localized in diverse microniches including Core, peri-necrotic region, and Inv margin (beyond the Core) (Additional file 1: Fig. S10I-M). This proposes an interesting scenario regarding the origin of MES subtype infiltrative GBM cells. Whether they originate from the tumor core by cells with infiltrating capacity escaping hypoxic stress and migrating towards the invasive margin (where a strong hypoxic environment is absent [76]) remains to be explored. Interestingly, 5ALA + cells within the Inv margin activate other transcriptional features such as glycolysis-dependent GPM metabolic pathway and Infl. wound response. The local microenvironment of the 5ALA + region is likely to be conducive to the interactions between malignant and myeloid cells and may aid malignant cells to acquire a unique combination of transcriptional features [67].

The ability of the 5ALA + population to retain a glycolytic metabolic state within Invasive margin (Fig. 2H and Additional file 1: Fig. S10A) raises another important question regarding hypoxic stress within this microenvironment. Despite the absence of hypoxia as inferred by CD31 immunostaining (Fig. 1P) and GSEA (Fig. 1I), the enrichment of glycolysis-dependent GPM in 5ALA + cells appears contradictory. However, the spatial analysis provided a plausible explanation by revealing that 5ALA + cells reside within a localized microniche within Invasive margin which is under strong hypoxic strain (Additional file 1: Fig. S10B). The retaining of analogous glycolytic metabolic states from the tumor Core may also render a survival advantage to the infiltrative 5ALA + cells. Metabolic reprogramming may allow the 5ALA + cells to cope with the energy demands required for invasion and colonization of the surrounding brain tissue. Moreover, enrichment of a glycolysis-dependent GPM metabolic signature was only observed in the 5ALA + population but not in 5ALA − cells (Fig. 2H, I). This refutes the notion that transcriptional signatures are artifactually established due to the process of FACS and supports the claim that these signatures reflect the biology of infiltrative margin residual disease. It is important to note that the unsorted invasive margin tissue is not the exact same tissue fragment that was processed by FACS (i.e., one invasive margin fragment was snap frozen, and a second fragment was immediately enzymatically dissociated and FACS processed). This spatial difference between the Inv margin and the proximate tissue from where the 5ALA + / − cells were isolated could contribute to some transcriptome differences.

Spatial transcriptomics did not provide single-cell resolution as multiple cells are typically present in each spot (55 μm in size). Therefore, it is possible that spots enriched with the 5ALA + signature may also harbor cell types other than malignant cells such as myeloid cells. Moreover, the co-localization of the reactive immune and 5ALA + gene signature (Fig. 5N, O) raises the possibility that the 5ALA + population encompasses MES GBM cells that may reside in the same microniche shared with myeloid cells corroborating CIBERSORTx findings (Fig. 3G). A previous study showed that apart from tumor cells, the infiltrating immune cells of myeloid lineage (especially macrophages and microglia) have the capacity to uptake and metabolize 5ALA by activating iron metabolism pathways [62]. This new finding underscores the possibility that the 5ALA + population localized distantly from the tumor core is heterogeneous in nature consisting of both infiltrative MES tumor and myeloid cells. The tumorigenic potential of the 5ALA + population as shown by our earlier in vivo study through subcutaneous xenograft implants [14] may stem from the malignant fraction of the 5ALA + population. Interestingly, the co-localization of the tumor and myeloid cells within the 5ALA + region also highlights the possibility of ligand-receptor-mediated cellular interactions which may be functionally associated with the capability of tumor cells to acquire the MES subtype as reported previously [67]. The interactions between distinct tumor subpopulations and the surrounding normal brain govern the tumor microenvironment, which is likely to facilitate the adaptation of different subpopulations to external selection pressures including treatment-induced stress. It has been reported that GBM cells deploy an epigenetic immunoediting process allowing these cells to mimic the myeloid cell-specific transcriptional program [60]. Furthermore, Hara et al. showed that macrophages have the potential to induce a transition of GBM cells into the MES state [67]. The induction is mediated by the interaction between the macrophage-derived oncostatin M (OSM) and OSM receptors in complex with GP130 on GBM cells [67]. In light of these findings, the myeloid cells are more likely to play a crucial role in the transition of the malignant cells into the MES subtype within the 5ALA + Inv region.

In contrast to the classification of GSC subtypes into proneural and mesenchymal states [11] or based on similarity to neural subtypes in normal or fetal brain development [77], Richards et al. proposed that GSC heterogeneity may originate from the transcriptional gradient composed of neural Developmental and Inf. wound response programs. Our study offers an important insight by revealing that infiltrative GBM cells with the MES subtype have an active Inf. wound response transcriptional program, whereas the remainder of the Invasive margin was devoid of Inf. wound response pathway activation. stRNA-seq analyses showed that Inf. wound response activation was more confined to the 5ALA + micro-niche indicating the unique ability of these rare infiltrative GBM cells to activate the neural wound healing pathway to promote and maintain growth. Upregulation of exonic levels for MES and Inf. wound response genes in 5ALA + relative to 5ALA − cells implied that these genes were under active post-transcriptional control.

The shared transcriptional regulatory network that governs GBM transition to MES and recurrence has not been previously elucidated, partly due to an inability to identify and characterize infiltrative GBM subpopulations exhibiting MES. Previous studies [59, 65–67] report that GBM cells with MES subtype reside within the tumor core, near the necrotic zone, and in blood vessels. However, none of these studies utilized 5ALA-induced PpIX fluorescence to determine the invasive margin that is ethically safe to collect surgical biopsies from, and where the 5ALA + signal fades into the background of non-neoplastic cells. As described previously, our results suggest the presence of an infiltrative 5ALA + population with MES signature outside the MRI contrast-enhanced Core (referred to as Inv margin in the current manuscript) that may represent a distinct localization with respect to prior studies.

The transition of GSCs to MES was reported to be dependent on TNF-α signaling via NFкB pathway [13], which was also upregulated in the infiltrative 5ALA + population (Fig. 1I). Interestingly, among the TFs, two NFкB family members—*NFkB1* and *REL*—in addition to the inhibitor of NFкB-REL complex NFкBIA, were upregulated in 5ALA + cells (Additional file 1: Fig. S7C). These seemingly opposing factors may establish a delicate balance between inflammatory and anti-inflammatory pathways that are required to maintain a chronic and persistent low level of inflammation, further boosted by the infiltration of anti-inflammatory and regulatory immune cells [78]. The shared transcriptional control of the MES and Inf. wound response genes by *REL* and *NFкB1* in infiltrative 5ALA + cells suggest an association between inflammatory pathways and MES gene signatures.

Previously, the transcriptional similarity between mesenchymal-like tumor cells and myeloid cells has been shown [67]. The current study, aiming to characterize the invasive 5ALA + population localized outside the MRI contrast-enhanced tumor Core region, revealed malignant and myeloid cells sharing similar transcriptomic landscapes within a shared microenvironment, corroborating the emerging evidence. This heterogeneous nature of the 5ALA + population encompassing MES malignant and myeloid cells is also corroborated by the recent evidence based upon scRNA-seq of the 5ALA + population [61] and two-photon excitation fluorescence microscopy [18], showing that 5ALA treatment is efficient to label the tumor tissue from surrounding normal brain but may not be exclusively specific for tumor cells [18, 61]. By combining scRNA-seq and live cell imaging with SCOPE-seq2, it has been proposed that non-malignant myeloid cells within the glioma microenvironment can also be labeled by PpIX (a fluorescent metabolite of 5ALA) in resected tissues from GBM patients who received 5ALA [61].

It is important to understand that the non-exclusivity of 5ALA signal to malignant cells is not limiting the utility of 5ALA-guided surgery; rather the 5ALA + region representing the co-occurrence of MES GBM and myeloid cells outside the tumor core ushers a new window of opportunity to identify a distinct microniche where the interactions between MES GBM and myeloid cells can be studied in relation to tumor recurrence a priori. Moreover, the utility of the 5ALA-guided surgery leading to a greater resection extent with a survival benefit has been determined by clinical trials [79]. Irrespective of the 5ALA-signal not being exclusive to tumor cells in the infiltrative margin, there is clearly a prominent 5ALA + tumor population, the removal of which is associated with a survival benefit.

We show that 5ALA + -specific gene signatures representing MES and Inf. wound response were enriched in recurrent GBM tumors relative to primary tumors and associated with poor survival outcomes (Fig. 6B, E). The close resemblance of the 5ALA + cell population to recurrent tumors in terms of acquisition of the MES subtype (Fig. 6B–E) offers a unique opportunity to explore 5ALA + transcriptional networks further, elucidate biomarkers predictive of recurrence interval, and identify putative molecular therapeutic targets a priori, to initiate treatment in advance of radiologically defined macroscopic recurrence.

## Conclusions

Overall, our study has comprehensively characterized the unique cellular, transcriptional, and metabolic features of the 5ALA + population within the GBM infiltrative margin and underscored the possibility that infiltration of MES malignant and myeloid cells into adjacent brain tissue may be a critical determinant of GBM recurrence. Our findings encourage the neuro-oncology research community to prioritize this infiltrative 5ALA + subpopulation(s) for both mechanistic pre-clinical modeling and to expedite next-generation molecular targeted drug screening. Characterization of the 5ALA + infiltrative subpopulation offers an opportunity to develop more effective GBM treatments and urges focus away from the GBM proliferative core, upon which failed targeted therapies have been predicated to date.

### Supplementary Information

**Additional 1:** PDF file, containing the article-associated supplementary figures (Fig. S1-S12). A short descriptional of the supplementary figures is given below. **Fig. S1.** Quality assessment of spatial-resolved bulk RNA profiling (SPRP) dataset. **Fig. S2.** Hallmark gene-set enrichment analysis of SPRP profile. **Fig S3.** GBM subtype gene-set enrichment analysis of SPRP profile. **Fig. S4.** Metabolic and transcriptional signature analysis and deconvolution of SPRP.  **Fig. S5.** Single-cell analysis from independent cohorts. **Fig. S6.** Single-cell wise UCell enrichment analysis. **Fig. S7.** Transcription factor – Target gene network analysis. **Fig. S8.** Exon-intron split analysis. Fig. S9. Stemness signature analysis. **Fig. S10.** Spatial segmentation and differential gene expression analysis. Fig. S11. Spatial copy number variation and enrichment analysis. **Fig. S12.** CIBERSORTx derived Cell-type fraction estimation across primary and recurrent GBM patients.

**Additional file 2:** **Table S1.** Clinical, pathological, and molecular characterization of GBM patients, and compiled Gene-signatures.

**Additional file 3:** **Table S2.** Hallmark GSEA and leading gene-edge gene list.

**Additional file 4:**  **Table S3.** Neural cell type GSEA and leading gene-edge gene list.

**Additional file 5:** **Table S4.** GBM-subtype GSEA and leading gene-edge gene list.

**Additional file 6:** **Table S5.** Cellular and metabolic gene-set GSEA and leading gene-edge gene list.

**Additional file 7:** **Table S6.** High-resolution gene expression matrices and Ucell scores of different gene signatures.

**Additional file 8:** **Table S7.** TF-target gene interaction.

**Additional file 9:** **Table S8.** Enrichment of selected gene-signatures in DEGs with significantly altered exon and intron counts in 5ALA+ cells.

**Additional file 10:** **Table S9.** Enrichment of stemness associated gene-signatures.

**Additional file 11:** **Table S10.** Genes correlated with stemness in 5ALA+ cells.

**Additional file 12:** **Table S11.** IVY-Glioblastoma samples with representative 5ALA+ signature expressions.

**Additional file 13:** **Table S12.** GSEA in primary and recurrent tumors.

## Data Availability

Raw RNA-seq data for spatially distinct unsorted GBM regions and 5ALA FACS sorted cells have been deposited at the ArrayExpress with accession number E-MTAB-8743 (https://www.ebi.ac.uk/biostudies/arrayexpress/studies/E-MTAB-8743) [80]. The publicly available datasets used in the current paper are available from GitHub (https://github.com/ccruizm/GBmap) [81] for GBmap, GSE131928 (https://www.ncbi.nlm.nih.gov/geo/query/acc.cgi?acc=GSE131928) [82] for Neftel et al., and EGAS00001004656 (https://ega-archive.org/studies/EGAS00001004656) for Richards et al. [83]. All the codes (R-scripts) used to analyze the data and visualization of the results are available through GitHub (https://github.com/TheSYSTEMScellSIGNALLINGlab/5ALA-Infiltrative-GBM) [84].
