# Supplementary material for: Spatially resolved transcriptomic profiles reveal unique defining molecular features of infiltrative 5ALA-metabolizing cells associated with glioblastoma recurrence
Source: Genome Med. 2023 Jul 11;15:48. doi: 10.1186/s13073-023-01207-1 (PMC10337060; doi:10.1186/s13073-023-01207-1)
Supplement: Supplementary file 1 — Additional 1: PDF file, containing the article-associated supplementary figures (Fig. S1-S12). A short descriptional of the supplementary figures is given below. Fig. S1. Quality assessment of spatial-resolved bulk RNA profiling (SPRP) dataset. Fig. S2. Hallmark gene-set enrichment analysis of SPRP profile. Fig S3. GBM subtype gene-set enrichment analysis of SPRP profile. Fig. S4. Metabolic and transcriptional signature analysis and deconvolution of SPRP. Fig. S5. Single-cell analysis from independent cohorts. Fig. S6. Single-cell wise UCell enrichment analysis. Fig. S7. Transcription factor – Target gene network analysis. Fig. S8. Exon-intron split analysis. Fig. S9. Stemness signature analysis. Fig. S10. Spatial segmentation and differential gene expression analysis. Fig. S11. Spatial copy number variation and enrichment analysis. Fig. S12. CIBERSORTx derived Cell-type fraction estimation across primary and recurrent GBM patients. [file 13073_2023_1207_MOESM1_ESM.pdf]

Fig S1

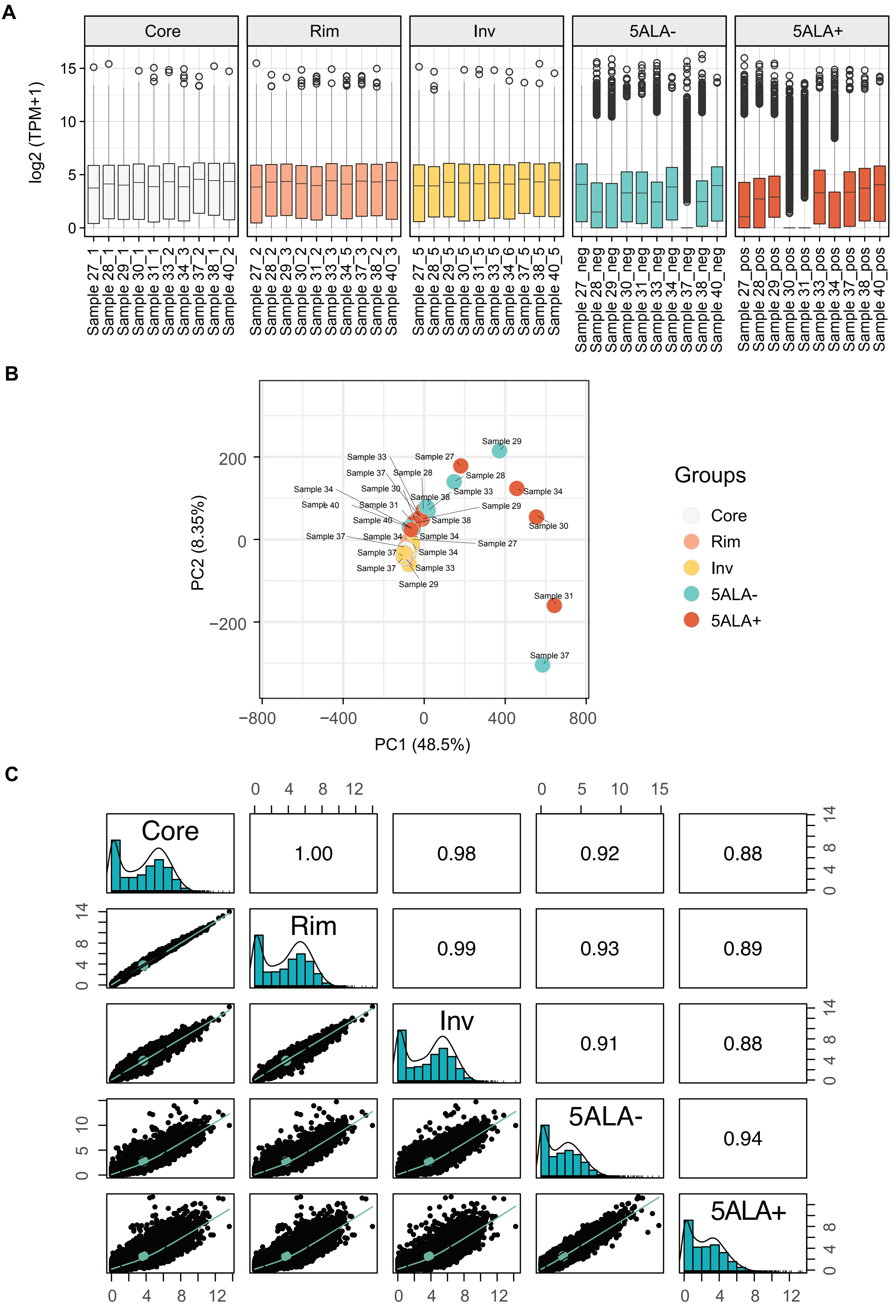
A
Core
Rim
Inv
5ALA-
5ALA+
log2 (TPM+1)
Sample 27_1
Sample 28_1
Sample 29_1
Sample 30_1
Sample 31_1
Sample 33_2
Sample 34_3
Sample 37_2
Sample 38_1
Sample 40_2
Sample 27_2
Sample 28_2
Sample 29_3
Sample 30_2
Sample 31_2
Sample 33_3
Sample 34_5
Sample 37_3
Sample 38_2
Sample 40_3
Sample 27_5
Sample 28_5
Sample 29_5
Sample 30_5
Sample 31_5
Sample 33_5
Sample 34_6
Sample 37_5
Sample 38_5
Sample 40_5
Sample 27_neg
Sample 28_neg
Sample 29_neg
Sample 30_neg
Sample 31_neg
Sample 33_neg
Sample 34_neg
Sample 37_neg
Sample 38_neg
Sample 40_neg
Sample 27_pos
Sample 28_pos
Sample 29_pos
Sample 30_pos
Sample 31_pos
Sample 33_pos
Sample 34_pos
Sample 37_pos
Sample 38_pos
Sample 40_pos
B
PC2 (8.35%)
PC1 (48.5%)
Groups
Core
Rim
Inv
5ALA-
5ALA+
C
Core
Rim
Inv
5ALA-
5ALA+
1.00
0.98
0.92
0.88
0.99
0.93
0.89
0.91
0.88
0.94

Fig. S1: Quality assessment of spatial-resolved bulk RNA profiling (SPRP) dataset

The boxplot of log normalized TPM expression values of 50 samples from 10 patients divided into five brain regions (Core, Rim, Invasive-margin, 5ALA-, 5ALA+) (A). The PCA plot is based on spatially-resolved RNA profiles (SPRP) across 10 GBM patients (B). The scatter plot matrix shows the histogram and correlation coefficients of the global transcriptome without housekeeping genes across brain regions and FACS-sorted cells (C). The lower triangle boxes represent the pairwise scatter plots, the diagonal boxes show the brain regions with the distribution of the values for those regions, and the correlation coefficients are presented in the upper triangle boxes (C).

Fig S2

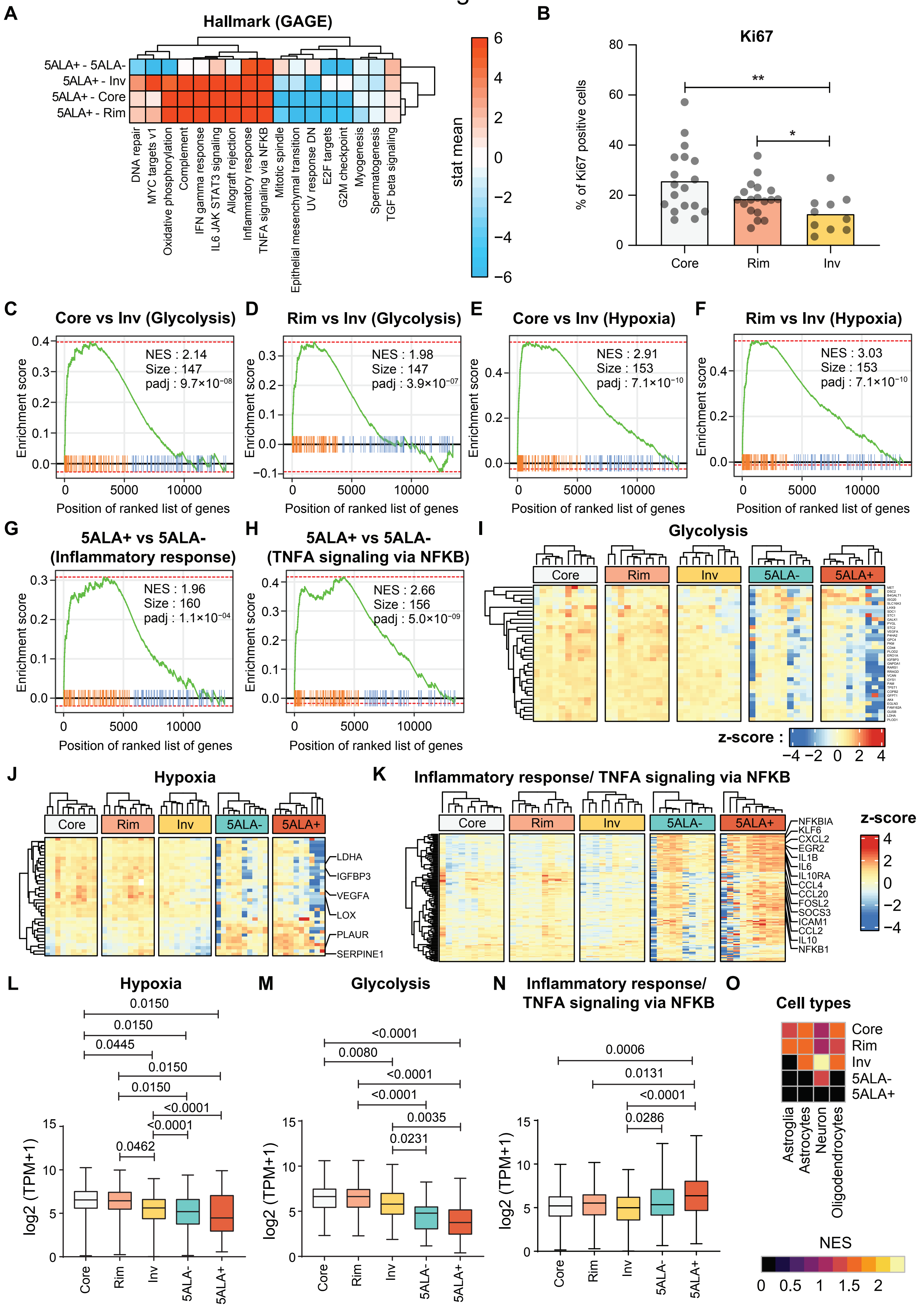
A
Hallmark (GAGE)
5ALA+ - 5ALA-
5ALA+ - Inv
5ALA+ - Core
5ALA+ - Rim
DNA repair
MYC targets v1
Oxidative phosphorylation
Complement
IFN gamma response
IL6 JAK STAT3 signaling
Allograft rejection
Inflammatory response
TNFA signaling via NFKB
Mitotic spindle
Epithelial mesenchymal transition
UV response DN
E2F targets
G2M checkpoint
Myogenesis
Spermatogenesis
TGF beta signaling
stat mean
6
4
2
0
−2
−4
−6
B
Ki67
% of Ki67 positive cells
80
60
40
20
0
**
*
Core
Rim
Inv
C
Core vs Inv (Glycolysis)
NES : 2.14
Size : 147
padj : 9.7×10−08
D
Rim vs Inv (Glycolysis)
NES : 1.98
Size : 147
padj : 3.9×10−07
E
Core vs Inv (Hypoxia)
NES : 2.91
Size : 153
padj : 7.1×10−10
F
Rim vs Inv (Hypoxia)
NES : 3.03
Size : 153
padj : 7.1×10−10
Enrichment score
Position of ranked list of genes
0
5000
10000
G
5ALA+ vs 5ALA- (Inflammatory response)
NES : 1.96
Size : 160
padj : 1.1×10−04
H
5ALA+ vs 5ALA- (TNFA signaling via NFKB)
NES : 2.66
Size : 156
padj : 5.0×10−09
I
Glycolysis
Core
Rim
Inv
5ALA-
5ALA+
z-score :
−4 −2 0 2 4
J
Hypoxia
LDHA
IGFBP3
VEGFA
LOX
PLAUR
SERPINE1
K
Inflammatory response/ TNFA signaling via NFKB
NFKBIA
KLF6
CXCL2
EGR2
IL1B
IL6
IL10RA
CCL4
CCL20
FOSL2
SOCS3
ICAM1
CCL2
IL10
NFKB1
z-score
4
2
0
−2
−4
L
Hypoxia
0.0150
0.0150
0.0445
0.0150
0.0150
<0.0001
<0.0001
0.0462
log2 (TPM+1)
15
10
5
0
Core
Rim
Inv
5ALA-
5ALA+
M
Glycolysis
<0.0001
0.0080
<0.0001
<0.0001
0.0035
0.0231
N
Inflammatory response/ TNFA signaling via NFKB
0.0006
0.0131
<0.0001
0.0286
O
Cell types
Core
Rim
Inv
5ALA-
5ALA+
Astroglia
Astrocytes
Neuron
Oligodendrocytes
NES
0 0.5 1 1.5 2

Fig. S2: Hallmark gene-set enrichment analysis of SPRP profile

Hallmark enrichment heatmap showing the enriched cancer hallmark pathways based on the significantly differentially expressed genes as identified by Limma (padj<0.05) between 5ALA+ cells and Core, Rim, Invasive-margin, and 5ALA- cells (A). Color code indicates enrichment score where red and blue colors represent positive and negative enrichment. Percentage of Ki67 positive cells across Core, Rim, and Inv regions (B). GSEA enrichment plots of representative gene sets from Glycolysis (Core *vs.* Invasive-margin) (C), Glycolysis (Rim *vs.* Invasive-margin) (D), Hypoxia (Core *vs.* Invasive-margin) (E), Hypoxia (Rim *vs.* Invasive-margin) (F), Inflammatory response (5ALA+ *vs.* 5ALA-) (G), and TNF-α signaling via NFkB (5ALA+ *vs.* 5ALA-) (H). The heatmaps show hierarchical clustering based on z-scored expression (Log_2 TPM+1) of the leading edge genes of Glycolysis (I), hypoxia (J), and inflammatory response/ TNF-α signaling via NFκB(K). Some rows are labeled with the symbols of selected genes of interest. Boxplots illustrating significant expression (Benjamini hochberg < 0.05) changes between brain regions for differentially regulated leading-edge genes (Limma; padj<0.05) from- Hypoxia (L), Glycolysis (M), and Inflammatory response/ TNF-A signaling via NFkB (N). Hierarchical clustering heatmap of brain cell type (oligodendrocytes, neurons, astrocytes, and cultured astroglia) specific enrichment analysis (O). The color intensities depict the normalized enrichment scores (NES) of each cell type on different brain regions (Core, Rim, Invasive-margin, 5ALA-, 5ALA+).

Fig S3

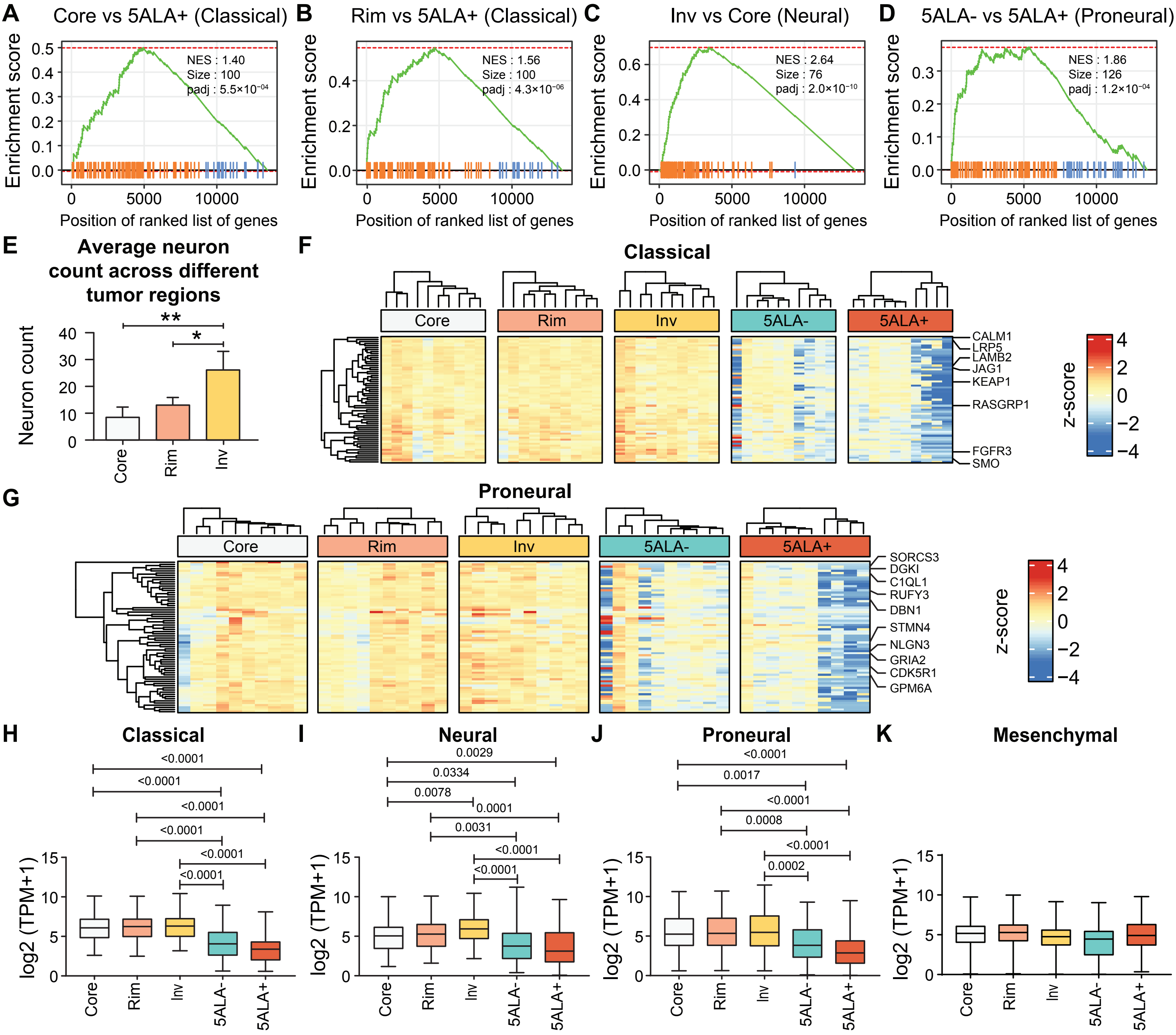

A Core vs 5ALA+ (Classical)
NES : 1.40
Size : 100
padj : 5.5×10⁻⁰⁴
B Rim vs 5ALA+ (Classical)
NES : 1.56
Size : 100
padj : 4.3×10⁻⁰⁶
C Inv vs Core (Neural)
NES : 2.64
Size : 76
padj : 2.0×10⁻¹⁰
D 5ALA- vs 5ALA+ (Proneural)
NES : 1.86
Size : 126
padj : 1.2×10⁻⁰⁴
Enrichment score
Position of ranked list of genes
E Average neuron count across different tumor regions
Neuron count
Core
Rim
Inv
F Classical
Core
Rim
Inv
5ALA-
5ALA+
CALM1
LRP5
LAMB2
JAG1
KEAP1
RASGRP1
FGFR3
SMO
z-score
G Proneural
SORCS3
DGKI
C1QL1
RUFY3
DBN1
STMN4
NLGN3
GRIA2
CDK5R1
GPM6A
H Classical
I Neural
J Proneural
K Mesenchymal
log2 (TPM+1)
<0.0001
0.0029
0.0334
0.0078
0.0001
0.0031
0.0017
0.0008
0.0002

Fig. S3: GBM subtype gene-set enrichment analysis of SPRP profile

GSEA plots of gene clusters from GBM subtypes – Classical (Core *vs.* 5ALA+) (A), Classical (Rim *vs.* 5ALA+) (B), Neural (Invasive-margin *vs.* Core) (C), and Proneural (5ALA- *vs.* 5ALA+) (D). Bar plot representing the number of NeuN positive cells in patient-matched spatially distinct GBM regions (Core, Rim, and Invasive-margin) across three GBM patients (E). The star (*) symbol indicates the p-value as estimated by the patient-matched paired T-test. Error bar shows standard deviation. Differential z-scored normalized expression Log2(TPM+1) of the leading-edge genes of GBM subtypes (from Verhaak *et al.*) – Classical (F) and Proneural (G) - are shown as a heatmap. Column (samples) and row (genes) are clustered by the Euclidian distance method. The color code represents the differential expression of genes (Yellow: higher expression; Blue = lower expression). The boxplots represent the $\log_2$ (TPM+1) expression levels for each brain region (Core, Rim, Invasive-margin) and 5ALA sorted cells (5ALA-, 5ALA+), with significant expression differences (Limma, padj < 0.05) denoted among GBM regions and 5ALA sorted cells for GBM subtypes – Classical (H), Neural (I), Proneural (J) and Mesenchymal (K).

Fig S4

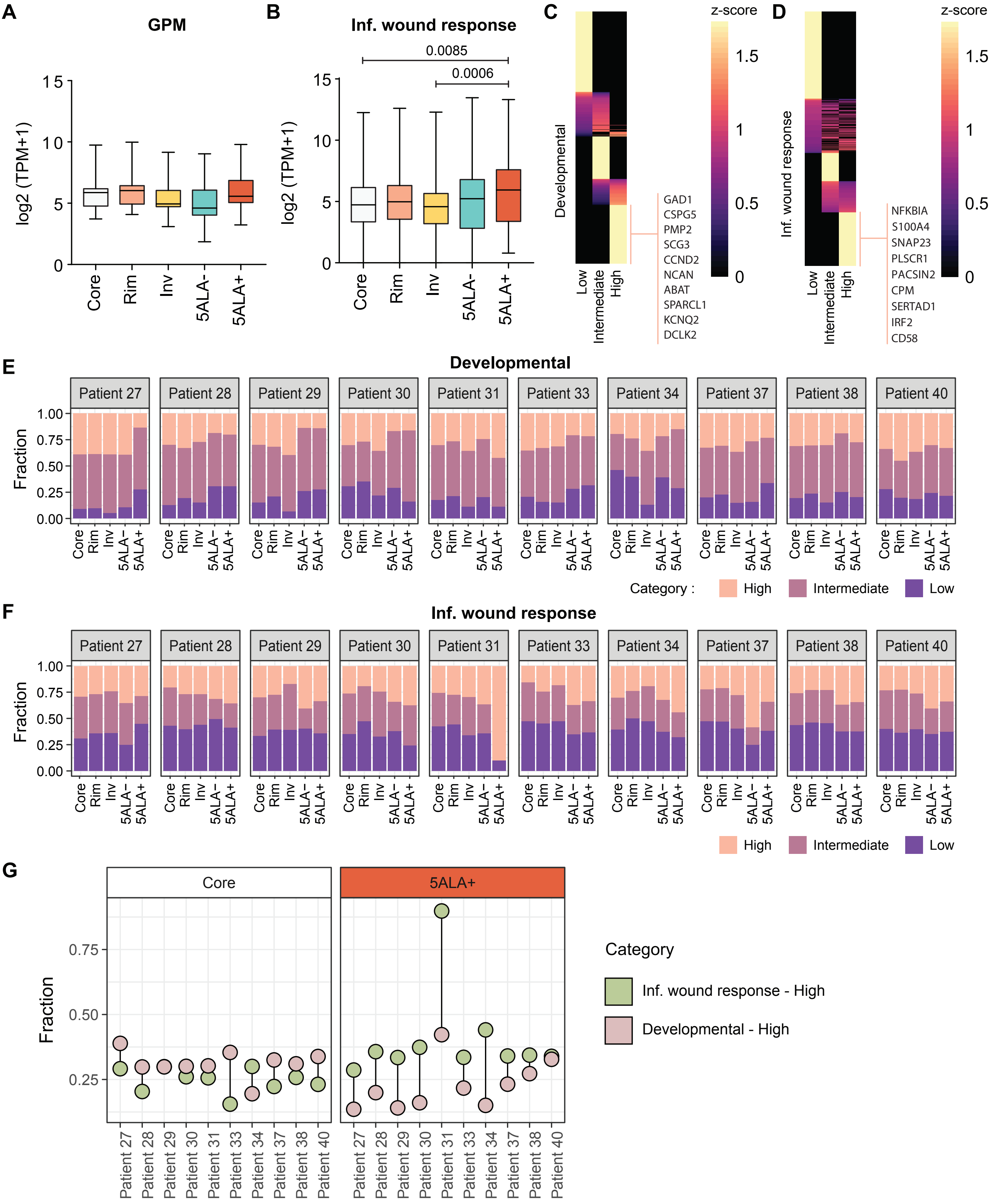
A
GPM
log2 (TPM+1)
Core
Rim
Inv
5ALA-
5ALA+
B
Inf. wound response
0.0085
0.0006
C
Developmental
Low
Intermediate
High
GAD1
CSPG5
PMP2
SCG3
CCND2
NCAN
ABAT
SPARCL1
KCNQ2
DCLK2
z-score
D
Inf. wound response
NFKBIA
S100A4
SNAP23
PLSCR1
PACSIN2
CPM
SERTAD1
IRF2
CD58
E
Developmental
Patient 27
Patient 28
Patient 29
Patient 30
Patient 31
Patient 33
Patient 34
Patient 37
Patient 38
Patient 40
Fraction
Category :
High
Intermediate
Low
F
Inf. wound response
G
Core
5ALA+
Category
Inf. wound response - High
Developmental - High

Fig. S4: Metabolic and transcriptional signature analysis and deconvolution of SPRP

Box plots showing the relative $\log_2$ (TPM+1) expression levels of differentially regulated (Limma; padj<0.05) cellular and metabolic state gene sets – GPM (A), Inf. wound response (B), for Core, Rim, Invasive-margin, 5ALA- and 5ALA+ cells. Respective p-values (if <0.05) are also shown. The signature-matrix generated from the single-cell RNA-seq data (Richards *et al.*) by CIBERSORTx deconvolution algorithm is shown. The matrix represents three categories (High, Intermediate, and Low) based on the expression of two transcriptional programs – Development (C) and Inf. wound response (D). Highly expressed canonical markers genes for Developmental-High and Inf. wound response-High are shown. Color code represents the differential expression (yellow: High, red: Low, Black: No expression). Patient-wise stacked bar plot representing the estimated fraction of cells with High, Intermediate, and Low transcriptional programs - Development (E) and Inf. wound response (F) across 10 GBM samples. The color indicates three different categories (High, Intermediate, and Low) of the transcriptional programs as reported by Neftel *et al.* (Development and Inf. wound response). The patient-wise estimated fractions of cells with High transcriptional programs (Development and Inf. wound response) in Core (left) and 5ALA+ cell (right) populations (G).

Fig S5

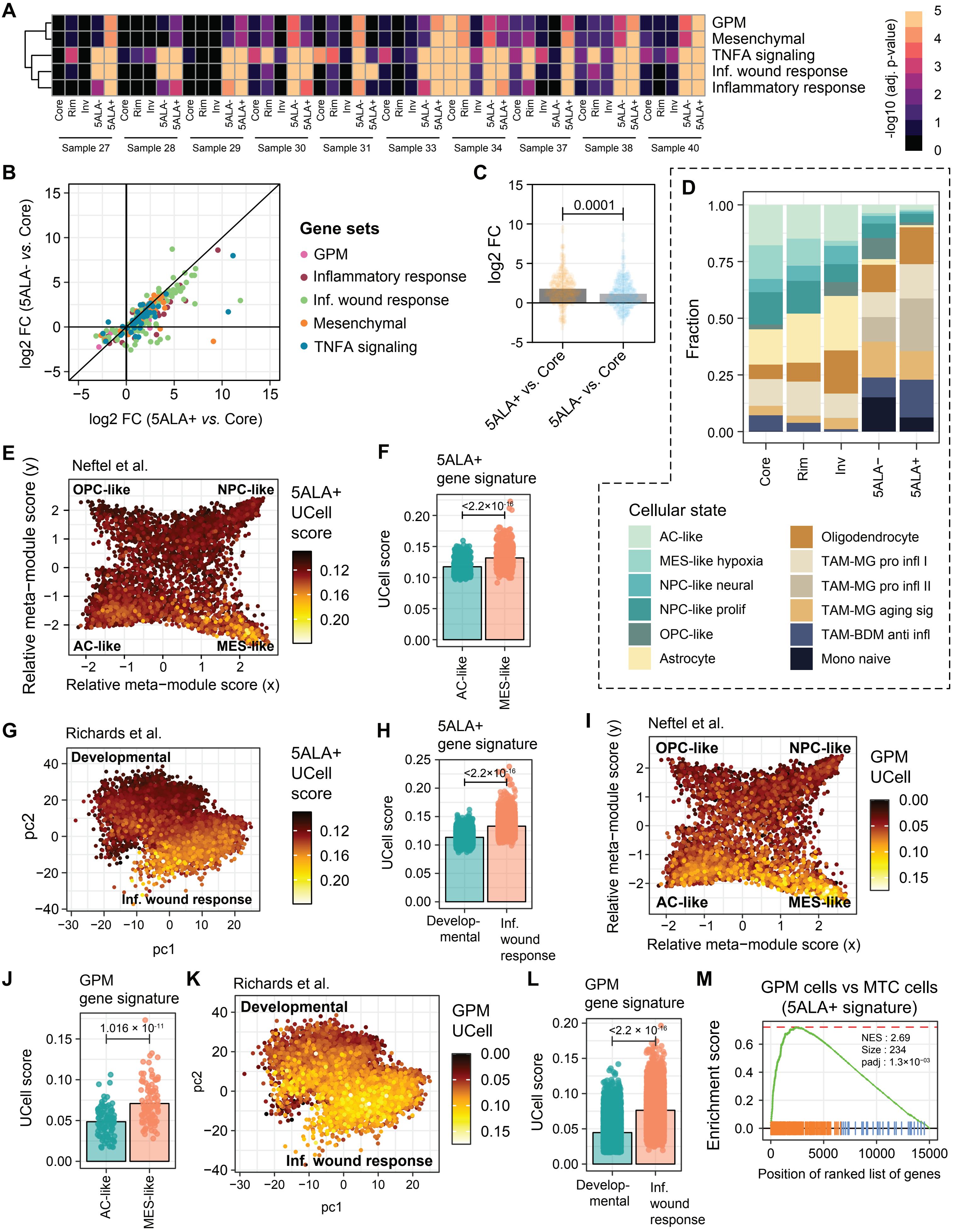

A
GPM
Mesenchymal
TNFA signaling
Inf. wound response
Inflammatory response
-log10 (adj. p-value)
Sample 27
Sample 28
Sample 29
Sample 30
Sample 31
Sample 33
Sample 34
Sample 37
Sample 38
Sample 40
Core
Rim
Inv
5ALA-
5ALA+
B
log2 FC (5ALA- vs. Core)
log2 FC (5ALA+ vs. Core)
Gene sets
GPM
Inflammatory response
Inf. wound response
Mesenchymal
TNFA signaling
C
log2 FC
0.0001
5ALA+ vs. Core
5ALA- vs. Core
D
Fraction
Cellular state
AC-like
MES-like hypoxia
NPC-like neural
NPC-like prolif
OPC-like
Astrocyte
Oligodendrocyte
TAM-MG pro infl I
TAM-MG pro infl II
TAM-MG aging sig
TAM-BDM anti infl
Mono naive
E
Neftel et al.
OPC-like
NPC-like
AC-like
MES-like
Relative meta−module score (y)
Relative meta−module score (x)
5ALA+ UCell score
F
5ALA+ gene signature
UCell score
<2.2×10-16
AC-like
MES-like
G
Richards et al.
Developmental
Inf. wound response
pc1
pc2
5ALA+ UCell score
H
5ALA+ gene signature
UCell score
<2.2×10-16
Develop-mental
Inf. wound response
I
Neftel et al.
OPC-like
NPC-like
AC-like
MES-like
Relative meta−module score (y)
Relative meta−module score (x)
GPM UCell
J
GPM gene signature
UCell score
1.016 × 10-11
AC-like
MES-like
K
Richards et al.
Developmental
Inf. wound response
pc1
pc2
GPM UCell
L
GPM gene signature
UCell score
<2.2 × 10-16
Develop-mental
Inf. wound response
M
GPM cells vs MTC cells (5ALA+ signature)
Enrichment score
NES : 2.69
Size : 234
padj : 1.3×10-03
Position of ranked list of genes

Fig. S5: Single-cell analysis from independent cohorts

Single sample GSEA (ssGSEA) was performed on distinct GBM regions (Core, Rim, Invasive-margin, 5ALA+, and 5ALA-) across ten GBM patients separately for five unique gene-signatures in 5ALA+ cells – GPM, MES, TNF-A signaling, Inflammatory wound response, and Inflammatory response (A). Log2 fold change (FC) of 5ALA+ genes between 5ALA+ vs. Core (x-axis) and 5ALA- vs. Core (y-axis) (B). Bar jitter plot showing the Log2 FCs (5ALA+ vs. Core and 5ALA- vs. Core) (C). The p-value was calculated by T-test between the FCs. The stacked bar plot represents the average cell-type estimates based on a GBmap data across 10 GBM samples; the color indicates different cell types (D). Two-dimensional scatter plot of cellular states based on RNA-seq dataset (Neftel *et al.*) where each quadrant corresponds to one distinct cellular state. The dots represent the positions of GBM cells in a two-dimensional plane reflecting their relative scores for the meta-modules - OPC-like, NPC-like, AC-like, and MES-like (E). The color code is based on the single-cell wise UCell scores of 5ALA+ gene signature across the GBM cells where yellow and black indicate high and low UCell scores, respectively. 5ALA+ scores were compared between MES-like and AC-like cells (F). The p-value (Wilcox test) is shown. Principle component analysis (PCA) plot of GSCs (N= 65,655 cells) based on RNA-seq dataset (Richards *et al.*) (G). Each dot represents a single GSC positioned by expression of Developmental (PC1-low) and Injury Response (PC1-high) programs. The color code is based on the single-cell wise UCell scores of 5ALA+ gene signature across the GBM cells where yellow and black indicate high and low UCell scores. 5ALA+ scores were compared between Developmental (N= 10,000) and Inf. wound response/Injury (N= 10,000) cells. The p-value = 2.2×10^{-16} (H). Mapping GPM UCell scores on the single cells from Netfel (I) and Richards *et al.* datasets (K). GPM scores were compared

between MES-like and AC-like cells. The p-value for this particular comparison was 1.016×10^{-11} (J). GPM scores were compared between Developmental (N= 10,000) and Inf. wound response/Injury (N= 10,000) cells. The p-value = 2.2×10^{-16} (L). Enrichment of 5ALA+ gene signature in GPM PDCs compared to MTC PDCs (M).

Fig S6

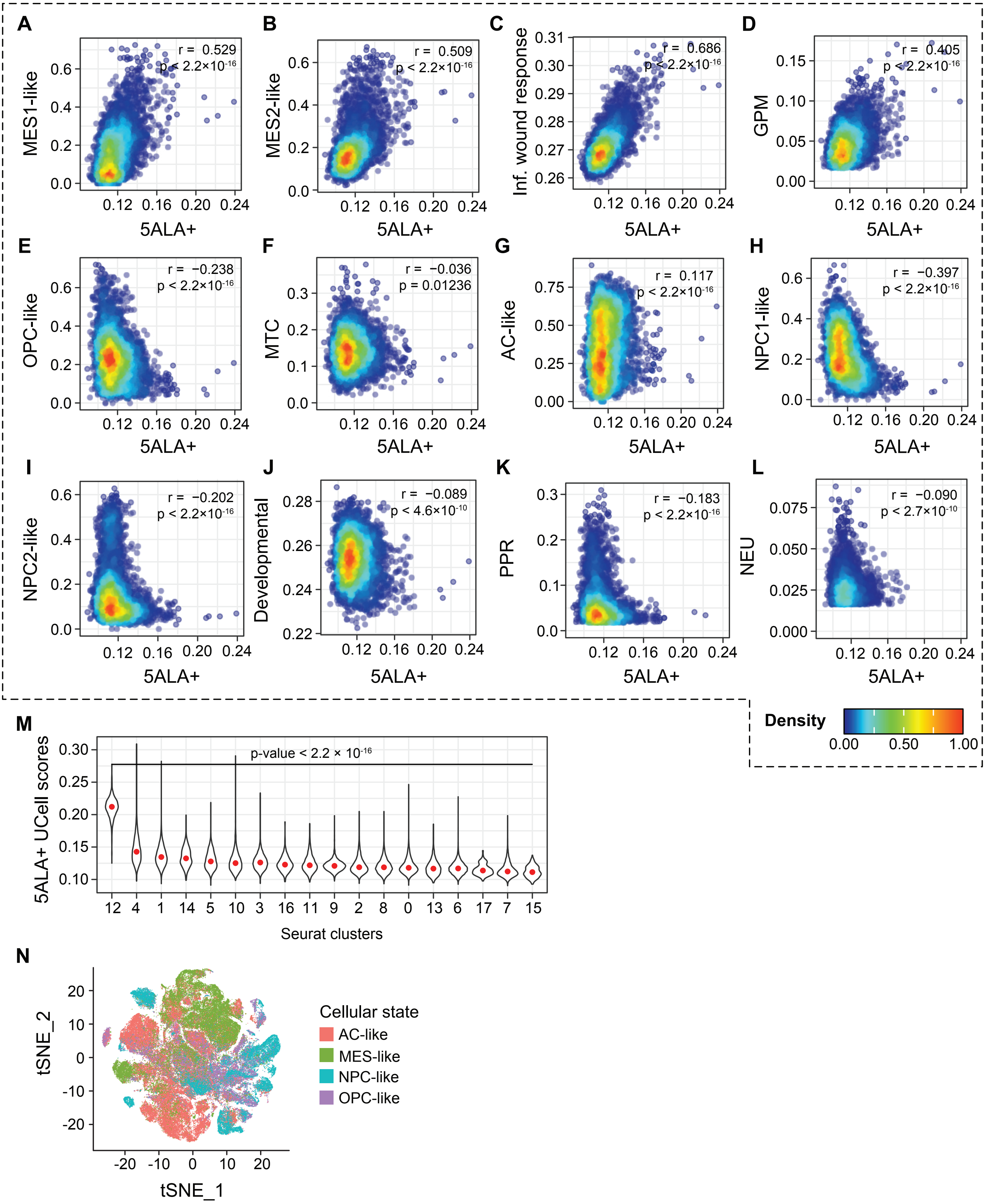

A
MES1-like
r = 0.529
p < 2.2×10-16
5ALA+
B
MES2-like
r = 0.509
p < 2.2×10-16
5ALA+
C
Inf. wound response
r = 0.686
p < 2.2×10-16
5ALA+
D
GPM
r = 0.405
p < 2.2×10-16
5ALA+
E
OPC-like
r = −0.238
p < 2.2×10-16
5ALA+
F
MTC
r = −0.036
p = 0.01236
5ALA+
G
AC-like
r = 0.117
p < 2.2×10-16
5ALA+
H
NPC1-like
r = −0.397
p < 2.2×10-16
5ALA+
I
NPC2-like
r = −0.202
p < 2.2×10-16
5ALA+
J
Developmental
r = −0.089
p < 4.6×10-10
5ALA+
K
PPR
r = −0.183
p < 2.2×10-16
5ALA+
L
NEU
r = −0.090
p < 2.7×10-10
5ALA+
Density
0.00
0.50
1.00
M
5ALA+ UCell scores
p-value < 2.2 × 10-16
12 4 1 14 5 10 3 16 11 9 2 8 0 13 6 17 7 15
Seurat clusters
N
tSNE_2
tSNE_1
Cellular state
AC-like
MES-like
NPC-like
OPC-like

Fig. S6: Single-cell wise UCell enrichment analysis

Scatter plots representing the UCell score correlation between 5ALA+ gene signature and MES1-like (A), MES2-like (B), Inf. wound response (C), GPM (D), OPC-like (E), and MTC (F), AC-like (G), NPC1-like (H), NPC2-like (I), Developmental (J), PPR (K), NEU (L). and. The Pearson correlation coefficient (r) and p-values are given for each scatter plot. P-values of correlation are given. Average 5ALA+ UCell scores across different Louvain clusters (M). We performed bootstrap analysis where 100 cells were selected randomly 1000 times for the comparison followed by the Wilcox test. The median p-value between cluster-12 and cluster-4 was 2.2×10^{-16}. All the other comparisons between Cluster-2 and the rest of the clusters resulted in p-value below 2.2×10^{-16}. Cell state annotations of the tSNE plot based on neoplastic cells from the GBmap dataset (N).

Fig S7

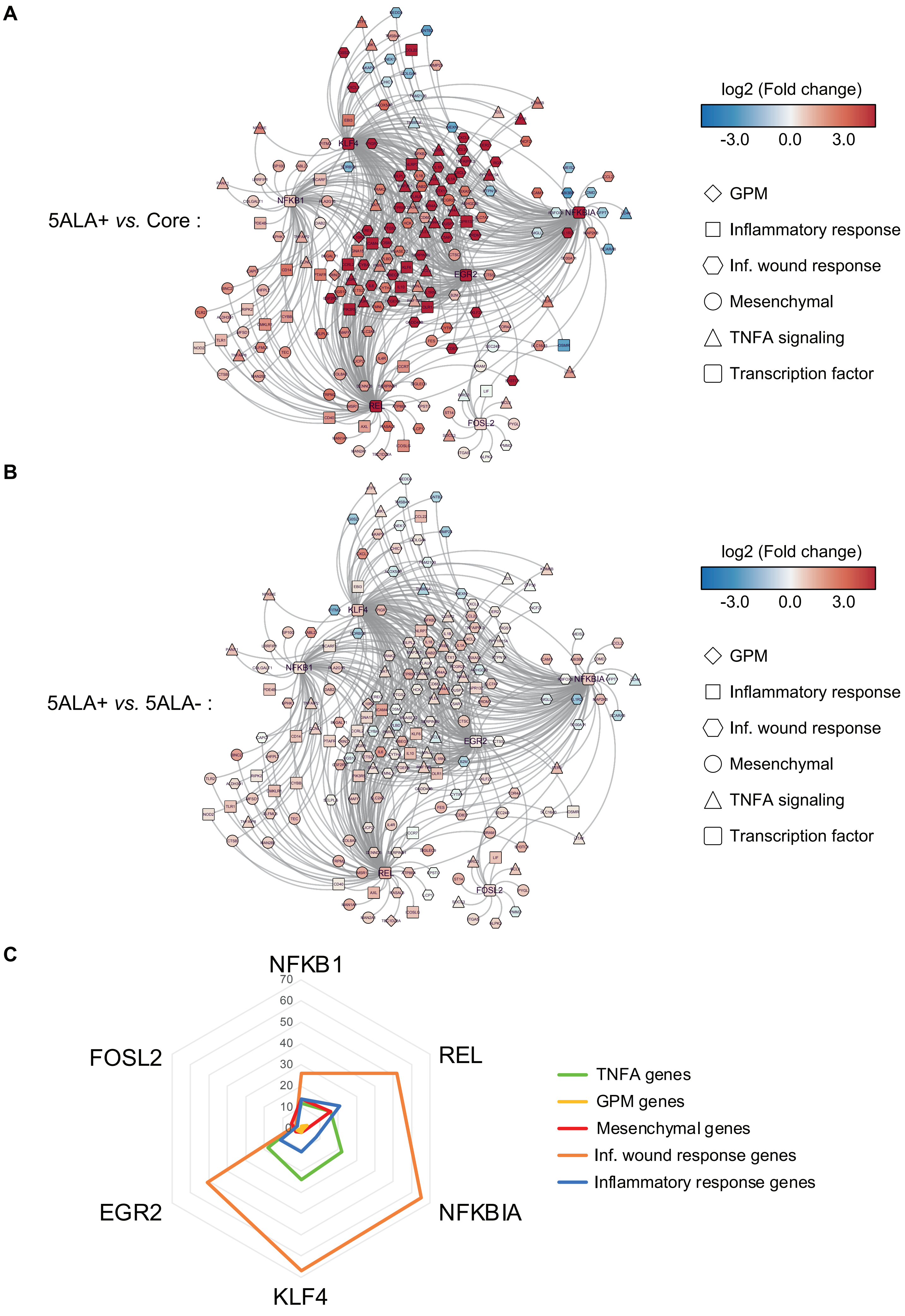
A
5ALA+ vs. Core :
B
5ALA+ vs. 5ALA- :
KLF4
NFKB1
NFKBIA
EGR2
REL
FOSL2
log2 (Fold change)
-3.0
0.0
3.0
GPM
Inflammatory response
Inf. wound response
Mesenchymal
TNFA signaling
Transcription factor
C
NFKB1
REL
NFKBIA
KLF4
EGR2
FOSL2
70
60
50
40
30
20
10
0
TNFA genes
GPM genes
Mesenchymal genes
Inf. wound response genes
Inflammatory response genes

Fig. S7: Transcription factor – Target gene network analysis

Transcription networks upregulated in 5ALA+ cells in comparison to Core (A) and 5ALA- cells (B) are shown. Transcription factors (TF) were identified in the differentially regulated leading-edge genes that were enriched in 5ALA+ cells by using ENCODE TF-annotation. The association between TF and corresponding target genes (TG) was retrieved from the ENCODE database. A transcription network is constructed representing TF-TG association by using the ARACNE algorithm. The diagram illustrates that TGs (Nodes) associated with enriched gene sets (Inflammatory response, Inflammatory wound response, MTC, Mesenchymal subtype, GPM, and TNF-A signaling), are mutually controlled by different TFs (Hubs). The association between a node (TG) and a hub (TF) is shown as edges (grey curved lines) and nodes/hubs are color-coded according to the Log_2 fold change (FC) values between 5ALA+ and Core (A) and 5ALA- cells (B), where red and blue indicate higher and lower FCs respectively. A radar plot representing TG (of different enriched subtypes) counts for the TFs (C). The colored dots represent the TG count for a specific TF.

Fig S8

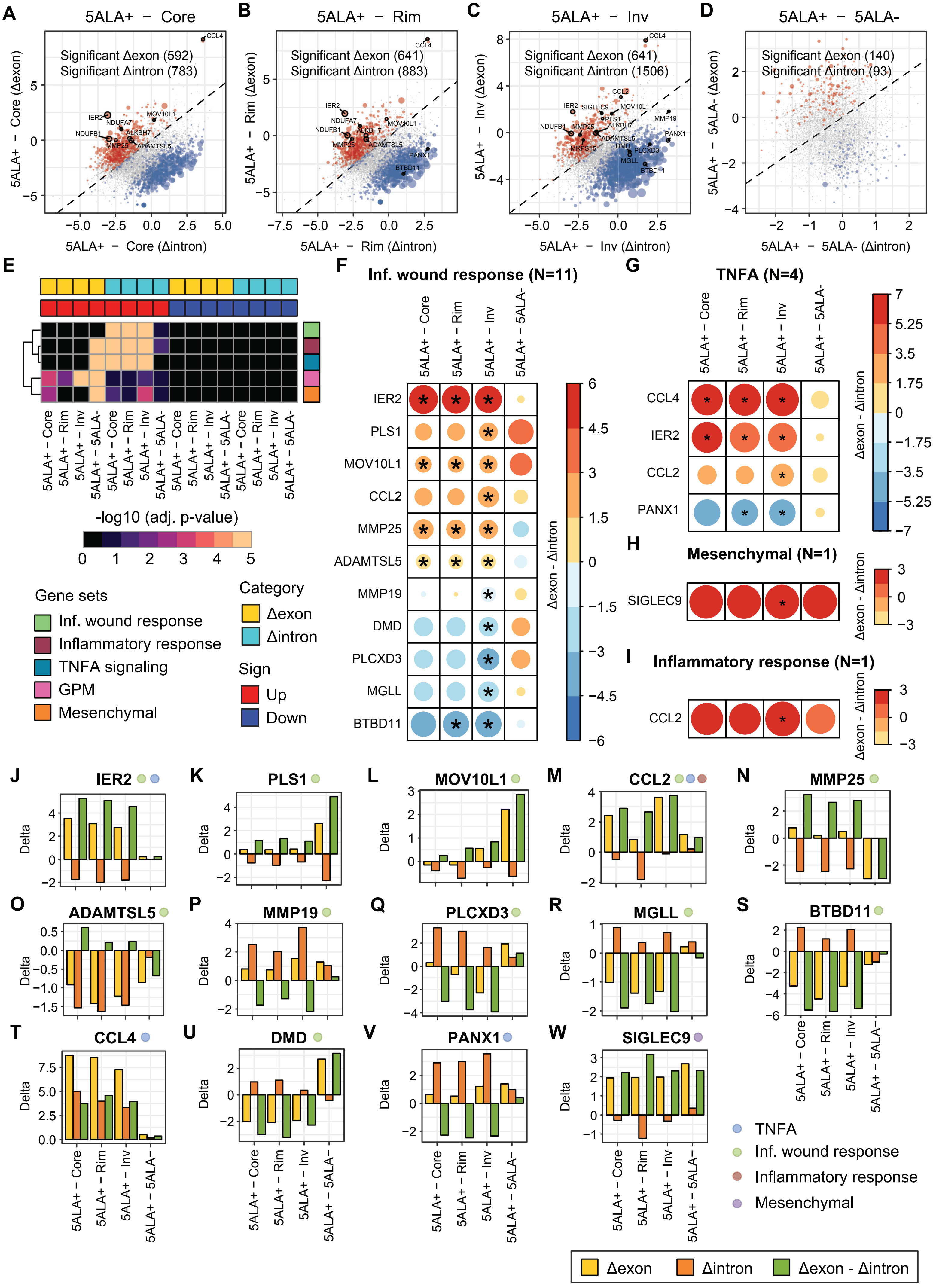

A
5ALA+ – Core
Significant Δexon (592)
Significant Δintron (783)
5ALA+ – Core (Δexon)
5ALA+ – Core (Δintron)
B
5ALA+ – Rim
Significant Δexon (641)
Significant Δintron (883)
5ALA+ – Rim (Δexon)
5ALA+ – Rim (Δintron)
C
5ALA+ – Inv
Significant Δexon (641)
Significant Δintron (1506)
5ALA+ – Inv (Δexon)
5ALA+ – Inv (Δintron)
D
5ALA+ – 5ALA-
Significant Δexon (140)
Significant Δintron (93)
5ALA+ – 5ALA- (Δexon)
5ALA+ – 5ALA- (Δintron)
E
-log10 (adj. p-value)
Gene sets
Inf. wound response
Inflammatory response
TNFA signaling
GPM
Mesenchymal
Category
Δexon
Δintron
Sign
Up
Down
F
Inf. wound response (N=11)
IER2
PLS1
MOV10L1
CCL2
MMP25
ADAMTSL5
MMP19
DMD
PLCXD3
MGLL
BTBD11
Δexon - Δintron
G
TNFA (N=4)
CCL4
IER2
CCL2
PANX1
H
Mesenchymal (N=1)
SIGLEC9
I
Inflammatory response (N=1)
CCL2
J
IER2
K
PLS1
L
MOV10L1
M
CCL2
N
MMP25
O
ADAMTSL5
P
MMP19
Q
PLCXD3
R
MGLL
S
BTBD11
T
CCL4
U
DMD
V
PANX1
W
SIGLEC9
Delta
5ALA+ – Core
5ALA+ – Rim
5ALA+ – Inv
5ALA+ – 5ALA-
TNFA
Inf. wound response
Inflammatory response
Mesenchymal
Δexon
Δintron
Δexon - Δintron

Fig. S8: Exon-intron split analysis

Exon-intron split analysis (EISA) of enriched cellular and metabolic subtypes in 5ALA+ cells. Scatter plot showing the gene-specific changes in exon (delta-exon) and intron (delta-intron) counts between 5ALA+ cells and tumor Core (A), Rim (B), Invasive-margin (C) and 5ALA- cells (D). X- and Y-axis represent the delta-intron and delta-exon, respectively. Color code represents the genes that showed significant regulation with adjusted p-value<0.05 (Blue: a significant change in delta intron and Red: a significant change in delta exon) between 5ALA+ cells and intra-tumor regions or 5ALA- cells. Heatmap showing the –log10 adjusted p-value of the hallmark enriched pathways (E). The samples representing the comparisons of 5ALA+ with other regions are given in columns (5ALA+ *vs.* tumor Core; 5ALA+ *vs.* Rim; 5ALA+ *vs.* Invasive-margin; and 5ALA+ *vs.* 5ALA). Each row represents the different cellular and metabolic subtypes. Columns are divided into upregulated (Red) or downregulated (Blue) segments based on the exon and intron regulation of genes in 5ALA+ cells. Finally, delta-exon (yellow) and delta-intron (Light blue) are annotated for each column. The color code in the heatmap is representative of –log10 p-values (Orange is highly significant and Black is not significant). Regulated DEGs with a significant change in exon and intron counts are shown as heatmaps for Inflammatory wound response (F), TNF-α signaling (G), Mesenchymal subtype (H), and Inflammatory response (I). The color code represents the difference between delta exon and delta intron (Δexon-Δintron). The significant changes of Δexon-Δintron are shown as (*). The size of the circle is according to the adjusted –log10 p-value. Bar plots represent delta exon (Δexon), delta intron (Δintron), and the difference between Δexon and Δintron (Δexon - Δintron) for the significant DEGs (J-W). The circles adjacent to the gene symbol represent the gene-signature and the colors of the circle indicate different gene-signatures.

Fig S9

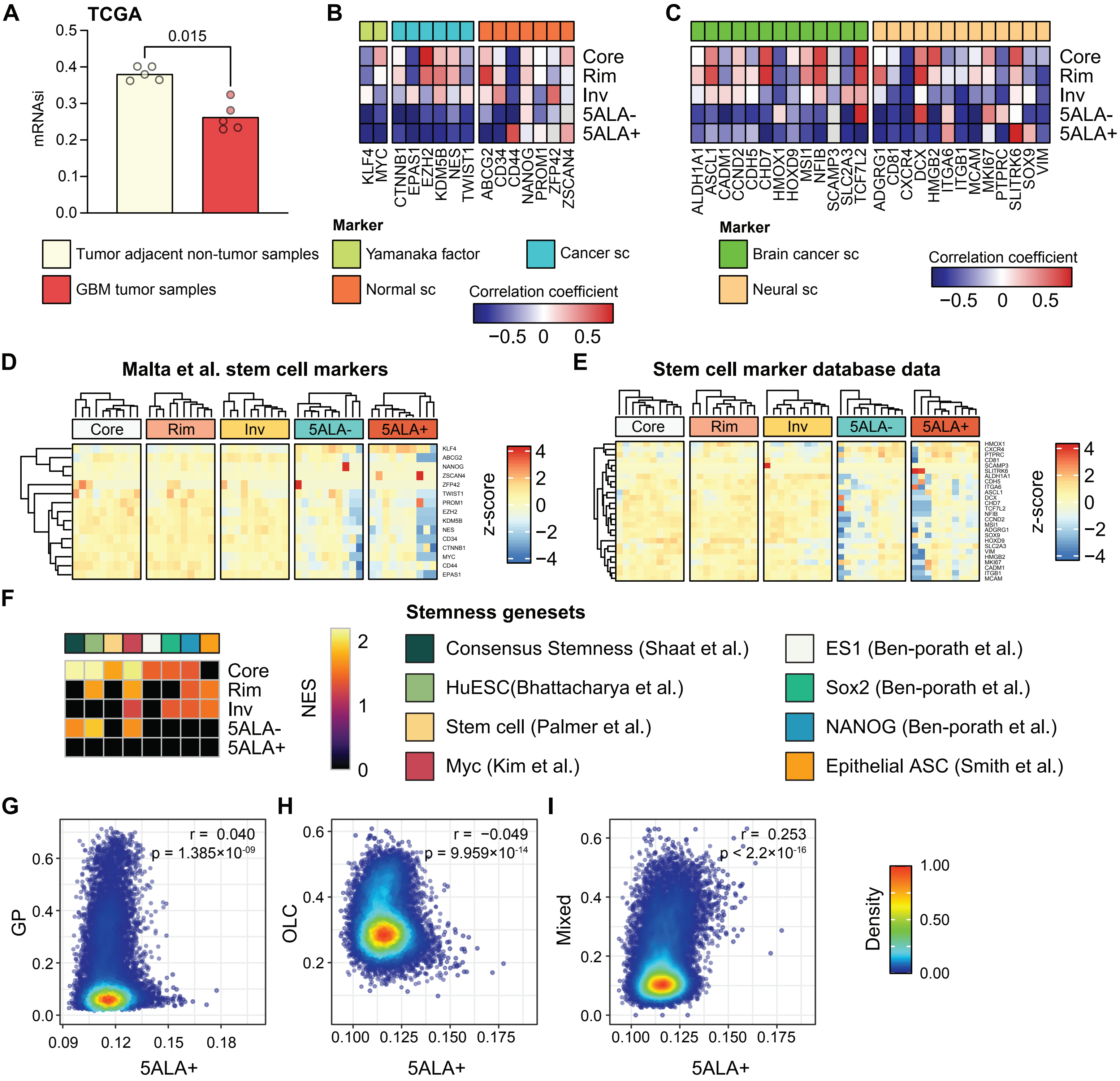

A
TCGA
0.015
mRNAsi
0.5
0.4
0.3
0.2
0.1
0.0
Tumor adjacent non-tumor samples
GBM tumor samples
B
Core
Rim
Inv
5ALA-
5ALA+
KLF4
MYC
CTNNB1
EPAS1
EZH2
KDM5B
NES
TWIST1
ABCG2
CD34
CD44
NANOG
PROM1
ZFP42
ZSCAN4
Marker
Yamanaka factor
Cancer sc
Normal sc
Correlation coefficient
−0.5 0 0.5
C
Core
Rim
Inv
5ALA-
5ALA+
ALDH1A1
ASCL1
CADM1
CCND2
CDH5
CHD7
HMOX1
HOXD9
MSI1
NFIB
SCAMP3
SLC2A3
TCF7L2
ADGRG1
CD81
CXCR4
DCX
HMGB2
ITGA6
ITGB1
MCAM
MKI67
PTPRC
SLITRK6
SOX9
VIM
Marker
Brain cancer sc
Neural sc
Correlation coefficient
−0.5 0 0.5
D
Malta et al. stem cell markers
Core
Rim
Inv
5ALA-
5ALA+
z-score
4
2
0
−2
−4
E
Stem cell marker database data
Core
Rim
Inv
5ALA-
5ALA+
z-score
4
2
0
−2
−4
F
Core
Rim
Inv
5ALA-
5ALA+
NES
2
1
0
Stemness genesets
Consensus Stemness (Shaat et al.)
HuESC(Bhattacharya et al.)
Stem cell (Palmer et al.)
Myc (Kim et al.)
ES1 (Ben-porath et al.)
Sox2 (Ben-porath et al.)
NANOG (Ben-porath et al.)
Epithelial ASC (Smith et al.)
G
r = 0.040
p = 1.385×10-09
GP
0.6
0.4
0.2
0.0
0.09
0.12
0.15
0.18
5ALA+
H
r = −0.049
p = 9.959×10-14
OLC
0.6
0.4
0.2
0.100
0.125
0.150
0.175
5ALA+
I
r = 0.253
p < 2.2×10-16
Mixed
0.6
0.4
0.2
0.0
0.100
0.125
0.150
0.175
5ALA+
Density
1.00
0.50
0.00

Fig. S9: Stemness signature analysis

Comparison of mRNAsi of adjacent non-tumor tissue (NAT) and GBM tumor tissue (A). P-value is calculated by the Wilcox test following a bootstrap analysis where an equal number of samples from GBM tissue were drawn 100 times to compare with NAT. The median p-value of 100 comparisons is shown. Correlation between mRNAsi values and mRNA expression for available Malta *et al.* stem cell markers (B). Correlation between mRNAsi and mRNA expression for markers from stem cell marker database (C). Heatmap of RNA-seq expression z-scores for the Malta *et al.* stem cell marker signatures (D) and markers from stem cell marker database (E). Heatmap illustrating normalized enrichment scores (NES) of GSEA with padj<0.05 for selected stemness gene-sets from different studies (F). Scatter plots representing the UCell score correlation between gene signature of 5ALA+ cells and GP (G), OLC (H), and Others mixed population (I). The Pearson correlation coefficient (r) is given for each scatter plot. P-values of correlation are given.

Fig S10

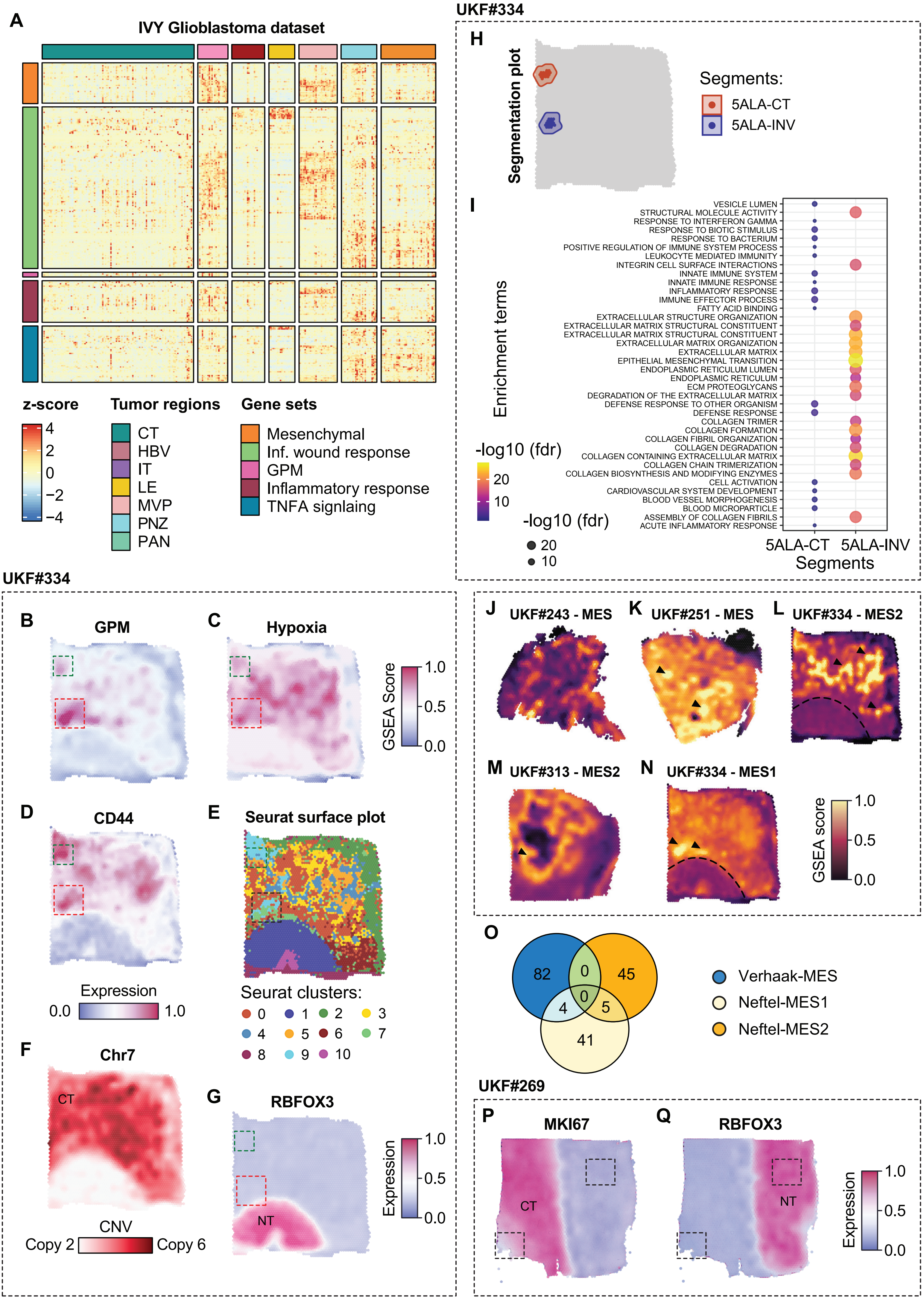
A
IVY Glioblastoma dataset
z-score
4
2
0
−2
−4
Tumor regions
CT
HBV
IT
LE
MVP
PNZ
PAN
Gene sets
Mesenchymal
Inf. wound response
GPM
Inflammatory response
TNFA signlaing
UKF#334
H
Segmentation plot
Segments:
5ALA-CT
5ALA-INV
I
Enrichment terms
VESICLE LUMEN
STRUCTURAL MOLECULE ACTIVITY
RESPONSE TO INTERFERON GAMMA
RESPONSE TO BIOTIC STIMULUS
RESPONSE TO BACTERIUM
POSITIVE REGULATION OF IMMUNE SYSTEM PROCESS
LEUKOCYTE MEDIATED IMMUNITY
INTEGRIN CELL SURFACE INTERACTIONS
INNATE IMMUNE SYSTEM
INNATE IMMUNE RESPONSE
INFLAMMATORY RESPONSE
IMMUNE EFFECTOR PROCESS
FATTY ACID BINDING
EXTRACELLULAR STRUCTURE ORGANIZATION
EXTRACELLULAR MATRIX STRUCTURAL CONSTITUENT
EXTRACELLULAR MATRIX STRUCTURAL CONSTITUENT
EXTRACELLULAR MATRIX ORGANIZATION
EXTRACELLULAR MATRIX
EPITHELIAL MESENCHYMAL TRANSITION
ENDOPLASMIC RETICULUM LUMEN
ENDOPLASMIC RETICULUM
ECM PROTEOGLYCANS
DEGRADATION OF THE EXTRACELLULAR MATRIX
DEFENSE RESPONSE TO OTHER ORGANISM
DEFENSE RESPONSE
COLLAGEN TRIMER
COLLAGEN FORMATION
COLLAGEN FIBRIL ORGANIZATION
COLLAGEN DEGRADATION
COLLAGEN CONTAINING EXTRACELLULAR MATRIX
COLLAGEN CHAIN TRIMERIZATION
COLLAGEN BIOSYNTHESIS AND MODIFYING ENZYMES
CELL ACTIVATION
CARDIOVASCULAR SYSTEM DEVELOPMENT
BLOOD VESSEL MORPHOGENESIS
BLOOD MICROPARTICLE
ASSEMBLY OF COLLAGEN FIBRILS
ACUTE INFLAMMATORY RESPONSE
-log10 (fdr)
20
10
-log10 (fdr)
20
10
5ALA-CT
5ALA-INV
Segments
UKF#334
B
GPM
C
Hypoxia
GSEA Score
1.0
0.5
0.0
D
CD44
E
Seurat surface plot
Expression
0.0
1.0
Seurat clusters:
0
1
2
3
4
5
6
7
8
9
10
F
Chr7
CT
CNV
Copy 2
Copy 6
G
RBFOX3
NT
Expression
1.0
0.5
0.0
J
UKF#243 - MES
K
UKF#251 - MES
L
UKF#334 - MES2
M
UKF#313 - MES2
N
UKF#334 - MES1
GSEA score
1.0
0.5
0.0
O
82
0
45
4
0
5
41
Verhaak-MES
Neftel-MES1
Neftel-MES2
UKF#269
P
MKI67
CT
Q
RBFOX3
NT
Expression
1.0
0.5
0.0

Fig. S10: Spatial segmentation and differential gene expression analysis

Heatmap representing the enrichment of the 5ALA+ specific genes across different anatomical regions (CT: Cellular Tumor; HBV: Hyperplastic blood vessels; IT: Infiltrative tumor; LE: Leading-edge; MVP: Microvascular proliferation; PNZ; Perinecrotic zone, and PAN: Pseudopalisading cells around necrosis) based on the IVY Glioblastoma dataset (A). GSEA score of GPM (B), Hypoxia (C), and single gene score (z-scored) for *CD44* (D) are shown for sample UKF#334, where the enriched and random spots are shown in red and blue, respectively. The spatial localization of the enriched spots is marked by a dashed box where the black and green box represents the 5ALA+ spots within the invasive margin (5ALA-INV) and inside the cellular tumor (5ALA-CT), respectively. Seurat Clusters representing the transcriptional diversities are mapped in the tissue section based on Spatial Transcriptomics data (E). Color codes are indicative of different clusters. The Inferred CNV analysis of Chromosome-7 (F) is presented as a spatial surface plot where the gain of Chromosome-7 is color-coded. The z-scored gene-expression value for *RBFOX3* (G) is mapped and represented as a surface plot. The spatial locations of the segmented 5ALA-INV and 5ALA-CT spots are shown (H) where 5ALA-INV and 5ALA-CT segments are represented by blue and red colors. GSEA scores of the enriched pathways based on the top-20 differentially expressed genes between the segmented 5ALA-INV and 5ALA-CT regions are shown as a heatmap (I). The color code is based on enrichment score: $-\log_{10}$(FDR) values. Spatial Gene-set enrichment analysis of MES, MES1, and MES2 genes across GBM tissues (J-N). Venn Diagram showing minimal or no overlap among MES, MES1, and MES2 gene sets (O). Spatial expressions (z-scored) of *MIK67* (Ki67) (P) and *RBFOX3* (NeuN) (Q) genes in sample UKF#269 are shown as surface plots.

Fig S11

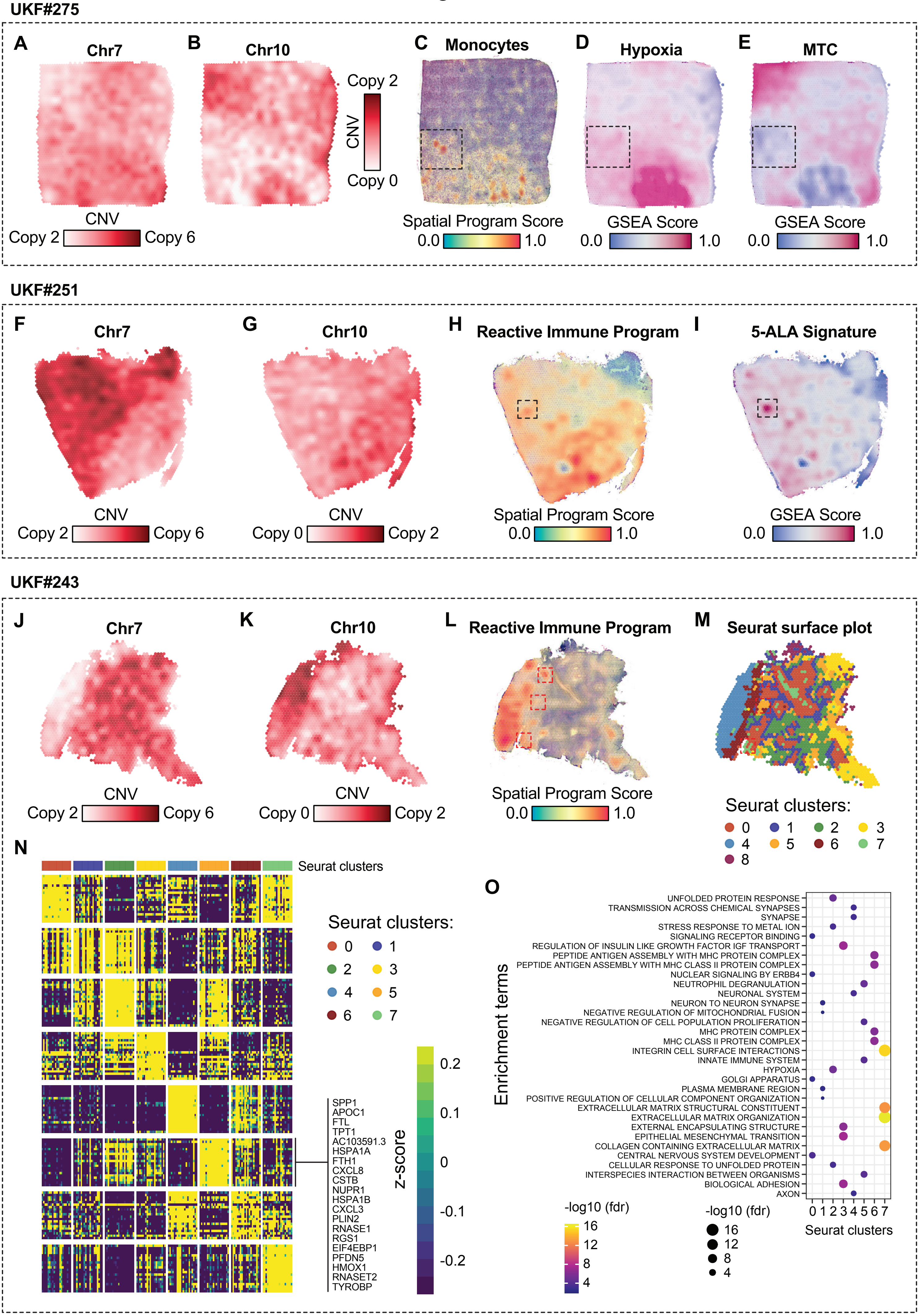
UKF#275
A
Chr7
B
Chr10
CNV
Copy 2
Copy 6
Copy 0
C
Monocytes
Spatial Program Score
0.0
1.0
D
Hypoxia
GSEA Score
E
MTC
UKF#251
F
Chr7
G
Chr10
H
Reactive Immune Program
I
5-ALA Signature
UKF#243
J
Chr7
K
Chr10
L
Reactive Immune Program
M
Seurat surface plot
Seurat clusters:
0 1 2 3 4 5 6 7 8
N
Seurat clusters
SPP1
APOC1
FTL
TPT1
AC103591.3
HSPA1A
FTH1
CXCL8
CSTB
NUPR1
HSPA1B
CXCL3
PLIN2
RNASE1
RGS1
EIF4EBP1
PFDN5
HMOX1
RNASET2
TYROBP
z-score
0.2
0.1
0
-0.1
-0.2
O
Enrichment terms
UNFOLDED PROTEIN RESPONSE
TRANSMISSION ACROSS CHEMICAL SYNAPSES
SYNAPSE
STRESS RESPONSE TO METAL ION
SIGNALING RECEPTOR BINDING
REGULATION OF INSULIN LIKE GROWTH FACTOR IGF TRANSPORT
PEPTIDE ANTIGEN ASSEMBLY WITH MHC PROTEIN COMPLEX
PEPTIDE ANTIGEN ASSEMBLY WITH MHC CLASS II PROTEIN COMPLEX
NUCLEAR SIGNALING BY ERBB4
NEUTROPHIL DEGRANULATION
NEURONAL SYSTEM
NEURON TO NEURON SYNAPSE
NEGATIVE REGULATION OF MITOCHONDRIAL FUSION
NEGATIVE REGULATION OF CELL POPULATION PROLIFERATION
MHC PROTEIN COMPLEX
MHC CLASS II PROTEIN COMPLEX
INTEGRIN CELL SURFACE INTERACTIONS
INNATE IMMUNE SYSTEM
HYPOXIA
GOLGI APPARATUS
PLASMA MEMBRANE REGION
POSITIVE REGULATION OF CELLULAR COMPONENT ORGANIZATION
EXTRACELLULAR MATRIX STRUCTURAL CONSTITUENT
EXTRACELLULAR MATRIX ORGANIZATION
EXTERNAL ENCAPSULATING STRUCTURE
EPITHELIAL MESENCHYMAL TRANSITION
COLLAGEN CONTAINING EXTRACELLULAR MATRIX
CENTRAL NERVOUS SYSTEM DEVELOPMENT
CELLULAR RESPONSE TO UNFOLDED PROTEIN
INTERSPECIES INTERACTION BETWEEN ORGANISMS
BIOLOGICAL ADHESION
AXON
-log10 (fdr)
16
12
8
4
0 1 2 3 4 5 6 7
Seurat clusters

Fig. S11: Spatial copy number variation and enrichment analysis

Inferred CNV analysis of Chromosome-7 (A) and -10 (B) are presented as spatial surface plots where the gain and loss of Chromosome-7 and -10, respectively are color-coded for sample UKF#275. GSEA scores of monocyte (C), Hypoxia (D), and MTC (E) are represented as surface plots by showing the enriched and random spots in red and blue, respectively. For sample UKF#251 the inferred CNVs of Chromosome-7 (F) and -10 (G) are shown. The spatial location of the enrichment of the Reactive immune program (RIP) (H) and 5ALA+ gene-signature are shown (I). For sample UKF#243, the inferred CNVs of Chromosome-7 (J) and -10 (K) are shown. The spatial localization of the spots enriched with RIP (Red color) is presented as a surface plot (L). Seurat Clusters representing the transcriptional diversities are mapped in the tissue section based on Spatial Transcriptomics data (M). Color codes are indicative of different clusters. Heatmap represents the top20 differential genes of each Seurat cluster (N). The genes belonging to Cluster-5 are shown. The color code indicates the differential expression of the genes. GSEA scores of the enriched pathways based on the top-20 differentially expressed genes across the Seurat clusters are shown as a heatmap (O). The color code is based on enrichment score: $-\log_{10}$(FDR) values.

Fig S12

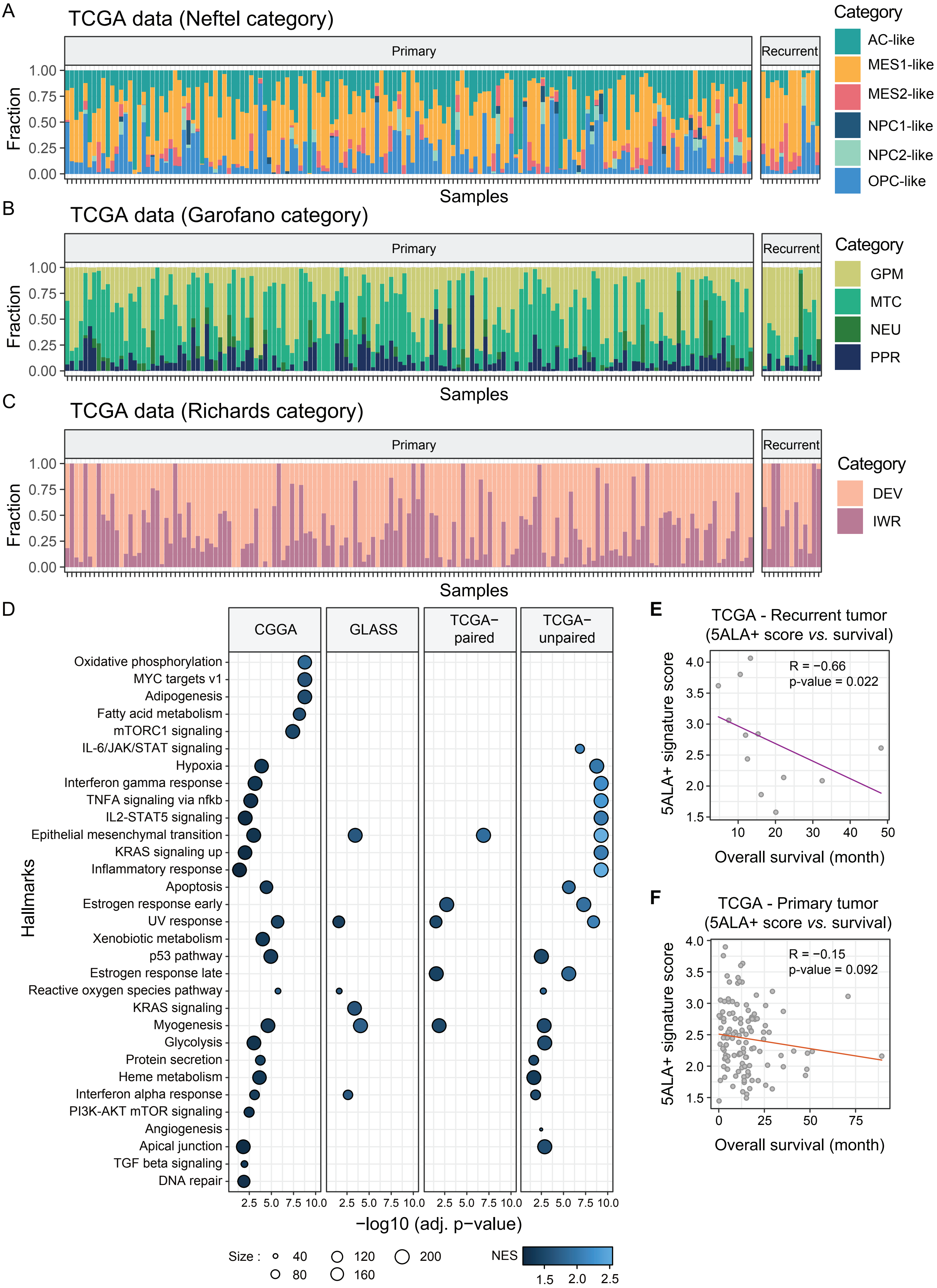
A
TCGA data (Neftel category)
Primary
Recurrent
Fraction
1.00
0.75
0.50
0.25
0.00
Samples
Category
AC-like
MES1-like
MES2-like
NPC1-like
NPC2-like
OPC-like
B
TCGA data (Garofano category)
Primary
Recurrent
Fraction
Samples
Category
GPM
MTC
NEU
PPR
C
TCGA data (Richards category)
Primary
Recurrent
Fraction
Samples
Category
DEV
IWR
D
CGGA
GLASS
TCGA−paired
TCGA−unpaired
Hallmarks
Oxidative phosphorylation
MYC targets v1
Adipogenesis
Fatty acid metabolism
mTORC1 signaling
IL-6/JAK/STAT signaling
Hypoxia
Interferon gamma response
TNFA signaling via nfkb
IL2-STAT5 signaling
Epithelial mesenchymal transition
KRAS signaling up
Inflammatory response
Apoptosis
Estrogen response early
UV response
Xenobiotic metabolism
p53 pathway
Estrogen response late
Reactive oxygen species pathway
KRAS signaling
Myogenesis
Glycolysis
Protein secretion
Heme metabolism
Interferon alpha response
PI3K-AKT mTOR signaling
Angiogenesis
Apical junction
TGF beta signaling
DNA repair
2.5 5.0 7.5 10.0
−log10 (adj. p−value)
Size : 40 80 120 160 200
NES 1.5 2.0 2.5
E
TCGA - Recurrent tumor
(5ALA+ score vs. survival)
R = −0.66
p-value = 0.022
5ALA+ signature score
Overall survival (month)
F
TCGA - Primary tumor
(5ALA+ score vs. survival)
R = −0.15
p-value = 0.092
5ALA+ signature score
Overall survival (month)

Fig. S12: CIBERSORTx derived Cell-type fraction estimation across primary and recurrent GBM patients

CIBERSORTx derived Cell-type fraction estimation across primary and recurrent GBM patients based on signature matrices from Netfel (A), Garofano (B), and Richards (C) *et al.* Hallmark enrichment analysis of DEGs between recurrent and primary GBM tumors. The hallmarks that are enriched in recurrent tumors in comparison to primary tumors from TCGA, CCGA, and GLASS cohorts are illustrated (D). Each bubble indicates one particular hallmark (y-axis). The x-axis indicates a –log10 adjusted p-value. The color gradient of the bubble indicates the normalized enrichment score (NES) whereas the size of the bubble depends on the gene-count for each hallmark. Paired and unpaired analysis was performed for the TCGA cohort. Scatter plots representing the correlation between 5ALA+ gene-signature scores and overall survival (months) in recurrent and primary patients from TCGA (E and F) cohort. Spearman correlation coefficient (R) and p-values are shown.
